# Effects of hearing intervention on blood-based biomarkers of neuroinflammation and neurodegeneration: a secondary analysis of the ACHIEVE randomised controlled trial

**DOI:** 10.1016/j.eclinm.2026.104164

**Published:** 2026-09-03

**Authors:** James Russell Pike, Alison R. Huang, Nicholas Reed, Michelle L. Arnold, Theresa H. Chisolm, David Couper, Jennifer A. Deal, Sarah Faucette, Adele M. Goman, Rebecca F. Gottesman, Timothy M. Hughes, David Knopman, Christine Mitchell, Thomas H. Mosley, Priya Palta, James S. Pankow, Victoria A. Sanchez, Elizabeth Selvin, Kevin J. Sullivan, Bharat Thyagarajan, Lynne E. Wagenknecht, Frank R. Lin, Josef Coresh, ACHIEVE Collaborative Research Group, Jennifer A. Deal, Frank R. Lin, Marilyn Albert, Joshua Betz, Alden Gross, Christine M. Mitchell, Clarice Myers, Jennifer A. Schrack, Richey Sharrett, Josef Coresh, James R. Pike, Nicholas S. Reed, Alison R. Huang, Melissa Minotti, Spencer Bolton, Laura Sherry, James S. Pankow, Sarah Aguilar, Elizabeth Anderson, Sydney Boelter, Elizabeth Penland Miller, Debbie Ng, Kristi Oeding, Sandra Potter, Katherine Teece, Soni Uccellini, Matthew Waggenspack, Luanne Welch, Jacqueline Weycker, Kerry Witherell, Kevin Sullivan, Thomas Mosley, Latilraka Anderson, Jillian Burt, April Carr, Arkenya Carter, Sarah Faucette, Rachel Foster, Ceola Greenwood, Temeka Griffin, Candace Jones, Dawn McLendon, Thomas Mosley, Stacee Naylor, Jenny Newman, Deidre O’Connor, Tiffany Owens, Jeraline Sims, Allison Thweatt, Tamikia Washington, Lynne E. Wagenknecht, Kathleen M. Hayden, Bria Backman, Debbie Barr, Joshua Evans, Jaime Hampton, Hailley Humphrey-Rutledge, Kaila H. Liou, Ashely Mitchell, Susan Smith, Nadine Shelton, Theresa Chisolm, Victoria Sanchez, Michelle Arnold, Ann C. Eddins, Emily Moore, Haley Neil, Preyanca Oree, Laura Westermann, Nancy W. Glynn, Yurun Cai, Theresa Gmelin, David Couper, Sheila Burgard, Lisa Gravens-Mueller, David Li, Adele M. Goman, Clifford R. Jack, David Knopman, Denise Reyes, A.J. Spychalla, Kaely Thostenson, Doug Galasko, Julie Buring, Judy Dubno, Tom Greene, Lawrence Lustig, Coryse St. Hillaire-Clarke, Matt Sutterer

**Affiliations:** a Department of Medicine, New York University Grossman School of Medicine, New York, NY, USA; b Department of Population Health, New York University Grossman School of Medicine, New York, NY, USA; c Department of Biostatistics, Gillings School of Global Public Health, University of North Carolina, Chapel Hill, NC, USA; d Department of Communication Sciences & Disorders, College of Behavioral & Community Sciences, University of South Florida, Tampa, FL, USA; e Department of Epidemiology, Johns Hopkins Bloomberg School of Public Health, Baltimore, MD, USA; f Cochlear Center for Hearing and Public Health, Johns Hopkins Bloomberg School of Public Health, Baltimore, MD, USA; g Department of Otolaryngology-Head & Neck Surgery, Johns Hopkins School of Medicine, Baltimore, MD, USA; h School of Psychology, University of Leeds, UK; i National Institute of Neurological Disorders & Stroke Intramural Research Program, National Institutes of Health, Bethesda, MD, USA; j Department of Internal Medicine, Wake Forest University School of Medicine, Winston-Salem, NC, USA; k Department of Neurology, Mayo Clinic, Rochester, MN, USA; l The MIND Center, University of Mississippi Medical Center, Jackson, MS, USA; m Department of Neurology, University of North Carolina at Chapel Hill School of Medicine, Chapel Hill, NC, USA; n Division of Epidemiology and Community Health, University of Minnesota School of Public Health, Minneapolis, MN, USA; o Welch Center for Prevention, Epidemiology and Clinical Research, Johns Hopkins University, Baltimore, MD, USA; p Department of Otolaryngology-Head & Neck Surgery, Morsani College of Medicine, University of South Florida, Tampa, FL, USA; q Department of Public Health Sciences, Wake Forest University School of Medicine, Winston-Salem, NC, USA

**Keywords:** Biomarkers, Neurology, Cognitive decline, Dementia, Hearing, Intervention

## Abstract

**Background:**

Research suggests that hearing loss in older adults is associated with elevated neuroinflammation and neurodegeneration, both of which are risk factors for dementia. In a secondary analysis of the Aging and Cognitive Health Evaluation in Elders (ACHIEVE) randomised controlled trial (ClinicalTrials.gov identifier: NCT03243422), we examined if hearing intervention slowed 3-year change in blood-based biomarkers of neuroinflammation (glial fibrillary acidic protein [GFAP]) and neurodegeneration (neurofilament light chain [NfL]).

**Methods:**

ACHIEVE, a clinical trial in the United States conducted between January 4, 2018 and November 30, 2022, tested the effects of hearing intervention (audiological counselling, provision of hearing aids) versus health education control (counselling on chronic disease prevention) on 3-year cognitive decline among older adults without substantial cognitive impairment and with untreated hearing loss. Plasma was collected at baseline and three years later among ACHIEVE participants recruited from the Atherosclerosis Risk in Communities cohort. Covariate-adjusted linear mixed effects models estimated intention-to-treat effects on 3-year change in inverse-normal transformed values of GFAP and NfL.

**Findings:**

Participants in the analytic sample (n = 164) were mean (SD) age 78.1 (2.9) years, 64.0% female, and 73.2% White. Over three years, hearing intervention was associated with a slower rise in GFAP (intervention: −0.026; 95% CI: −0.144, 0.093, control: 0.149; 95% CI: 0.039, 0.260; difference: −0.175; 95% CI: −0.322, −0.028; p-difference: 0.019) and NfL (intervention: 0.171; 95% CI: 0.022, 0.319, control: 0.330; 95 %CI: 0.185, 0.475, difference: −0.159; 95 %CI: −0.348, 0.029; p-difference: 0.098).

**Interpretation:**

Randomisation to hearing intervention was associated with a slower three-year rise in a blood-based biomarker of neuroinflammation. Comparable estimates were observed for neurodegeneration, although the effect was not statistically significant. Findings suggest that hearing intervention may reduce the rate of change in non-specific blood-based biomarkers associated with brain health and dementia progression.

**Funding:**

US National Institutes of Health.

**Research in context**

**Evidence before this study**

We searched PubMed on September 19, 2025 using the search terms “(randomised trial) AND (hearing) AND (biomarker)” and further restricted retrieved studies to those that included a study population of adults without prevalent cognitive impairment or dementia, tested an intervention involving technologies or strategies for hearing loss treatment, had trial follow-up of >1 year, and examined changes in blood-based biomarkers of neuroinflammation and neurodegeneration. No published trials or secondary analyses of existing trials were identified that met these criteria.

**Added value of this study**

To the investigators’ knowledge, this is the first study to examine the effect of hearing intervention on longitudinal change in blood-based biomarkers of neuroinflammation (GFAP) and neurodegeneration (NfL) in older adults with hearing loss. In a secondary analysis of the ACHIEVE randomised controlled trial, hearing intervention was associated with slower 3-year change in GFAP. A similar but not statistically significant trend was observed for NfL.

**Implications of all the available evidence**

Findings suggest randomisation to hearing intervention in older adults may reduce the risk of dementia by slowing the progression of non-specific blood-based biomarkers of brain health. Hearing loss is a highly prevalent, modifiable target with potential for dementia prevention; established interventions confer no medical risk and their range of benefits may extend to preserving brain health.

## Introduction

Among the known potentially modifiable risk factors for dementia, consensus reports have identified hearing loss as a prominent target.^1^ Prior research suggests the association between hearing loss and dementia may be attributable to reduced auditory stimulation causing structural and functional changes in the brain.^2^ Emerging research^3^, ^4^, ^5^ also shows associations between hearing loss and non-specific biomarkers of neuroinflammation and neurodegeneration that are associated with dementia progression.^6^

Blood-based biomarkers of neuroinflammation and neurodegeneration are clinically important for monitoring progression towards all-cause dementia.^7^ Glial Fibrillary Acidic Protein (GFAP) is a protein that helps maintain the structural integrity of astrocytes and can serve as an indicator of neuroinflammation.^7^ Neurofilament Light Chain (NfL) is a neuronal protein routinely released into extracellular space that can be used to quantify neurodegeneration.^7^ Elevated concentrations of NfL and GFAP have been observed during the preclinical phases of several neurodegenerative disorders, including Amyotrophic Lateral Sclerosis, Parkinson’s disease, and dementias including frontotemporal dementia and Alzheimer’s disease (AD).^8^ Change in these biomarkers can function as objective, non-specific indicators of dementia risk.^9^

Hearing intervention may slow cognitive decline in older adults, but clinical trial evidence is heterogenous. The primary results of the landmark ACHIEVE randomised controlled trial^10^ showed overall null effects of hearing intervention (audiological counselling and provision of hearing aids) versus health education control (individual sessions with a health educator covering topics on chronic disease prevention) on 3-year cognitive decline. However, a prespecified sensitivity analysis revealed that hearing intervention slowed cognitive decline by 48% among a subgroup of participants recruited from the Atherosclerosis Risk in Communities (ARIC) cohort^11^ who had greater risk for cognitive decline and incident dementia (e.g., older age, worse cognitive function at baseline, etc.).

In a secondary analysis of the ACHIEVE trial, we investigated the effects of hearing intervention versus health education control on change in GFAP and NfL in the subgroup of ARIC participants. This subgroup was selected because they are more representative of the general population of older adults with hearing loss and elevated dementia risk. By investigating the effect of hearing intervention versus health education control on objective indicators of dementia risk, this study provides an important and novel contribution to the literature.

## Methods

### Study design and participants

ACHIEVE is a multicentre, parallel-group, unblinded randomised controlled trial that investigated the effects of a best-practice hearing intervention versus a health education control on 3-year change in cognitive function among community-dwelling older adults (ClinicalTrials.gov identifier: NCT03243422, prospectively registered on July 7th, 2017).^10^^,^^12^ The trial was partially embedded within the scientific and physical infrastructure of the ARIC cohort,^12^ a prospective longitudinal study of adults initially recruited between 1987 and 1989 from four locations in the United States: Forsyth County, North Carolina; Jackson, Mississippi; suburbs of Minneapolis, Minnesota; Washington County, Maryland. At each site, ACHIEVE participants were recruited from two populations: 1) older adults co-enrolled in the ARIC cohort and 2) healthy de novo community volunteers from the surrounding community. Recruitment occurred between November 9, 2017 and October 25, 2019. Randomization occurred between January 4, 2018 and October 25, 2019. Each participant was followed for 3 years (eFig. S1) with their final assessment occurring between June 1, 2021 and November 30, 2022.

Recruitment methodologies and screening have been previously reported.^12^^,^^13^ The eligibility criteria were age (70–84 years), living situation (community-dwelling, not planning to move from the area), English language fluency, adult-onset bilateral hearing loss as defined by better-ear 4-frequency pure-tone average (PTA) at 0.5, 1, 2, and 4 kHz between 30 and 70 dB hearing level (dB HL), free from substantial cognitive impairment (mini-mental state examination score of ≥23 for participants with a high school degree or less and ≥25 for those with some college education or more), no difficulty with ≥2 or more activities of daily living (getting in/out of bed or chairs, bathing, dressing, eating, and toileting), visual acuity better than 20/63 on the MNREAD (University of Minnesota) acuity chart, no hearing aid use within the prior year, no concurrent enrolment in trials focused on auditory or cognitive exposures or outcomes, and a willingness to be randomised and participate in ACHIEVE interventions. Participants (Total: n = 977; ARIC: n = 238, de novo: n = 739) underwent 1:1 permuted block randomisation stratified by severity of hearing loss (PTA < 40 or ≥40 dB). Eligible spousal pairs or partners were randomly assigned as a unit and stratified by recruitment source and field site.^10^^,^^12^^,^^13^ All participants were offered the other intervention at the end of the 3-year period.

Funding sources for this investigation had no role in study design, data collection, data analysis, data interpretation, or writing of this report. Prior to implementation, ethics approval for all procedures and protocols was obtained from the institutional review boards (single IRB# IRB00008129) at each participating field site and academic centre. Each participant provided written informed consent. The trial was conducted in accordance with the principles of the Declaration of Helsinki and Good Clinical Practice guidelines. An independent data and safety monitoring board met every six months to verify participant safety and review study progress.^10^^,^^12^^,^^13^ Adverse events of otitis externa, cerumen impaction or ear foreign body requiring removal by a physician, and death from any cause were monitored systematically throughout the trial. There were no adverse events that were unexpected and judged to be related to trial participation.

### Interventions

The hearing intervention was developed based on evidence-based best-practices for hearing care.^10^ Participants randomised to the hearing intervention completed four 1-h sessions with an audiologist every 1–3 weeks post-randomisation. Sessions included fitting and verifying hearing aids to prescriptive targets using real-ear measurements, provision of hearing assistive technologies to enhance hearing aid use in specific situations, extensive orientation and instruction on device use, communication strategies training, and counselling on self-efficacy and expectation management. Reinstruction was given during booster sessions held every six months post-randomisation.

The health education control comprised individual sessions with a certified health educator who administered the evidence-based 10 Keys to Healthy Aging™ program^14^ which focused on topics relevant to chronic condition management and disability prevention among older adults. Sessions were tailored to the participant and included goal setting, educational counselling, activities, and a 5- to 10-min upper body stretching program. To parallel the staff contact of the hearing intervention, participants completed four 1-h sessions every 1–3 weeks post-randomisation and returned for 6-month booster sessions.

### Outcomes

#### Blood-based biomarkers

**Fig. 1:**
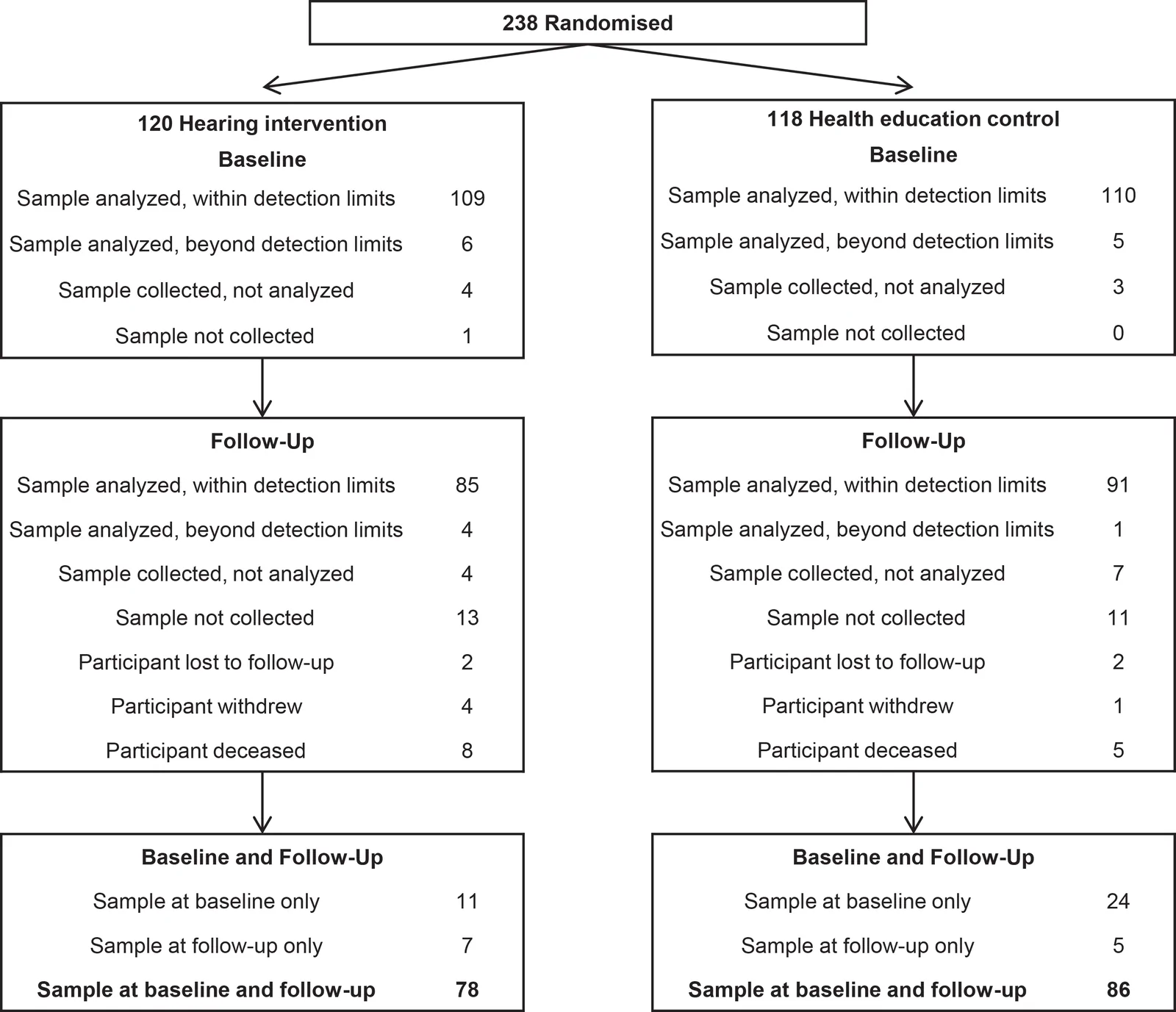
Analysis of blood-based biomarkers obtained at baseline and follow-up from ARIC participants enrolled in ACHIEVE stratified by randomisation. Abbreviations: ACHIEVE, Aging and Cognitive Health Evaluation in Elders Trial; ARIC, Atherosclerosis in Communities Cohort.

Ethylenediaminetetraacetic acid blood samples were collected from 237 ARIC cohort participants (Fig. 1) between June 29, 2016 and May 7, 2019. The blood draw occurred a median (IQI) of 10.4 (1.9, 14.6) months before randomisation for the ACHIEVE trial. Follow-up blood samples were obtained from 192 ARIC participants between June 1, 2021 and July 11, 2024. The median (IQI) time between randomisation and the second blood draw was 3.3 (3.0, 3.4) years. Among 403 ACHIEVE de novo participants, a blood draw was performed between January 17, 2023 and October 10, 2024 at a median (IQI) of 4.5 (4.2, 4.7) years after randomisation (eFig. S2).

Stored plasma samples were analysed (eMethods: Blood-Based Biomarkers) by the Advanced Research and Diagnostic Laboratory at the University of Minnesota between December 16, 2024 and December 21, 2024 using the Alamar Biosciences NUcleic acid Linked Immuno-Sandwich Assay (NULISA™) central nervous system (CNS) panel 120 kit (Alamar Part# 800104, Lot# 2407305). Prior to analysis, thawed samples were centrifuged for 5 min at 10,000×*g* and 4 °C. The samples were transferred to eleven 96-well microplates provided with the reagent kit and analysed in two batches. The plates were sealed and spun for 1 min at 1000×*g* and 4 °C. Utilising the automated Alamar Argo HT workflow, target analytes were bound through multiple capture, wash, and release steps, resulting in immunocomplexes with both sample-specific and target-specific DNA tags that were pooled into a single library for next generation sequencing. The library was sequenced using a P2 100 cycle flow cell (Illumina Part# 20046811, Reagent Lot# 20841078, Flow cell Lot# 20846922) on a NexSeq1000. After sequencing, the FASTQ file was uploaded to Argo Command Centre for automatic normalisation using internal and inter-plate controls and then log2-transformed for conversion to NULISA protein quantification (NPQ) units.^15^ Higher NPQ scores indicate greater concentrations of a biomarker. A 1-unit change corresponds to a 2-fold increase in the concentration of the biomarker. A 2-unit change corresponds to a 4-fold increase. Concentrations are biomarker-specific and cannot be compared to each other.

The limit of detection was defined by the manufacturer as three times the standard deviation of the normalised counts of plate-specific negative controls. Sufficient plasma for laboratory analysis was available from 230 ARIC participants who provided blood prior to randomisation (Fig. 1). A total of 219 samples (95.2% [219/230]) were within limits of detection. At the follow-up, 181 ARIC participants had sufficient plasma for analysis and 176 samples (97.2% [176/181]) produced measurements within the limits of detection. Among de novo participants (eFig. S2), 389 samples from the follow-up were analysed which resulted in 376 measurements (96.7% [376/389]) within the limits of detection.

Reproducibility was examined by analysing plasma obtained on the same day from the same ARIC participant but placed in two separate vials. Sixteen paired vials were analysed using a single lot across five plates examined as a single batch. The range of the reproducibility sample (N = 16, GFAP: 11.9–14.6 NPQ, NfL: 11.9–13.3 NPQ, phosphorylated tau (pTau) at threonine 181 [pTau181]: 12.6–14.3 NPQ, pTau217: 11.0–12.6 NPQ, pTau231: 12.7–14.5 NPQ) was similar but attenuated when compared to the range of the primary analytic sample (N = 164, GFAP: 11.1–15.0 NPQ, NfL: 10.5–15.3 NPQ, pTau181: 12.0–15.5 NPQ, pTau217: 10.1–13.7 NPQ, pTau231: 12.2–16.0 NPQ). The intraclass correlation coefficient (ICC) of the reproducibility sample was calculated by dividing between-participant variance by the total variance and 95% confidence intervals (95% CI) were generated using percentile bootstrapping with 1000 samples. The ICC was excellent for GFAP (β: 0.97, 95% CI: 0.91, 0.99), pTau181 (β: 0.92, 95% CI: 0.85, 0.98), and pTau231 (β: 0.95, 95% CI: 0.91, 0.99). The ICC was acceptable for NfL (β: 0.80, 95% CI: 0.64, 0.94) and pTau217 (β: 0.88, 95% CI: 0.79, 0.97). The CV was calculated as the average of each within-participant ratio of the standard deviation to the mean. The CV was excellent for GFAP (0.6%), NfL (0.4%), pTau181 (0.6%), pTau217 (1.0%), and pTau231 (0.6%). Within-participant error was quantified as the standard error of measurement, which was subsequently used to calculate minimal detectable change with 95% confidence. Minimal detectable change was 1.55 (95% CI: 0.85, 1.89) for GFAP, 1.01 (95% CI: 0.75, 1.17) for NfL, 1.12 (95% CI: 0.74, 1.34) for pTau181, 1.33 (95% CI: 0.93, 1.52) for pTau217, and 1.28 (95% CI: 0.91, 1.51) for pTau231. To account for measurement error and biological variation, reference change values (RCVs) were computed for GFAP (6.6%), NfL (11.2%), pTau181 (7.8%), pTau217 (6.8%), and pTau231 (7.6%).

Although the NULISA™panel measured 120 biomarkers including pTau181, pTau217, and pTau231, prior research^4^^,^^16^ suggests hearing loss among older adults exhibits stronger associations with non-specific biomarkers of neuroinflammation (GFAP) and neurodegeneration (NfL) than biomarkers specific to AD pathology (pTau). Consequently, the primary analysis was restricted to the pre-specified primary outcomes of GFAP and NfL. In exploratory analyses, associations with pTau181, pTau217, and pTau231 were examined to provide a more comprehensive depiction of the effects of hearing intervention on neuropathology.

#### Cognition

Although not the focus of this investigation, the effect of hearing intervention on cognition was examined as a secondary outcome to evaluate whether previously reported protective effects^10^ persisted among ARIC participants with repeated biomarker measurements. Cognition was assessed by a 10-test cognitive battery (eMethods: Cognitive Battery). The battery included the Digit Span Backwards, Boston Naming Test, Word Fluency Test, Animal Naming Score, Digit Symbol Substitution, Trail Making Tests A and B, Incidental Learning, Logical Memory Test, and the Delayed Word Recall. Scores from the cognitive battery were used to compute a factor score of cognition.^17^ To reduce confounding, tests with exclusively auditory stimuli (Digit Span Backwards, Logical Memory Test) were not included in the model that generated factor scores for this investigation. Factor scores were standardised to the baseline of ACHIEVE.

### Covariates

Date of birth, sex, race, education (less than high school education, high school education or equivalent, some college education or more), and annual income (<$5,000, $5000 to $7,999, $8000 to $11,999, $12,000 to $15,999, $16,000 to $24,999, $25,000 to $34,999, $35,000 to $49,999, $50,000 to $74,999, $75,000 to $99,999, >$100,000) were self-reported at the baseline. Date of birth was used to calculate age. The field site each participant was recruited by, the recruitment source (ARIC or de novo), and whether the participant enrolled with a spouse or partner was documented.

Genotyping of apolipoprotein E (APOE) polymorphism at codons 112 (rs429358) and 158 (rs7412) in exon 3 was performed using a custom designed TaqMan allele discrimination assay (Applied Biosystems; Foster City, California). A 96-bp product for assay rs429358 was amplified utilising 0.9 μM each of the forward primer 5’-GGGCACGGCTGTCCAA-3’ and the reverse primer 5’-CCTCGCCGCGGTACTG-3’, 0.2 μM of each of the sequence-specific probes 5’-6FAM-CGGCCGCACACGTCCTCC-TAMRA-3’ and 5’-VIC-CGGCCGCGCACGTCCT-TAMRA-3’, 50 ng DNA, 5.0 mM MgCl_2_, and 1X TaqMan Universal PCR Master Mix containing AmpliTaq Gold DNA Polymerase in a 22 μl single–plex reaction. A 157-bp product for assay rs7412 was amplified utilising 0.9 μM each of the forward primer 5’-TCCCACCTGCGCAAGCT-3’ and the reverse primer 5’-GCACGCGGCCCTGTT-3’, 0.2 μM of each of the sequence-specific probes 5’-6FAM-CCTGCAGAAGCGCCTGGCA-TAMRA-3’ and 5’-VIC-GACCTGCAGAAGTGCCTGGCAGT-TAMRA-3’, 50 ng DNA, 5.0 mM MgCl_2_, and 1X TaqMan Universal PCR Master Mix containing AmpliTaq Gold DNA Polymerase in a 22 μl single–plex reaction. Each PCR assay included an initial step of 2 min at 50 °C and 10 min at 95 °C to activate the AmpliTaq Gold, and the products were amplified using 40 cycles of 15 s at 95 °C and 1 min at 60 °C on a DNA Engine Tetrad Thermal Cycler (MJ Research; Waltham, MA). End point allele detection and genotype calling were performed using the ABI 7700 and Sequence Detection System software (Applied Biosystems; Foster City, California).

Objective hearing was quantified through audiometry performed in sound attenuating rooms. Pure-tone air- and bone-conduction thresholds were assessed in each ear using a modified psychophysical bracketing method.^18^ Body weight was measured to the nearest 0.1 kg, and height was measured to the nearest centimetre. Body mass index (BMI) was calculated as weight in kilograms divided by height in metres squared. Kidney disease was defined as self-reported physician diagnosis. Details about additional measures obtained at the baseline of ACHIEVE are provided in the Supplemental materials (eMethods: Additional Baseline Measures).

### Statistical analyses

All analyses were conducted in SAS (version 9.4). MICE was used to impute missing covariates. The imputation model is described in the Supplemental materials (eMethods: Multiple Imputation).

To prevent outliers from biasing results, a rank-based inverse normal transformation was applied to biomarker concentrations. Scatterplots of change in transformed and untransformed biomarkers stratified by randomisation were generated. Descriptive characteristics were compared by randomisation, recruitment source (ARIC or de novo), and inclusion in the primary analytic sample (N = 164).

The intention-to-treat effect of randomised treatment assignment (hearing intervention versus health education control) on 3-year change in the pre-specified primary outcomes of GFAP and NfL was estimated among ARIC participants with baseline and follow-up blood samples (N = 164). A two-level linear mixed effects model with a random intercept, a random time slope, and an unstructured covariance matrix was specified since this model was used in previous analyses^10^ and exhibited comparable or better fit to the data than simpler models, such as a model with a compound symmetry covariance matrix (Bayesian Information Criterion [BIC]: 788.5 versus 847.0) or a model without a random time slope (BIC: 788.5 versus 788.0). Time in years between the baseline (first blood draw) and follow-up (second blood draw) was defined as the timescale. Point estimates and 95% CIs were generated using restricted maximum likelihood with a Kenward-Roger correction. Estimates from the analysis of imputed datasets were combined according to Rubin’s rules. The initial model generated crude, unadjusted estimates of change over time. The primary model adjusted for baseline measures of age, sex, field site, education, hearing loss, and the presence of APOE ε4 alleles. An interaction was specified between each covariate and time. A third model mitigated the influence of dilution^19^ and inadequate filtration^20^ by adjusting for BMI and kidney disease and including an interaction between these covariates and time. To facilitate comparisons with previously reported results from ACHIEVE,^10^ the analyses were repeated for the secondary outcome of cognition. Estimates of the effect of hearing intervention do not account for multiple comparisons and two-sided p-values are provided for descriptive purposes only.

Sensitivity analyses were conducted to evaluate the robustness of the results. The first analysis used complete case data to estimate the intention-to-treat effect. The second analysis examined change in untransformed biomarkers using complete case or imputed data. The third analysis examined change in both GFAP and NfL by specifying a multivariate three-level mixed effects model that included a random intercept and time slope for each participant (level 3) and a random intercept for the two biomarkers (level 2). The fourth analysis used a two-level mixed effects model fit to imputed data to examine the effect of intervention compliance by generating per-protocol estimates among participants who completed the assigned intervention, had no major protocol deviations, never wore hearing aids if they were assigned to the control, and never discontinued hearing aid use if they were assigned to the hearing intervention. The fifth analysis estimated the complier average causal effect using inverse probability weighting. The sixth analysis mitigated selection bias by incorporating inverse probability of selection weights into intention-to-treat models (eMethods: Inverse Probability Weighting).

In a supplemental prospective analysis, the intention-to-treat effect of randomised treatment assignment on differences in the pre-specified primary outcomes (GFAP, NfL) and the secondary outcome (cognition) at the three-year follow-up was estimated in all participants (ARIC and de novo) with follow-up blood samples (n = 552, eFig. S1) using a linear regression model. The initial model adjusted for the time in years between randomisation and when the follow-up blood sample was obtained. The primary model added baseline measures of age, sex, recruitment source, field site, education, hearing loss, and the presence of APOE ε4 alleles. To mitigate the influence of dilution and inadequate filtration, a third model integrated baseline measures of BMI and kidney disease. The prospective analysis was repeated separating ARIC participants (n = 176) and de novo participants (n = 376). The interaction between recruitment source and randomisation was estimated among all participants (n = 552).

In an exploratory analysis designed to provide a more comprehensive evaluation of the effects of hearing intervention on neuropathology, biomarkers of AD pathology (pTau181, pTau217, and pTau231) were examined. The initial analysis used imputed and complete case data to replicate the models that estimated the effect of randomised treatment assignment on change in transformed and untransformed exploratory biomarkers. A subsequent analysis replicated the prospective models that examined the effect of randomisation on differences at the three-year follow-up.

### Role of the funding source

Funding sources for this investigation had no role in study design, data collection, data analysis, data interpretation, or writing of this report.

## Results

In the primary analytic sample (Fig. 1) of 164 participants (Table 1), the mean (SD) age was 78.1 (2.9) years, 64.0% (105/164) were women, 26.8% (44/164) were Black, 73.2% (120/164) were White, and the mean (SD) better-ear 4-frequency PTA was 38.94 (6.61) dB. Compared to the 977 participants in the ACHIEVE trial (eTable S1), participants examined in this investigation were more likely to be Black (26.8% [44/164] versus 11.5% [112/977]), less likely to have a college education (48.2% [79/164] versus 53.4% [521/976]), and more likely to earn less than 25,000 US$ per year (24.2% [38/157] versus 15.5% [147/950]). There were minimal differences when compared to participants in the ACHIEVE trial recruited from the ARIC cohort (eTable S1). The baseline differences between participants randomised to the hearing intervention versus health education control were also minimal (Table 1, eTable S2).

**Table 1:** Demographic and clinical characteristics of ACHIEVE participants with baseline and follow-up blood-based biomarkers stratified by randomly assigned treatment (n = 164).

	All	Intervention (n = 78)	Control (n = 86)	p
Age, years (n = 164)	78.1 (2.9)	78.3 (3.0)	77.9 (2.8)	0.31
Female sex (n = 164)	105 (64.0)	49 (62.8)	56 (65.1)	0.76
Black race (n = 164)	44 (26.8)	20 (25.6)	24 (27.9)	0.74
White race (n = 164)	120 (73.2)	58 (74.4)	62 (72.1)	0.74
Field site (n = 164)				
Forsyth County, North Carolina	38 (23.2)	17 (21.8)	21 (24.4)	0.32
Jackson, Mississippi	43 (26.2)	19 (24.4)	24 (27.9)	
Minneapolis, Minnesota	30 (18.3)	13 (16.7)	17 (19.8)	
Washington County, Maryland	53 (32.3)	29 (37.2)	24 (27.9)	
Education (n = 164)				
Less than high school	17 (10.4)	9 (11.5)	8 (9.3)	0.73
High school, GED, or vocational school	68 (41.5)	32 (41.0)	36 (41.9)	
Some college, graduate, or professional school	79 (48.2)	37 (47.4)	42 (48.8)	
Income, US$ (n = 157)				
Under $25,000	38 (24.2)	18 (24.0)	20 (24.4)	0.049
$25,000–$49,999	54 (34.4)	34 (45.3)	20 (24.4)	
$50,000–$74,999	35 (22.3)	14 (18.7)	21 (25.6)	
$75,000–$100,000	13 (8.3)	3 (4.0)	10 (12.2)	
Over $100,000	17 (10.8)	6 (8.0)	11 (13.4)	
Enrolled as spousal pair (n = 164)	16 (9.8)	5 (6.4)	11 (12.8)	0.17
One or more apolipoprotein E ε4 alleles (n = 160)	41 (25.6)	16 (21.6)	25 (29.1)	0.28
Body mass index, kg/m^2^ (n = 163)	29.1 (5.4)	29.6 (4.6)	28.6 (6.0)	0.24
Diabetes (n = 164)	45 (27.4)	21 (26.9)	24 (27.9)	0.89
Systolic blood pressure, mm HG (n = 160)	128.5 (15.5)	125.4 (15.1)	131.5 (15.4)	0.013
Diastolic blood pressure, mm HG (n = 160)	65.5 (10.7)	64.7 (10.9)	66.3 (10.6)	0.36
Hypertension (n = 161)	111 (68.9)	52 (66.7)	59 (71.1)	0.54
Kidney disease (n = 164)	12 (7.3)	7 (9.0)	5 (5.8)	0.44
Alcohol use (n = 164)				
Current	81 (49.4)	36 (46.2)	45 (52.3)	0.99
Former	40 (24.4)	24 (30.8)	16 (18.6)	
Never	43 (26.2)	18 (23.1)	25 (29.1)	
Cigarette use (n = 164)				
Current	4 (2.4)	4 (5.1)	0 (0.0)	0.17
Former	64 (39.0)	31 (39.7)	33 (38.4)	
Never	96 (58.5)	43 (55.1)	53 (61.6)	
Pure tone average, dB (n = 164)	38.94 (6.61)	39.58 (7.05)	38.36 (6.16)	0.24
Pure tone average ≥ 40 db (n = 164)	68 (41.5)	34 (43.6)	34 (39.5)	0.60
Hearing handicap inventory score (n = 163)	12.26 (9.48)	12.86 (10.33)	11.72 (8.68)	0.45
Quick speech in noise (n = 164)	18.42 (5.51)	17.90 (5.85)	18.90 (5.18)	0.25
Mini-mental state examination score (n = 164)	28.18 (1.71)	28.29 (1.72)	28.09 (1.71)	0.46
Intervention drop-in (n = 86)	8 (9.3)		8 (9.3)	
Intervention drop-out (n = 78)	3 (3.8)	3 (3.8)		
Time between blood-based biomarkers, years (n = 164)	4.2 (0.8)	4.1 (0.8)	4.2 (0.9)	0.29
Blood-based biomarkers in NPQ units, mean (SD)				
Glial fibrillary acidic protein (n = 164)				
Baseline	13.14 (0.62)	13.06 (0.60)	13.21 (0.62)	0.10
Follow-up	13.21 (0.65)	13.05 (0.62)	13.35 (0.65)	0.0029
Three-year change	0.05 (0.31)	−0.01 (0.34)	0.10 (0.27)	0.029
Neurofilament light chain (n = 164)				
Baseline	12.49 (0.69)	12.48 (0.77)	12.50 (0.61)	0.84
Follow-up	12.74 (0.77)	12.63 (0.76)	12.83 (0.77)	0.092
Three-year change	0.17 (0.49)	0.09 (0.51)	0.25 (0.45)	0.035
Blood-based biomarkers in standard deviations, mean (SD)				
Glial fibrillary acidic protein (n = 164)				
Baseline	−0.03 (0.96)	−0.16 (0.93)	0.08 (0.98)	0.11
Follow-up	0.07 (1.00)	−0.16 (0.96)	0.29 (0.99)	0.0035
Three-year change	0.07 (0.48)	−0.01 (0.54)	0.15 (0.41)	0.039
Neurofilament light chain (n = 164)				
Baseline	0.04 (0.98)	0.00 (1.07)	0.07 (0.89)	0.65
Follow-up	0.37 (1.02)	0.23 (1.06)	0.51 (0.97)	0.079
Three-year change	0.24 (0.61)	0.14 (0.67)	0.33 (0.55)	0.055
Cognition, mean (SD) (n = 160)				
Baseline	−0.24 (1.01)	−0.23 (1.06)	−0.24 (0.96)	0.96
Follow-up	−0.59 (1.18)	−0.47 (1.25)	−0.69 (1.12)	0.24
Three-year change	−0.34 (0.51)	−0.25 (0.56)	−0.42 (0.44)	0.027

ACHIEVE, Aging and Cognitive Health Evaluation in Elders Trial; dB, decibels; GED, General educational development credential; kg, kilograms; m, metres; mm HG, millimetres of mercury; NULISA protein quantification; NPQ.

Data are n (%) or mean (SD). Denominators for percentages are based on the number of participants with complete data as indicated after each characteristic. Sex, race, education, income, cigarette use, and alcohol use were self-reported. The presence of apolipoprotein E ε4 alleles was ascertained by the TaqMan assay (Applied Biosystems, Foster City, California). Diabetes and kidney disease were defined as present if the participant reported using medication or self-reported a physician diagnosis. Sitting blood pressure was measured using a random zero sphygmomanometer. Hypertension was defined as present based on the use of antihypertensive medication, systolic blood pressure greater than or equal to 140 mm Hg, or diastolic blood pressure greater than or equal to 90 mm Hg. Glial fibrillary acidic protein and neurofilament light chain were measured using the Alamar NUcleic acid Linked Immuno-Sandwich Assay (NULISA™) central nervous system 120 kit, which produces sample-specific NPQ units. Higher scores indicate greater neuroinflammation or neurodegeneration. A 1-unit change corresponds to a 2-fold increase in the concentration of the biomarker. A 2-unit change corresponds to a 4-fold increase in the concentration. To prevent outliers from biasing results, a rank-based inverse normal transformation was applied to each biomarker prior to statistical analysis. A factor score quantifying cognition was developed using a validated latent variable modelling approach and standardised to the baseline, with lower scores indicating worse cognitive function. Univariate differences assessed using χ2 tests, t tests, and Cochran–Armitage trend tests.

Scatterplots depicting change in pre-specified primary (GFAP, NfL) and exploratory (pTau181, pTau217, and pTau231) biomarkers over time (eFigs. S3–S6) revealed that the progression rate was slightly slower in participants randomised to the hearing intervention—particularly among individuals with elevated biomarker concentrations at the baseline. This effect led to a statistically significant difference in the three-year rate of change for GFAP (Table 1), pTau181, and pTau231 (eTable S2). Only a few participants had increases over time that exceeded the RCVs for GFAP (intervention: 0 participants; control: 4 participants), NfL (intervention: 1 participant; control: 5 participants), pTau181 (intervention: 1 participant; control: 2 participants), pTau217 (intervention: 3 participants; control: 1 participant), and pTau231 (intervention: 1 participant; control: 2 participants).

**Fig. 2:**
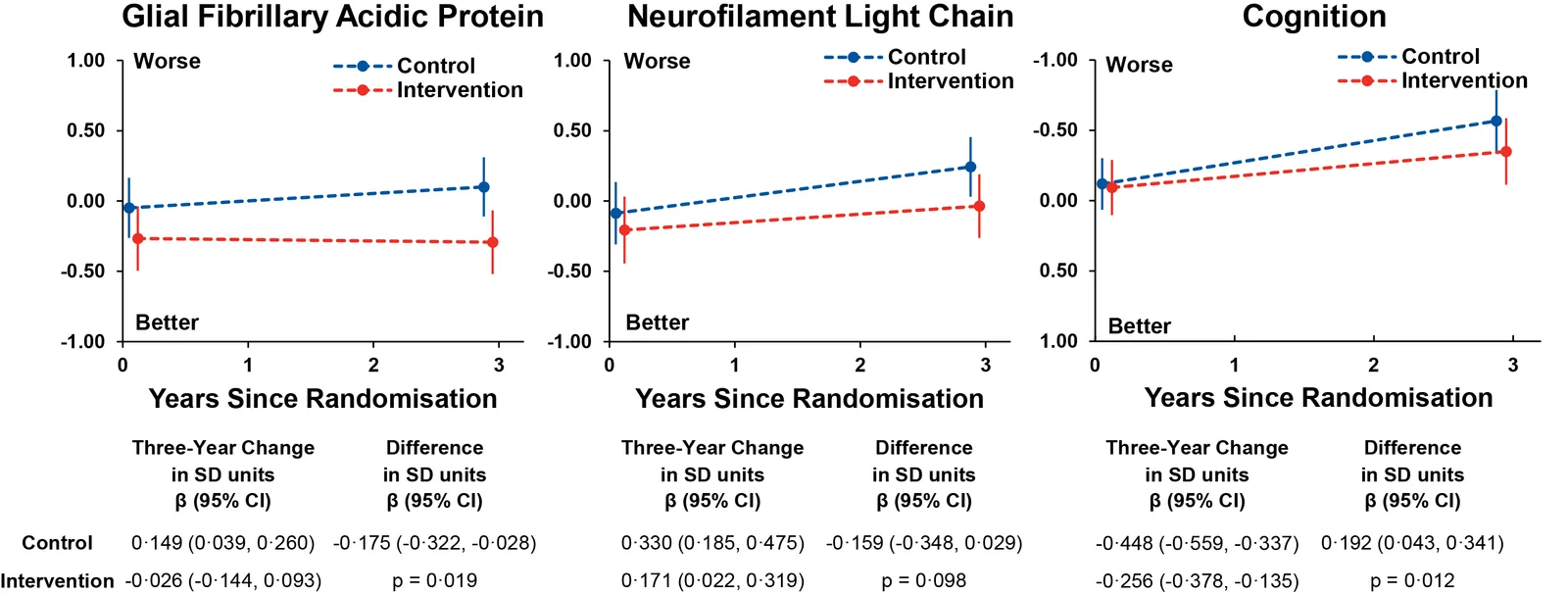
Covariate-adjusted intention-to-treat analysis of three-year change in pre-specified blood-based biomarkers and cognition among participants with baseline and follow-up blood-based samples in ACHIEVE by randomly assigned treatment (n = 164). Abbreviations: ACHIEVE, Aging and Cognitive Health Evaluation in Elders Trial; CI, confidence interval; SD, standard deviation. Point estimates, 95% confidence intervals, and p values were calculated by fitting separate linear mixed effects models for each outcome to imputed data. Each model specified time from baseline as the timescale, included a random intercept and time slope, employed an unstructured variance-covariance matrix, and adjusted for baseline measures of age, sex, field site, education, hearing loss (pure tone average <40 dB versus ≥40 dB), and the presence of apolipoprotein E ε4 alleles. An interaction was specified between each covariate and time. Glial fibrillary acidic protein and neurofilament light chain were measured using the Alamar NUcleic acid Linked Immuno-Sandwich Assay (NULISA™) central nervous system 120 kit. Higher scores indicate greater neuroinflammation or neurodegeneration. To prevent outliers from biasing results, a rank-based inverse normal transformation was applied to each biomarker prior to statistical analysis. A factor score quantifying cognition was developed using a validated latent variable modelling approach and standardised to the baseline, with lower scores indicating worse cognitive function.

Covariate-adjusted intention-to-treat analyses of three-year change (Fig. 2, eTable S3) in the primary analytic sample (Fig. 1) revealed that the hearing intervention was associated with a slower rise in GFAP (intervention: −0.026; 95% CI: −0.144, 0.093, control: 0.149; 95% CI: 0.039, 0.260; difference: −0.175; 95% CI: −0.322, −0.028; p-difference: 0.019). A comparable effect was observed for NfL (intervention: 0.171; 95% CI: 0.022, 0.319, control: 0.330; 95% CI: 0.185, 0.475, difference: −0.159; 95% CI: −0.348, 0.029; p-difference: 0.098), although the difference was not statistically significant. The hearing intervention also had a protective effect on the secondary outcome of cognitive decline (intervention: −0.256; 95% CI: −0.378, −0.135, control: −0.448; 95% CI: −0.559, −0.337, difference: 0.192; 95% CI: 0.043, 0.341; p-difference: 0.012) that replicated results from prior publications^10^ even though this investigation excluded tests (Digit Span Backwards, Logical Memory Test) with exclusively auditory stimuli. Comparable results were obtained when analysing factor scores that included all cognitive tests. A similar pattern of effects was observed in sensitivity analyses that examined complete cases (eTable S4), modelled untransformed biomarker concentrations (eTables S5 and S6), estimated the combined effect on GFAP and NFL (eTable S7), estimated the per-protocol effect (eTable S8), estimated the complier average causal effect (eTable S9), and accounted for selection bias (eTable S10). None of the statistically significant effects exceeded the RCV.

In an exploratory analysis of the primary analytic sample, hearing intervention was associated with a slower rise (eTable S11) in pTau181 (intervention: −0.022; 95% CI: −0.169, 0.125, control: 0.222; 95% CI: 0.085, 0.360; difference: −0.245; 95% CI: −0.427, −0.062; p-difference: 0.0087) and pTau231 (intervention: 0.056; 95% CI: −0.076, 0.188, control: 0.255; 95% CI: 0.132, 0.378; difference: −0.199; 95% CI: −0.363, −0.035; p-difference: 0.017). A similar effect was observed for pTau217 (intervention: 0.085; 95% CI: −0.034, 0.203, control: 0.201; 95% CI: 0.085, 0.316; difference: −0.116; 95% CI: −0.266, 0.034; p-difference: 0.13), but the difference between the intervention and control was not statistically significant. These effects persisted in sensitivity analyses (eTables S12–S15) but did not exceed the RCV.

**Fig. 3:**
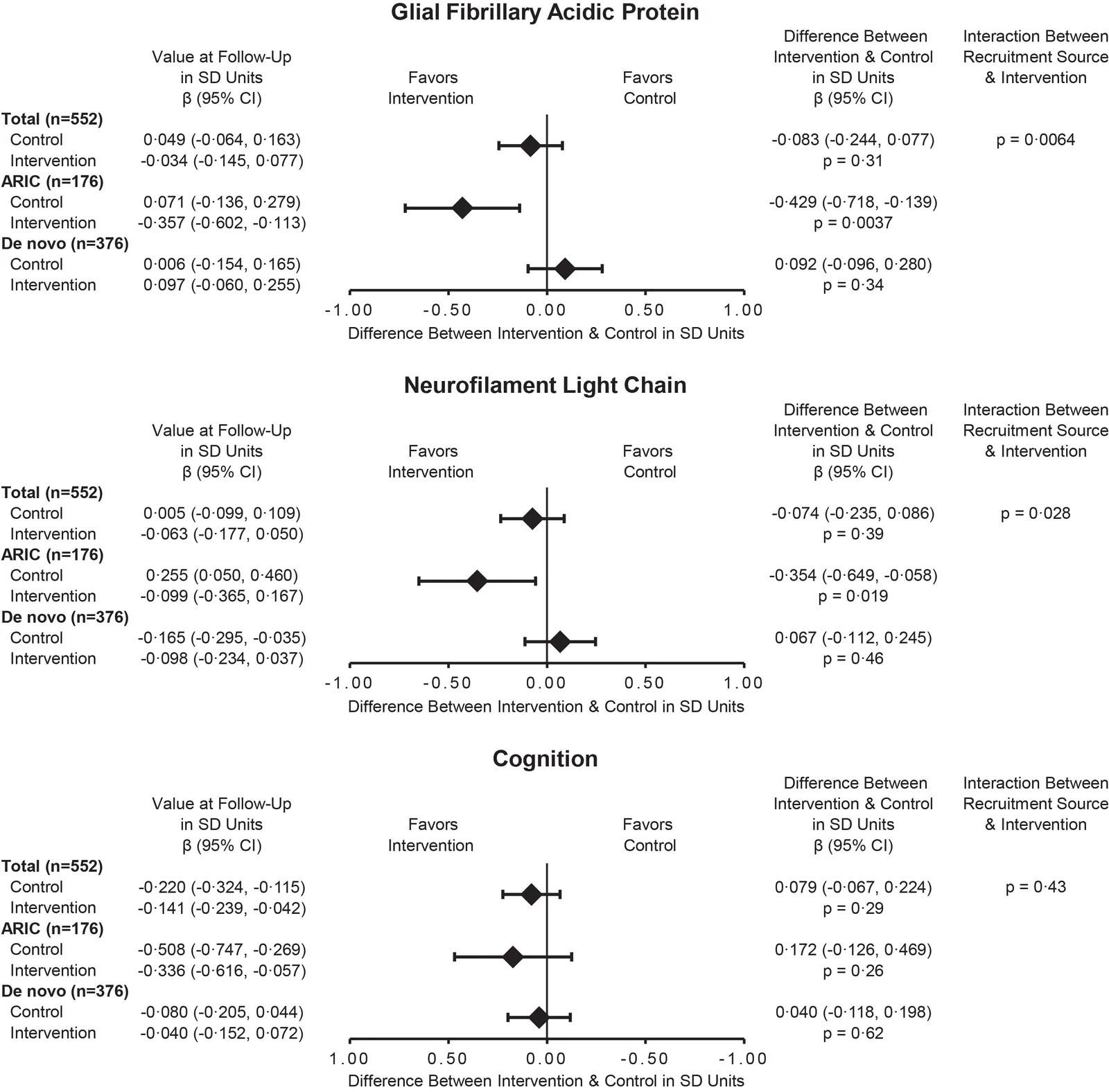
Supplemental covariate-adjusted intention-to-treat analysis of differences at the three-year follow-up in pre-specified blood-based biomarkers and cognition among participants with follow-up samples in ACHIEVE by randomly assigned treatment and recruitment source (n = 552). Abbreviations: ACHIEVE, Aging and Cognitive Health Evaluation in Elders Trial; ARIC, Atherosclerosis Risk in Communities Cohort; CI, confidence interval; SD, standard deviation. Point estimates, 95% confidence intervals, and p values were calculated by fitting separate linear regression models for each outcome to imputed data. The product of recruitment source and randomisation was added in a separate model to compute the p value for interaction. Each model adjusted for the time in years between randomisation and the follow-up assessment plus baseline measures of age, sex, recruitment source, field site, education, hearing loss (pure tone average <40 dB versus ≥40 dB), and the presence of apolipoprotein E ε4 alleles. Glial fibrillary acidic protein and neurofilament light chain were measured using the Alamar NUcleic acid Linked Immuno-Sandwich Assay (NULISA™) central nervous system 120 kit. Higher scores indicate greater neuroinflammation or neurodegeneration. To prevent outliers from biasing results, a rank-based inverse normal transformation was applied to each biomarker prior to statistical analysis. A factor score quantifying cognition was developed using a validated latent variable modelling approach and standardised to the baseline, with lower scores indicating worse cognitive function.

In a supplemental prospective analysis of all ACHIEVE participants (ARIC and de novo, eFig. S1) with follow-up blood samples (Total: n = 552; ARIC: n = 176, de novo: n = 376), biomarker concentrations were lower in the hearing intervention compared to the control at Year 3, but differences were not statistically significant (Fig. 3, eTable S16). A statistically significant interaction between recruitment source (ARIC, de novo) and treatment randomisation was detected for the pre-specified biomarkers of GFAP (p-interaction: 0.0064) and NfL (p-interaction: 0.028). In the de novo cohort, there was no effect of hearing intervention on GFAP and NfL at Year 3. However, among ARIC participants randomisation to hearing intervention was associated with lower (better) Year 3 levels of GFAP (intervention: −0.357; 95% CI: −0.602, −0.113, control: 0.071; 95% CI: −0.136, 0.279; difference: −0.429; 95% CI: −0.718, −0.139; p-difference: 0.0037) and NfL (intervention: −0.099; 95% CI: −0.365, 0.167, control: 0.255; 95% CI: 0.050, 0.460; difference: −0.354; 95% CI: −0.649, −0.058; p-difference: 0.019). A similar protective effect was observed in sensitivity analyses (eTables S12–S23). In an analysis of exploratory biomarkers, hearing intervention was associated with lower (better) Year 3 levels of pTau181, pTau217, and pTau231 among ARIC participants (eTable S24). These association remained consistent across sensitivity analyses (eTable S25–S28).

## Discussion

In a secondary analysis of the ACHIEVE trial, randomisation to hearing intervention was associated with slower progression of a pre-specified non-specific biomarker of neuroinflammation (GFAP) over three years in a subset of participants for whom the intervention also reduced the rate of cognitive decline.^10^ A comparable effect was observed for the pre-specified biomarker of neurodegeneration (NfL), although the difference between the intervention and the control was not statistically significant. Exploratory analyses indicated a similar effect on AD-specific biomarkers (pTau181, pTau231). Collectively, these findings suggest that hearing intervention may slow the progression of objective measures of neuropathology.

Prior research on the association of hearing loss with non-specific biomarkers of neuroinflammation and neurodegeneration is limited. A cross-sectional analysis of 373 participants (mean age: 66.4, 55% female) reported associations with NfL and GFAP.^5^ In a midlife, longitudinal analysis of the Beaver Dam Offspring Study (n = 1529, mean age: 48.2 years, 54% female), baseline hearing loss was associated with accelerated increases in NfL over 10 years (hearing loss: 4.0% per year, no hearing loss: 3.2% per year)^4^; GFAP was not investigated. One prior study investigated hearing aid use and NfL. In a cross-sectional analysis of 30 patients with age-related hearing loss (mean age: 64.0, female: 83%, hearing: 35–64 dB HL) and 20 sex-matched controls (mean age: 60.4, female: 90%), an unadjusted comparison showed higher mean NfL in patients with hearing loss (p < 0.0001). Among patients with hearing loss, mean NfL was lower after six months of hearing aid use (p < 0.0001).^3^

Evidence of the effect of hearing loss on AD-pathology is also scarce. Some cross-sectional^5^ and longitudinal^21^ studies have reported statistically significant associations. Other cross-sectional^22^ and longitudinal^4^ studies have reported null findings. One study^3^ found that hearing aid use was associated with a decrease in pTau181 after six months. While intriguing, these findings are restricted to observational studies susceptible to confounding or, in the case of the effect of hearing aids, the lack of a randomly assigned control group.

To our knowledge, this is the first randomised controlled trial to examine the effect of hearing intervention versus health education control on blood-based biomarkers. Since treatment was randomly assigned, it is unlikely that a shared upstream cause of cochlear and cerebral pathology is the primary mechanism. Several alternative mechanisms may explain why hearing intervention might mitigate dementia risk. One possibility is that loss of auditory stimulation leads to chronic glial activation^23^ which causes neuroinflammation. Neuroinflammation in turn mediates changes in neurodegeneration and AD pathology.^24^ This sequence aligns with experimental work^25^ indicating that reducing auditory input is by itself sufficient to activate astrocytes and microglia and to accelerate amyloid deposition and tau phosphorylation in mouse models of Alzheimer’s disease, suggesting that deafferentation can drive neuroinflammation rather than merely accompany it. This sequence may explain consistently observed associations between adult-onset hearing loss and biomarkers of dementia.^26^ An alternative possibility is that hearing intervention preserves social connections^27^ which reduces anxiety, depression, and other factors associated with elevated NfL and GFAP.^28^ However, null findings of the association between loneliness and blood-based biomarkers^29^ make this mechanism less plausible. Ultimately, additional studies and future investigations that use such techniques as causal mediation are needed to clarify the underlying mechanisms and determine whether the greater statistical significance for GFAP than NfL reflects biological differences or statistical variation.

The current findings should be interpreted in context of important limitations. Pre-randomisation blood samples were not collected in de novo participants. Consequently, longitudinal change in biomarkers was only examined among 164 participants with pre-randomisation blood samples obtained through the ARIC cohort. The results may therefore be specific to populations with greater risk for cognitive decline and incident dementia (e.g., older age, worse cognitive function, etc.).^10^ Future analyses will investigate longitudinal changes in blood-based biomarkers obtained post-randomization from de novo participants if that data becomes available. Biomarker concentrations in the ARIC cohort were only available at two time points, limiting the precision of trajectory estimates. The small sample size and limited number of follow-up assessments also reduced statistical power. This was addressed by restricting the primary analysis a priori to the pre-specified outcomes of GFAP and NfL rather than testing all biomarkers and correcting for multiple comparisons. However, this approach introduced some type 1 error inflation. It is also important to consider that while reductions in the rate of change of GFAP, pTau181, and pTau231 were all statistically significant, they did not exceed reference change values. However, reference change values describe the variability of repeated measurements within a single participant, whereas the primary analysis depicts change in all participants. A difference smaller than the reference change value may still constitute real change at the group level while remaining uninterpretable for an individual patient. Replication in larger samples with more frequent sampling is needed. Finally, key covariates, such as multilingualism, were not measured in ACHIEVE which may have introduced measurement bias in the secondary outcome of cognition.

Overall, this investigation suggests that hearing intervention may reduce the progression of non-specific biomarkers of neuroinflammation among older adults with hearing loss who have multiple risk factors for incident dementia. This finding bolsters the role of hearing loss as a modifiable target for dementia prevention, as elevated levels of non-specific biomarkers of neuroinflammation and neurodegeneration are associated with both accelerated cognitive decline^30^ and greater risk of dementia^9^ in older community-dwelling adults. The findings also underscore the role of blood-based biomarkers as valuable tools that should be used in future dementia prevention trials,^30^ particularly in providing biological information in designs and populations where intervention effects on incident dementia may take longer to observe.

## Contributors

Conceptualisation: Alison R Huang, Nicholas Reed, James Pike, Frank R. Lin, Josef Coresh, Adele M. Goman, Christine Mitchell.

Data curation: James Pike.

Formal analysis: James Pike, Alison R Huang, Nicholas Reed, Josef Coresh.

Funding acquisition: Frank R. Lin, Josef Coresh, Elizabeth Selvin.

Investigation: Bharat Thyagarajan, Michelle Arnold, Theresa Chisolm, Victoria Sanchez, Nicholas Reed, Adele M. Goman, Christine Mitchell, James Pankow, Thomas H. Mosley, Frank R. Lin.

Methodology: James Pike, Alison R Huang, Nicholas Reed, Josef Coresh.

Project administration: Josef Coresh, Alison R Huang, Frank R. Lin.

Writing—original draft: Alison R Huang, Nicholas Reed, James Pike.

Writing—review & editing: Michelle Arnold, Theresa Chisolm, David Couper, Jennifer A. Deal.

Rebecca F. Gottesman, Adele M. Goman, Timothy M. Hughes, David Knopman, Christine Mitchell.

Thomas H. Mosley, Priya Palta, James Pankow, Victoria Sanchez, Kevin J. Sullivan, Bharat.

Thyagarajan, Elizabeth Selvin, Lynne Wagenknecht, Frank R. Lin, Josef Coresh, ACHIEVE Collaborative Research Group.

All authors had access to the data, contributed to interpretation of the data, participated in writing and reviewing the manuscript, approved the final version for submission, and agreed to be accountable for the accuracy and integrity of the data.

## Data sharing statement

A deidentified dataset and data dictionary have been submitted to the National Heart, Lung, and Blood Institute (NHLBI) Biologic Specimen and Data Repository Information Coordinating Center (BioLINCC) and were made publicly available in 2026. Additional details on data access policies will be made available at https://www.achievestudy.org at that time. The study protocol and statistical analysis plan are available at https://www.clinicaltrials.gov.

## Declaration of interests

NSR reports employment by Amplifon USA. JAD reports participation in Cognition & Hearing Advisory Board and Sonova/Phonak Data Safety Monitoring Board with no financial renumeration. RFG reports being an unpaid member of the MarkVCID External Advisory Board. TMH reports funding from NIH grants: P30AG072947, R01AG054069, R01AG058969, RF1NS110043, U01HL096812, R01HL153191, R01AG070867, R01AG071032, R01AG072634, R01AG068629, R01HL158622, R01AG070881, R01AG067557, R21AG075291, R01AG080821, R01AG074971. DK reports participation in a data safety monitoring board in DIAN-TU from Washington University St. Louis as well as Roche Trontinemab. VAS reports grants from Otonomy Inc., Frequency Therapeutics Ltd., Pipeline Therapeutics, Aerin Medical, Oticon Medical, Helen of Troy Ltd., and Sensorion for Industry-sponsored clinical research contract to my institution to support research activity they conducted (not directly to them); a consulting fee from Autifony Therapeutics Ltd for paid consulting related to hearing and treatments for hearing loss that is not directly related to this manuscript; an honoraria related to presentations at a sponsored event from Oticon Medical, Sonova Holding AG, and Phonak USA; and serving on the COACH Trial Data Safety Monitoring Board. ES reports research grants and payments to their institution from the American Heart Association; royalty payments from Wolters Kluwer for chapters and laboratory monographs in UpToDate on measurements of glycemic control and screening tests for type 2 diabetes; payment from Siemens Healthineers for educational lecture; honorariums for a Deputy Editor role in Diabetes Care and Editorial Board role in Diabetologia and an unpaid leadership roles in the American Diabetes Association, International Federation of Clinical Chemistry, and International Diabetes Federation; and donated materials (reagents and devices) for NIH-supported research that includes Abbott Diabetes Care, Abbott Diagnostics, Asahi Kasei Pharma Corp, and Precision Diagnostics. FRL reports grants from the National Institutes of Health and Eleanor Schwartz Charitable Foundation; consulting fees from Apple Inc; paid fees for serving as an expert witness for the plaintiff in a class action lawsuit against an insurance company in Washington state pertaining to litigation relating to the insurance company’s policy of non-coverage for hearing aids; participation in the Scientific Advisory Boards for Fondation Pour L’Audition and Sharper Sense; volunteer board member of the nonprofit AccessHears; contributions from Sonova to Johns Hopkins University for hearing technologies used in the ACHIEVE trial; and functioning as the director of a public health research center at the Johns Hopkins Bloomberg School of Public Health funded in part by a philanthropic donation from Cochlear Ltd. JRP, ARH, MLA, THC, DC, SF, CM, THM, PP, JSP, KJS, BT, LEW, and JC report no conflicts of interest.

## Appendix A Supplementary data

Cover Letter

eMethods, eFigs. S1–S6, and eTables S1–S28

Protocol
