# Supplementary material for: Effects of hearing intervention on blood-based biomarkers of neuroinflammation and neurodegeneration: a secondary analysis of the ACHIEVE randomised controlled trial
Source: eClinicalMedicine. 2026 Sep 3;99:104164. doi: 10.1016/j.eclinm.2026.104164 (PMC13571546; doi:10.1016/j.eclinm.2026.104164)
Supplement: Supplementary file 2 [file mmc1.docx]

Monday, July 27, 2026

Dr. Claudia Schaefer
Editor in Chief
eClinicalMedicine

Dr. Schaefer:

Enclosed is a revised version of the manuscript entitled “Effects of hearing intervention on blood-based biomarkers of neuroinflammation and neurodegeneration: A secondary analysis of the ACHIEVE randomised controlled trial.” Revisions have been made based on comments from the reviewers. In addition, we have made changes to address the following issues.

**Throughout the paper, please clearly indicate each outcome reported as secondary, pre-specified, post-hoc or exploratory accordingly. The section Outcomes in the Methods needs to reflect this, as well as the Methods section of the Summary.**

Changes have been made through the manuscript and supplemental materials to clarify the nature of each outcome.

**Rename the "Abstract" as "Summary."**

Revised.

**Add Keywords immediately after the Summary.**

Completed.

**Move the Data Sharing statement to directly after the Contributors section.**

Revised.

**Separate figures from their legends: place the figure legends together after the References.**

Revised.

**Add an Ethics section within the Methods.**

Completed.

**Add a Role of Funders statement at the end of the Methods.**

Completed.

**Describe the methods in the main text rather than directing readers to the Supplementary Material (eMethods currently only in the supplement).**

We have moved many of the methodological details from the supplement to the main text. Some details remain in the supplement to stay within the journal’s recommended word count and reference limit. This material can also be moved if that is preferred.

**State the exact dates of recruitment and study conduct (add also to the Summary).**

Revised.

**State whether trial registration was prospective.**

Revised.

**Include the trial registration number in the Summary.**

Revised.

**Provide the ethical approval reference number(s) — currently only the trial registration number is given.**

The primary IRB number has been added. If there are additional reference numbers required, we would be happy to include them.

**Add a statement confirming adherence to the Declaration of Helsinki.**

Completed.

**In the Contributors section, explain how the consortium/study group listed in the byline contributed.**

Completed.

**Provide a declaration of interest for every author individually — some authors' interests are stated but others are missing (Page 10). If any authors have no conflicts, include the standard "no conflicts" statement for them (Page 10).**

Revised.

**Upload a completed CONSORT reporting checklist as a separate file — currently missing.**

Completed.

**Give absolute numbers alongside all percentages — currently inconsistent.**

The text of the manuscript has been updated to include absolute numbers for each percentage.

**Supply tables as separate editable Word files; they are currently embedded in the main manuscript (Page 14).**

Revised.

**Add the article title (and a contents list, if not present) to the Supplementary Material PDF — title is currently missing.**

Revised.

**Each author needs to complete the ICMJE form separately. Please note that all statements, on the ICMJE forms and in the Declaration of Interest statement in the manuscript, need to match EXACTLY.**

The Declaration of Interest statement has been updated and missing forms have been uploaded.

**All authors need to sign the Author signature form.**

Missing forms have been uploaded and forms without names and titles have been revised.

All co-authors have reviewed and approved the manuscript. The corresponding authors take full responsibility for the data, the analyses and interpretation, and the conduct of the research. No related papers from the same data have been published, posted, or submitted. We thank you for your consideration of this manuscript and look forward to your response.

James Russell Pike

Grossman School of Medicine

New York University

550 1st Avenue

New York, NY 10016

Email: [James.Pike@nyulangone.org](mailto:James.Pike@nyulangone.org)

Phone: 818-406-0286

Alison Huang

Cochlear Center for Hearing and Public Health

Department of Epidemiology

Johns Hopkins University

615 N. Wolfe St. Baltimore, MD 21205

Email: [ahuang31@jh.edu](mailto:ahuang31@jh.edu)

Phone: 717-580-9681

Josef Coresh, MD PhD

Grossman School of Medicine

New York University

550 1st Avenue

New York, NY 10016

Email: [Josef.Coresh@nyulangone.org](mailto:Josef.Coresh@nyulangone.org)
