# Supplementary material for: Effects of hearing intervention on blood-based biomarkers of neuroinflammation and neurodegeneration: a secondary analysis of the ACHIEVE randomised controlled trial
Source: eClinicalMedicine. 2026 Sep 3;99:104164. doi: 10.1016/j.eclinm.2026.104164 (PMC13571546; doi:10.1016/j.eclinm.2026.104164)
Supplement: Supplementary file 3 [file mmc2.docx]

**Effects of hearing intervention on blood-based biomarkers of neuroinflammation and neurodegeneration: A secondary analysis of the ACHIEVE randomised controlled trial**

**Supplemental Materials**

**Table of Contents**

[**eMethods**	4](#_Toc235339208)

[**Blood-Based Biomarkers**	4](#_Toc235339209)

[**Cognitive Battery**	4](#_Toc235339210)

[**Additional Baseline Measures**	5](#_Toc235339211)

[**Multiple Imputation**	5](#_Toc235339212)

[**Inverse Probability Weighting**	5](#_Toc235339213)

[**eTable 1.** Demographic and Clinical Characteristics of ACHIEVE Participants Stratified by Recruitment Source and Inclusion in Primary Analytic Sample (n=977)	7](#_Toc235339214)

[**eTable 2.** Demographic and Clinical Characteristics of ACHIEVE Participants with Follow-Up Blood-Based Biomarkers Stratified by Randomly Assigned Treatment (n=552)	8](#_Toc235339215)

[**eTable 3.** Intention-to-Treat Analysis of Three-Year Change in Pre-Specified Blood-Based Biomarkers and Cognition Among Participants with Baseline and Follow-Up Samples in ACHIEVE by Randomly Assigned Treatment (n=164)	10](#_Toc235339216)

[**eTable 4.** Complete Case Intention-to-Treat Analysis of Three-Year Change in Pre-Specified Blood-Based Biomarkers and Cognition Among Participants with Baseline and Follow-Up Samples in ACHIEVE by Randomly Assigned Treatment (n=164)	11](#_Toc235339217)

[**eTable 5.** Intention-to-Treat Analysis of Three-Year Change in Untransformed Pre-Specified Blood-Based Biomarkers Among Participants with Baseline and Follow-Up Samples in ACHIEVE by Randomly Assigned Treatment (n=164)	12](#_Toc235339218)

[**eTable 6.** Complete Case Intention-to-Treat Analysis of Three-Year Change in Untransformed Pre-Specified Blood-Based Biomarkers Among Participants with Baseline and Follow-Up Samples in ACHIEVE by Randomly Assigned Treatment (n=164)	13](#_Toc235339219)

[**eTable 7.** Multivariate Intention-to-Treat Analysis of Three-Year Change in Pre-Specified Blood-Based Biomarkers Among Participants with Baseline and Follow-Up Samples in ACHIEVE by Randomly Assigned Treatment (n=164)	14](#_Toc235339220)

[**eTable 8.** Per-Protocol Analysis of Three-Year Change in Pre-Specified Blood-Based Biomarkers and Cognition Among Participants with Baseline and Follow-Up Samples in ACHIEVE by Randomly Assigned Treatment (n=150)	15](#_Toc235339221)

[**eTable 9.** Complier Average Causal Effect Analysis of Three-Year Change in Pre-Specified Blood-Based Biomarkers and Cognition Among Participants with Baseline and Follow-Up Samples in ACHIEVE by Randomly Assigned Treatment (n=164)	16](#_Toc235339222)

[**eTable 10.** Selection Weighted Intention-to-Treat Analysis of Three-Year Change in Pre-Specified Blood-Based Biomarkers and Cognition Among Participants with Baseline and Follow-Up Samples in ACHIEVE by Randomly Assigned Treatment (n=164)	17](#_Toc235339223)

[**eTable 11.** Intention-to-Treat Analysis of Three-Year Change in Exploratory Blood-Based Biomarkers of Alzheimer's Disease Pathology Among Participants with Baseline and Follow-Up Samples in ACHIEVE by Randomly Assigned Treatment (n=164)	18](#_Toc235339224)

[**eTable 12.** Complete Case Intention-to-Treat Analysis of Three-Year Change in Exploratory Blood-Based Biomarkers of Alzheimer's Disease Pathology Among Participants with Baseline and Follow-Up Samples in ACHIEVE by Randomly Assigned Treatment (n=164)	19](#_Toc235339225)

[**eTable 13.** Intention-to-Treat Analysis of Three-Year Change in Untransformed Exploratory Blood-Based Biomarkers of Alzheimer's Disease Pathology Among Participants with Baseline and Follow-Up Samples in ACHIEVE by Randomly Assigned Treatment (n=164)	20](#_Toc235339226)

[**eTable 14.** Complete Case Intention-to-Treat Analysis of Three-Year Change in Untransformed Exploratory Blood-Based Biomarkers of Alzheimer's Disease Pathology Among Participants with Baseline and Follow-Up Samples in ACHIEVE by Randomly Assigned Treatment (n=164)	21](#_Toc235339227)

[**eTable 15.** Multivariate Intention-to-Treat Analysis of Three-Year Change in Exploratory Blood-Based Biomarkers of Alzheimer's Disease Pathology Among Participants with Baseline and Follow-Up Samples in ACHIEVE by Randomly Assigned Treatment (n=164)	22](#_Toc235339228)

[**eTable 16.** Intention-to-Treat Analysis of Differences in Pre-Specified Blood-Based Biomarkers and Cognition Among Participants with Follow-Up Samples in ACHIEVE by Randomly Assigned Treatment and Recruitment Source (n=552)	23](#_Toc235339229)

[**eTable 17.** Complete Case Intention-to-Treat Analysis of Differences in Pre-Specified Blood-Based Biomarkers and Cognition Among Participants with Follow-Up Samples in ACHIEVE by Randomly Assigned Treatment and Recruitment Source (n=552)	24](#_Toc235339230)

[**eTable 18.** Intention-to-Treat Analysis of Differences in Untransformed Pre-Specified Blood-Based Biomarkers Among Participants with Follow-Up Samples in ACHIEVE by Randomly Assigned Treatment and Recruitment Source (n=552)	25](#_Toc235339231)

[**eTable 19.** Complete Case Intention-to-Treat Analysis of Differences in Untransformed Pre-Specified Blood-Based Biomarkers Among Participants with Follow-Up Samples in ACHIEVE by Randomly Assigned Treatment and Recruitment Source (n=552)	26](#_Toc235339232)

[**eTable 20.** Multivariate Intention-to-Treat Analysis of Differences in Pre-Specified Blood-Based Biomarkers Among Participants with Follow-Up Samples in ACHIEVE by Randomly Assigned Treatment and Recruitment Source (n=552)	27](#_Toc235339233)

[**eTable 21.** Per-Protocol Analysis of Differences in Pre-Specified Blood-Based Biomarkers and Cognition Among Participants with Follow-Up Sample in ACHIEVE by Randomly Assigned Treatment and Recruitment Source (n=552)	28](#_Toc235339234)

[**eTable 22.** Complier Average Causal Effect Analysis of Differences in Pre-Specified Blood-Based Biomarkers and Cognition Among Participants with Follow-Up Samples in ACHIEVE by Randomly Assigned Treatment and Recruitment Source (n=552)	29](#_Toc235339235)

[**eTable 23.** Selection Weighted Intention-to-Treat Analysis of Differences in Pre-Specified Blood-Based Biomarkers and Cognition Among Participants with Follow-Up Samples in ACHIEVE by Randomly Assigned Treatment and Recruitment Source (n=552)	30](#_Toc235339236)

[**eTable 24.** Intention-to-Treat Analysis of Differences in Exploratory Blood-Based Biomarkers of Alzheimer's Disease Pathology Among Participants with Follow-Up Samples in ACHIEVE by Randomly Assigned Treatment and Recruitment Source (n=552)	31](#_Toc235339237)

[**eTable 25.** Complete Case Intention-to-Treat Analysis of Differences in Exploratory Blood-Based Biomarkers of Alzheimer's Disease Pathology Among Participants with Follow-Up Samples in ACHIEVE by Randomly Assigned Treatment and Recruitment Source (n=552)	32](#_Toc235339238)

[**eTable 26.** Intention-to-Treat Analysis of Differences in Untransformed Exploratory Blood-Based Biomarkers of Alzheimer's Disease Pathology Among Participants with Follow-Up Samples in ACHIEVE by Randomly Assigned Treatment and Recruitment Source (n=552)	33](#_Toc235339239)

[**eTable 27.** Complete Case Intention-to-Treat Analysis of Differences in Untransformed Exploratory Blood-Based Biomarkers of Alzheimer's Disease Pathology Among Participants with Follow-Up Samples in ACHIEVE by Randomly Assigned Treatment and Recruitment Source (n=552)	34](#_Toc235339240)

[**eTable 28.** Multivariate Intention-to-Treat Analysis of Differences in Exploratory Blood-Based Biomarkers of Alzheimer's Disease Pathology Among Participants with Follow-Up Samples in ACHIEVE by Randomly Assigned Treatment and Recruitment Source (n=552)	35](#_Toc235339241)

[**eFigure 1.** ACHIEVE Screening, Randomisation, Follow-Up, and Analysis of Blood-Based Biomarkers Stratified by Recruitment Source	36](#_Toc235339242)

[**eFigure 2.** Analysis of Blood-Based Biomarkers Obtained at Follow-Up from ACHIEVE Participants Stratified by Randomisation and Recruitment Source	37](#_Toc235339243)

[**eFigure 3.** Scatterplots of Three-Year Change in Pre-Specified Blood-Based Biomarkers Among Participants with Baseline and Follow-Up Samples in ACHIEVE by Randomly Assigned Treatment (n=164)	38](#_Toc235339244)

[**eFigure 4.** Scatterplots of Three-Year Change in Exploratory Blood-Based Biomarkers of Alzheimer's Disease Pathology Among Participants with Baseline and Follow-Up Samples in ACHIEVE by Randomly Assigned Treatment (n=164)	39](#_Toc235339245)

[**eFigure 5.** Scatterplots of Three-Year Change in Untransformed Pre-Specified Blood-Based Biomarkers Among Participants with Baseline and Follow-Up Samples in ACHIEVE by Randomly Assigned Treatment (n=164)	40](#_Toc235339246)

[**eFigure 6.** Scatterplots of Three-Year Change in Untransformed Exploratory Blood-Based Biomarkers of Alzheimer's Disease Pathology Among Participants with Baseline and Follow-Up Samples in ACHIEVE by Randomly Assigned Treatment (n=164)	41](#_Toc235339247)

[**Members of the ACHIEVE Collaborative Research Group**	42](#_Toc235339248)

[**References**	43](#_Toc235339249)

# **eMethods**

**Blood-Based Biomarkers**

Blood samples were initially placed in an ice bath until plasma was separated by centrifugation. The median (IQI) time between the blood draw and centrifugation was 5 (3, 8) minutes. Plasma was divided into aliquots in 1·5-mL tubes, frozen, and stored in -80°C freezers. These conditions have demonstrated stability for multiple biomarkers^1^ measured in the ARIC cohort^2^ even after more than a decade in storage.^3^ The median (IQI) time between when blood was frozen and when it was thawed at room temperature was 3·22 (2·71, 3·51) years. This timeframe aligns with prior studies^4^ that demonstrated the stability and accuracy of the Alamar Biosciences NUcleic acid Linked Immuno-Sandwich Assay (NULISA™) central nervous system 120 kit used in this investigation.

**Cognitive Battery**

The cognitive battery administered within the Aging and Cognitive Health Evaluation in Elders (ACHIEVE) trial included ten cognitive tests. All tests had visual stimuli with the exception of the Digit Span Backwards and Logical Memory Test. Before tests were administered, speech understanding was confirmed using an established protocol.^5^

1. *Digit Span Backwards (DSB)*.^6^ A test of attention in which participants were read a string of numbers ranging from 2 to 7 digits. For each number string, the participant was asked to repeat the numbers backwards. Two trials were administered for each digit span length. The score ranged from 0 to 12 and documented the number of trials in which the participant correctly stated the reversed number string.
2. *Boston Naming Test (BNT)*.^7^ A 30-item test that entailed naming common objects from a series of 30 line drawings. Participants were given 20 seconds to name the object in each drawing. The score ranged from 0 to 30 and indicated the number of objects correctly identified.
3. *Word Fluency Test (WFT)*.^8^ A measure of phonemic fluency in which participants were given 60 seconds to state as many words as possible that began with the letters F, A, or S. 60 seconds were given for each letter. The score denoted the total number of acceptable words stated.
4. *Animal Naming Score (ANS)*.^8^ A measure of semantic fluency that involved naming animals in 60 seconds. Names of extinct, imaginary, and magical animals were permitted. Credit was given for breeds, different names for males, females, or infants of the same species (e.g. bull, cow, calf) as well as superordinate and subordinate titles (e.g. dog and terrier). The score represents the total number of animals stated.
5. *Digit Symbol Substitution (DSS)*.^6^ A test of executive functioning in which participants were asked to translate numbers to symbols using a key. The score is a count of numbers correctly translated to symbols within 90-seconds.
6. *Trail Making Test A (TMTA)*.^9^ A test of processing speed in which participants were presented with numbers ranging from 1 to 25. Each number was placed in a separate circle and distributed haphazardly across a page. Participants were asked to draw lines connecting the numbers sequentially. The score indicated the number of seconds the participant took to complete the test. Participants who took longer than four minutes or who made more than 5 errors were given the maximum score of 240 seconds.
7. *Trail Making Test B (TMTB)*.^9^ A variation of the TMTA in which participants were presented with numbers ranging from 1 to 13 and letters ranging from A to L. Participants were asked to draw lines connecting the numbers and letters in a sequential, alternating fashion. The score represents the number of seconds required for the participant to complete the test and was calculated using the same rules applied to the TMTA.
8. *Incidental Learning (ILR)*.^10^ A test of delayed recall. Immediately following completion of the DSS, participants were asked to write down as many symbols as they could remember followed by the number paired with each symbol. The score ranged from 0 to 9 and separately documented the number of symbols and symbol-pairs recalled.
9. *Logical Memory Test (LMT)*.^6^ A test of recall from two short stories read aloud to the participant. Initially, participants were asked to recall the details immediately following the reading of each story. Participants were then informed there would be additional questions about these stories at a subsequent point in time. After a delay of approximately 20 minutes, the participants were again asked to recall the details of each story. The score reflected the number of details correctly recalled with a maximum score of 25 per story.
10. *Delayed Word Recall (DWR)*.^11^ A 10-word test of delayed episodic verbal memory in which participants were visually presented with 10 common nouns that they were asked to use in a sentence. After a five-minute delay, participants were given 60 seconds to recall the words. The score ranged from 0 to 10 and catalogued the number of words correctly recalled.

**Additional Baseline Measures**

Speech in noise ability was measured by the Quick Speech in Noise (QuickSIN) test.^12^ Communicative function was measured by the 10-item screening version of the Hearing Handicap Inventory for the Elderly (HHIE).^13,14^ Systolic blood pressure (SBP) and diastolic blood pressure (DBP) were measured using the Omron HEM-907 XL oscillometric automated sphygmomanometer (Omron Healthcare, Kyoto, Japan). Hypertension was defined as SBP ≥140 mmHg, DBP ≥90 mmHg, use of anti-hypertensive medication, or self-reported physician diagnosis. Diabetes was defined as fasting glucose ≥ 126 mg/dL, non-fasting glucose ≥200 mg/dL, use of glucose-lowering medication, or self-reported physician diagnosis. Current, former, or never use of cigarettes or alcohol were ascertained by self-report.

**Multiple Imputation**

To mitigate bias in estimates of cognitive decline caused by informative attrition,^15^ multiple imputation by chained equations^16^ was employed. A hundred imputed datasets were generated based on a two-stage analysis^17^ indicating that precision would be acceptable if a minimum of twelve datasets were analysed. The imputation model included baseline and follow-up measures of glial fibrillary acidic protein (GFAP), neurofilament light (NfL), and cognition. The imputation model also incorporated baseline measures of all covariates specified in the primary analysis model including randomisation, age, sex, recruitment source, field site, education, pure tone average, and the presence of apolipoprotein E ε4 alleles. Auxiliary variables^18^ added to the imputation model included race, MMSE, QuickSIN, HHIE, BMI, kidney disease, SBP, DBP, hypertension, diabetes, cigarette use, alcohol use, enrolment in ACHIEVE with a spouse or partner, and the time between baseline and follow-up assessments.

**Inverse Probability Weighting**

The primary analysis applied the intention-to-treat principle to estimate the effect of randomisation independent of treatment adherence.^19^ The effect of treatment adherence was estimated using per-protocol analyses, but these estimates can be biased if treatment adherence is associated with factors that are correlated with the primary outcome.^20,21^ To mitigate bias, the propensity of treatment adherence was estimated from a logistic regression model fit to imputed data.^22,23^ The propensity model included baseline measures of randomisation, age, sex, race, recruitment source, field site, education, cognition, MMSE, the presence of apolipoprotein E ε4 alleles, pure tone average, QuickSIN, HHIE, BMI, kidney disease, SBP, DBP, hypertension, diabetes, cigarette use, alcohol use, enrolment in ACHIEVE with a spouse or partner, and the time between baseline and follow-up assessments. The estimated propensities were used to generate unstabilised and stabilised weights. The unstabilised weights were used in unadjusted models and the stabilised weights were used in covariate-adjusted models to estimate the effect of treatment adherence conditional on the assumption of principal ignorability.^24^ To mitigate selection bias, a logistic regression model was used to estimate the probability that each ACHIEVE participant (n=977) would have a follow-up sample (n=552). The model included all measures used in the imputation model. The estimated probabilities were used to generate unstabilised and stabilised selection weights that were incorporated into intention-to-treat analyses.

# **eTable 1.** Demographic and Clinical Characteristics of ACHIEVE Participants Stratified by Recruitment Source and Inclusion in Primary Analytic Sample (n=977)

|  | **ACHIEVE (n=977)** | | | |  | **ARIC (n=238)** | |  |
| --- | --- | --- | --- | --- | --- | --- | --- | --- |
|  | **Total** | **De novo** | **ARIC** |  |  | **Excluded** | **Included** |  |
|  | **(n=977)** | **(n=739)** | **(n=238)** | **p** |  | **(n=74)** | **(n=164)** | **p** |
| Age, years (n=977) | 76·8 (4·0) | 76·1 (4·0) | 78·9 (2·9) | <0·0001 |  | 78·5 (3·1) | 78·1 (2·9) | 0·39 |
| Female sex (n=977) | 523 (53·5) | 376 (50·9) | 147 (61·8) | 0·0034 |  | 42 (56·8) | 105 (64·0) | 0·29 |
| Black race (n=977) | 112 (11·5) | 44 (6·0) | 68 (28·6) | <0·0001 |  | 24 (32·4) | 44 (26·8) | 0·38 |
| White race (n=977) | 858 (87·8) | 689 (93·2) | 169 (71·0) | <0·0001 |  | 49 (66·2) | 120 (73·2) | 0·27 |
| Field site (n=977) |  |  |  |  |  |  |  |  |
| Forsyth County, North Carolina | 236 (24·2) | 175 (23·7) | 61 (25·6) | 0·81 |  | 23 (31·1) | 38 (23·2) | 0·13 |
| Jackson, Mississippi | 243 (24·9) | 180 (24·4) | 63 (26·5) |  |  | 20 (27·0) | 43 (26·2) |  |
| Minneapolis, Minnesota | 236 (24·2) | 193 (26·1) | 43 (18·1) |  |  | 13 (17·6) | 30 (18·3) |  |
| Washington County, Maryland | 262 (26·8) | 191 (25·8) | 71 (29·8) |  |  | 18 (24·3) | 53 (32·3) |  |
| Education (n=976) |  |  |  |  |  |  |  |  |
| Less than high school | 37 (3·8) | 15 (2·0) | 22 (9·3) | 0·0073 |  | 5 (6·8) | 17 (10·4) | 0·27 |
| High school, GED, or vocational school | 418 (42·8) | 322 (43·6) | 96 (40·5) |  |  | 28 (38·4) | 68 (41·5) |  |
| Some college, graduate, or professional school | 521 (53·4) | 402 (54·4) | 119 (50·2) |  |  | 40 (54·8) | 79 (48·2) |  |
| Income, US$ (n=950) |  |  |  |  |  |  |  |  |
| Under $25,000 | 147 (15·5) | 87 (12·0) | 60 (26·7) | <0·0001 |  | 22 (32·4) | 38 (24·2) | 0·16 |
| $25,000-$49,999 | 283 (29·8) | 206 (28·4) | 77 (34·2) |  |  | 23 (33·8) | 54 (34·4) |  |
| $50,000-$74,999 | 210 (22·1) | 163 (22·5) | 47 (20·9) |  |  | 12 (17·6) | 35 (22·3) |  |
| $75,000-$100,000 | 140 (14·7) | 119 (16·4) | 21 (9·3) |  |  | 8 (11·8) | 13 (8·3) |  |
| Over $100,000 | 170 (17·9) | 150 (20·7) | 20 (8·9) |  |  | 3 (4·4) | 17 (10·8) |  |
| Enrolled as spousal pair (n=977) | 90 (9·2) | 67 (9·1) | 23 (9·7) | 0·78 |  | 7 (9·5) | 16 (9·8) | 0·94 |
| One or more apolipoprotein E ε4 alleles (n=908) | 224 (24·7) | 165 (24·3) | 59 (25·7) | 0·69 |  | 18 (25·7) | 41 (25·6) | 0·99 |
| Body mass index, kg/m^2^ (n=972) | 29·0 (5·5) | 28·9 (5·5) | 29·2 (5·4) | 0·49 |  | 29·4 (5·7) | 29·1 (5·4) | 0·64 |
| Diabetes (n=977) | 195 (20·0) | 127 (17·2) | 68 (28·6) | 0·0001 |  | 23 (31·1) | 45 (27·4) | 0·56 |
| Systolic blood pressure, mm HG (n=968) | 130·9 (17·3) | 131·6 (17·3) | 128·6 (17·0) | 0·025 |  | 128·9 (20·0) | 128·5 (15·5) | 0·88 |
| Diastolic blood pressure, mm HG (n=968) | 68·1 (10·5) | 69·0 (10·2) | 65·2 (10·8) | <0·0001 |  | 64·4 (11·2) | 65·5 (10·7) | 0·47 |
| Hypertension (n=974) | 651 (66·8) | 482 (65·2) | 169 (71·9) | 0·058 |  | 58 (78·4) | 111 (68·9) | 0·13 |
| Kidney disease (n=975) | 57 (5·8) | 39 (5·3) | 18 (7·6) | 0·19 |  | 6 (8·2) | 12 (7·3) | 0·81 |
| Alcohol use (n=977) |  |  |  |  |  |  |  |  |
| Current | 560 (57·3) | 445 (60·2) | 115 (48·3) | 0·0004 |  | 34 (45·9) | 81 (49·4) | 0·92 |
| Former | 238 (24·4) | 174 (23·5) | 64 (26·9) |  |  | 24 (32·4) | 40 (24·4) |  |
| Never | 179 (18·3) | 120 (16·2) | 59 (24·8) |  |  | 16 (21·6) | 43 (26·2) |  |
| Cigarette use (n=977) |  |  |  |  |  |  |  |  |
| Current | 25 (2·6) | 15 (2·0) | 10 (4·2) | 0·67 |  | 6 (8·1) | 4 (2·4) | 0·037 |
| Former | 443 (45·3) | 346 (46·8) | 97 (40·8) |  |  | 33 (44·6) | 64 (39·0) |  |
| Never | 509 (52·1) | 378 (51·2) | 131 (55·0) |  |  | 35 (47·3) | 96 (58·5) |  |
| Pure tone average, dB (n=977) | 39·42 (6·91) | 39·52 (6·98) | 39·12 (6·71) | 0·44 |  | 39·51 (6·96) | 38·94 (6·61) | 0·54 |
| Pure tone average ≥40 db (n=977) | 425 (43·5) | 326 (44·1) | 99 (41·6) | 0·50 |  | 31 (41·9) | 68 (41·5) | 0·95 |
| Hearing handicap inventory score (n=970) | 15·28 (9·75) | 16·32 (9·61) | 12·04 (9·50) | <0·0001 |  | 11·57 (9·60) | 12·26 (9·48) | 0·61 |
| Quick speech in noise (n=972) | 18·41 (5·22) | 18·52 (5·06) | 18·06 (5·68) | 0·23 |  | 17·25 (6·00) | 18·42 (5·51) | 0·14 |
| Mini-mental state examination score (n=977) | 28·22 (1·62) | 28·28 (1·57) | 28·01 (1·75) | 0·023 |  | 27·62 (1·80) | 28·18 (1·71) | 0·022 |
| Intervention drop-in (n=462) | 76 (16·5) | 67 (19·4) | 9 (7·8) | 0·0035 |  | 1 (3·3) | 8 (9·3) | 0·29 |
| Intervention drop-out (n=488) | 10 (2·0) | 5 (1·4) | 5 (4·2) | 0·059 |  | 2 (4·8) | 3 (3·8) | 0·81 |
| Time between randomisation  and follow-up, years (n=877) | 3·1 (0·2) | 3·0 (0·1) | 3·2 (0·2) | <0·0001 |  | 3·2 (0·3) | 3·2 (0·2) | 0·57 |

Abbreviations: ACHIEVE, Aging and Cognitive Health Evaluation in Elders Trial; ARIC, Atherosclerosis in Communities Cohort; dB, decibels; GED, General educational development credential; kg, kilograms; m, metres; mm HG, millimetres of mercury.

Data are n (%) or mean (SD). Denominators for percentages are based on the number of participants with complete data as indicated after each characteristic. Sex, race, education income, cigarette use, and alcohol use were self-reported. The presence of apolipoprotein E ε4 alleles was ascertained by the TaqMan assay (Applied Biosystems, Foster City, California). Diabetes and kidney disease were defined as present if the participant reported using medication or self-reported a physician diagnosis. Sitting blood pressure was measured using a random zero sphygmomanometer. Hypertension was defined as present based on the use of antihypertensive medication, systolic blood pressure greater than or equal to 140 mm Hg, or diastolic blood pressure greater than or equal to 90 mm Hg. Univariate differences assessed using χ2 tests, t tests, and Cochran–Armitage trend tests.

# **eTable 2.** Demographic and Clinical Characteristics of ACHIEVE Participants with Follow-Up Blood-Based Biomarkers Stratified by Randomly Assigned Treatment (n=552)

|  |  | **Total (n=552)** | |  | **ARIC (n=176)** | |  | **De novo (n=376)** | |  |
| --- | --- | --- | --- | --- | --- | --- | --- | --- | --- | --- |
|  |  | **Intervention** | **Control** |  | **Intervention** | **Control** |  | **Intervention** | **Control** |  |
|  | **All** | **(n=287)** | **(n=265)** | **p** | **(n=85)** | **(n=91)** | **p** | **(n=202)** | **(n=174)** | **p** |
| **Baseline** |  |  |  |  |  |  |  |  |  |  |
| Age, years (n=552) | 76·4 (3·9) | 76·2 (3·9) | 76·6 (3·9) | 0·24 | 78·3 (3·0) | 77·9 (2·8) | 0·31 | 75·4 (3·9) | 76·0 (4·3) | 0·16 |
| Female sex (n=552) | 306 (55·4) | 151 (52·6) | 155 (58·5) | 0·17 | 54 (63·5) | 60 (65·9) | 0·76 | 97 (48·0) | 95 (54·6) | 0·20 |
| Black race (n=552) | 76 (13·8) | 36 (12·5) | 40 (15·1) | 0·38 | 24 (28·2) | 26 (28·6) | 0·74 | 12 (5·9) | 14 (8·0) | 0·42 |
| White race (n=552) | 473 (85·7) | 250 (87·1) | 223 (84·2) | 0·32 | 61 (71·8) | 65 (71·4) | 0·74 | 189 (93·6) | 158 (90·8) | 0·32 |
| Field site (n=552) |  |  |  |  |  |  |  |  |  |  |
| Forsyth County, North Carolina | 114 (20·7) | 59 (20·6) | 55 (20·8) | 0·82 | 19 (22·4) | 21 (23·1) | 0·32 | 40 (19·8) | 34 (19·5) | 0·77 |
| Jackson, Mississippi | 148 (26·8) | 75 (26·1) | 73 (27·5) |  | 21 (24·7) | 26 (28·6) |  | 54 (26·7) | 47 (27·0) |  |
| Minneapolis, Minnesota | 114 (20·7) | 61 (21·3) | 53 (20·0) |  | 14 (16·5) | 18 (19·8) |  | 47 (23·3) | 35 (20·1) |  |
| Washington County, Maryland | 176 (31·9) | 92 (32·1) | 84 (31·7) |  | 31 (36·5) | 26 (28·6) |  | 61 (30·2) | 58 (33·3) |  |
| Education (n=552) |  |  |  |  |  |  |  |  |  |  |
| Less than high school | 22 (4·0) | 12 (4·2) | 10 (3·8) | 0·62 | 10 (11·8) | 8 (8·8) | 0·73 | 2 (1·0) | 2 (1·1) | 0·28 |
| High school, GED, or vocational school | 251 (45·5) | 126 (43·9) | 125 (47·2) |  | 35 (41·2) | 37 (40·7) |  | 91 (45·0) | 88 (50·6) |  |
| Some college, graduate, or professional school | 279 (50·5) | 149 (51·9) | 130 (49·1) |  | 40 (47·1) | 46 (50·5) |  | 109 (54·0) | 84 (48·3) |  |
| Income, US$ (n=540) |  |  |  |  |  |  |  |  |  |  |
| Under $25,000 | 82 (15·2) | 39 (14·0) | 43 (16·5) | 0·76 | 20 (25·0) | 21 (24·1) | 0·049 | 19 (9·5) | 22 (12·6) | 0·12 |
| $25,000-$49,999 | 166 (30·7) | 98 (35·1) | 68 (26·1) |  | 36 (45·0) | 21 (24·1) |  | 62 (31·2) | 47 (27·0) |  |
| $50,000-$74,999 | 124 (23·0) | 51 (18·3) | 73 (28·0) |  | 15 (18·8) | 21 (24·1) |  | 36 (18·1) | 52 (29·9) |  |
| $75,000-$100,000 | 79 (14·6) | 39 (14·0) | 40 (15·3) |  | 3 (3·8) | 13 (14·9) |  | 36 (18·1) | 27 (15·5) |  |
| Over $100,000 | 89 (16·5) | 52 (18·6) | 37 (14·2) |  | 6 (7·5) | 11 (12·6) |  | 46 (23·1) | 26 (14·9) |  |
| Enrolled as spousal pair (n=552) | 52 (9·4) | 20 (7·0) | 32 (12·1) | 0·040 | 5 (5·9) | 11 (12·1) | 0·17 | 15 (7·4) | 21 (12·1) | 0·13 |
| One or more apolipoprotein E ε4 alleles (n=536) | 131 (24·4) | 68 (24·6) | 63 (24·2) | 0·91 | 17 (21·0) | 25 (27·5) | 0·28 | 51 (26·2) | 38 (22·5) | 0·42 |
| Body mass index, kg/m^2^ (n=550) | 28·9 (5·5) | 29·0 (5·3) | 28·8 (5·6) | 0·68 | 29·6 (4·5) | 28·8 (5·9) | 0·24 | 28·8 (5·6) | 28·9 (5·4) | 0·90 |
| Diabetes (n=552) | 114 (20·7) | 59 (20·6) | 55 (20·8) | 0·95 | 24 (28·2) | 25 (27·5) | 0·89 | 35 (17·3) | 30 (17·2) | 0·98 |
| Systolic blood pressure, mm HG (n=546) | 129·9 (17·0) | 128·6 (17·8) | 131·3 (16·1) | 0·064 | 125·6 (16·7) | 131·1 (15·3) | 0·013 | 129·9 (18·2) | 131·4 (16·4) | 0·39 |
| Diastolic blood pressure, mm HG (n=546) | 68·1 (10·6) | 67·2 (11·0) | 69·1 (10·1) | 0·034 | 64·7 (11·0) | 66·3 (10·4) | 0·36 | 68·2 (10·9) | 70·5 (9·7) | 0·035 |
| Hypertension (n=549) | 358 (65·2) | 183 (63·8) | 175 (66·8) | 0·46 | 57 (67·1) | 63 (71·6) | 0·54 | 126 (62·4) | 112 (64·4) | 0·69 |
| Kidney disease (n=551) | 30 (5·4) | 16 (5·6) | 14 (5·3) | 0·87 | 7 (8·2) | 5 (5·5) | 0·44 | 9 (4·5) | 9 (5·2) | 0·75 |
| Alcohol use (n=552) |  |  |  |  |  |  |  |  |  |  |
| Current | 310 (56·2) | 160 (55·7) | 150 (56·6) | 0·96 | 39 (45·9) | 47 (51·6) | 0·99 | 121 (59·9) | 103 (59·2) | 0·85 |
| Former | 131 (23·7) | 70 (24·4) | 61 (23·0) |  | 26 (30·6) | 18 (19·8) |  | 44 (21·8) | 43 (24·7) |  |
| Never | 111 (20·1) | 57 (19·9) | 54 (20·4) |  | 20 (23·5) | 26 (28·6) |  | 37 (18·3) | 28 (16·1) |  |
| Cigarette use (n=552) |  |  |  |  |  |  |  |  |  |  |
| Current | 11 (2·0) | 8 (2·8) | 3 (1·1) | 0·32 | 4 (4·7) | 0 (0·0) | 0·17 | 4 (2·0) | 3 (1·7) | 0·81 |
| Former | 237 (42·9) | 125 (43·6) | 112 (42·3) |  | 34 (40·0) | 35 (38·5) |  | 91 (45·0) | 77 (44·3) |  |
| Never | 304 (55·1) | 154 (53·7) | 150 (56·6) |  | 47 (55·3) | 56 (61·5) |  | 107 (53·0) | 94 (54·0) |  |
| Pure tone average, dB (n=552) | 39·36 (6·72) | 39·48 (7·02) | 39·22 (6·38) | 0·64 | 39·61 (6·87) | 38·34 (6·18) | 0·24 | 39·43 (7·10) | 39·67 (6·46) | 0·73 |
| Pure tone average ≥40 db (n=552) | 242 (43·8) | 123 (42·9) | 119 (44·9) | 0·63 | 38 (44·7) | 36 (39·6) | 0·49 | 85 (42·1) | 83 (47·7) | 0·27 |
| Hearing handicap inventory score (n=547) | 14·92 (9·81) | 15·19 (10·19) | 14·62 (9·39) | 0·50 | 13·40 (10·45) | 11·58 (8·85) | 0·45 | 15·94 (10·01) | 16·23 (9·30) | 0·77 |
| Quick speech in noise (n=548) | 18·71 (5·14) | 18·49 (5·56) | 18·95 (4·64) | 0·30 | 18·01 (5·69) | 18·80 (5·10) | 0·25 | 18·70 (5·51) | 19·02 (4·40) | 0·53 |
| Mini-mental state examination score (n=552) | 28·30 (1·58) | 28·33 (1·58) | 28·27 (1·57) | 0·63 | 28·26 (1·73) | 28·14 (1·69) | 0·46 | 28·36 (1·52) | 28·33 (1·50) | 0·87 |
| Intervention drop-in (n=264) | 42 (15·8) |  | 42 (15·8) |  |  | 8 (8·8) |  |  | 34 (19·5) |  |
| Intervention drop-out (n=288) | 5 (1·7) | 5 (1·7) |  |  | 3 (3·5) |  |  | 2 (1·0) |  |  |
| Time between randomisation and  blood-based biomarker, years (n=552) | 4·1 (0·7) | 4·2 (0·7) | 4·0 (0·7) | 0·0022 | 3·5 (0·7) | 3·3 (0·4) | 0·017 | 4·5 (0·4) | 4·4 (0·4) | 0·041 |
|  |  |  |  |  |  |  |  |  |  |  |
|  |  |  |  |  |  |  |  |  |  |  |
|  |  |  |  |  |  |  |  |  |  |  |
|  |  |  |  |  |  |  |  |  |  |  |
|  |  |  |  |  |  |  |  |  |  |  |
|  |  | **Total (n=552)** | |  | **ARIC (n=176)** | |  | **De novo (n=376)** | |  |
|  |  | **Intervention** | **Control** |  | **Intervention** | **Control** |  | **Intervention** | **Control** |  |
|  | **All** | **(n=287)** | **(n=265)** | **p** | **(n=85)** | **(n=91)** | **p** | **(n=202)** | **(n=174)** | **p** |
| **Baseline and Follow-up** |  |  |  |  |  |  |  |  |  |  |
| Blood-based biomarkers in NPQ units, mean (SD) |  |  |  |  |  |  |  |  |  |  |
| Glial fibrillary acidic protein, mean (SD) |  |  |  |  |  |  |  |  |  |  |
| Baseline (n=164) |  |  |  |  | 13·06 (0·60) | 13·21 (0·62) | 0·10 |  |  |  |
| Follow-up (n=552) | 13·17 (0·71) | 13·14 (0·74) | 13·20 (0·68) | 0·35 | 13·05 (0·62) | 13·35 (0·65) | 0·0029 | 13·19 (0·76) | 13·14 (0·69) | 0·52 |
| Three-year change (n=164) |  |  |  |  | -0·01 (0·34) | 0·10  (0·27) | 0·029 |  |  |  |
| Neurofilament light chain, mean (SD) |  |  |  |  |  |  |  |  |  |  |
| Baseline (n=164) |  |  |  |  | 12·48 (0·77) | 12·50 (0·61) | 0·84 |  |  |  |
| Follow-up (n=552) | 12·45 (0·71) | 12·42 (0·72) | 12·49 (0·70) | 0·27 | 12·63 (0·76) | 12·83 (0·77) | 0·092 | 12·34 (0·70) | 12·32 (0·58) | 0·75 |
| Three-year change (n=164) |  |  |  |  | 0·09 (0·51) | 0·25 (0·45) | 0·035 |  |  |  |
| Phosphorylated tau-181, mean (SD) |  |  |  |  |  |  |  |  |  |  |
| Baseline (n=164) |  |  |  |  | 13·30 (0·52) | 13·29 (0·56) | 0·85 |  |  |  |
| Follow-up (n=552) | 13·35 (0·62) | 13·33 (0·63) | 13·37 (0·62) | 0·44 | 13·31 (0·63) | 13·47 (0·57) | 0·093 | 13·36 (0·62) | 13·33 (0·64) | 0·71 |
| Three-year change (n=164) |  |  |  |  | 0·01 (0·36) | 0·13 (0·36) | 0·028 |  |  |  |
| Phosphorylated tau-217, mean (SD) |  |  |  |  |  |  |  |  |  |  |
| Baseline (n=164) |  |  |  |  | 11·49 (0·47) | 11·64 (0·56) | 0·080 |  |  |  |
| Follow-up (n=552) | 11·60 (0·56) | 11·58 (0·56) | 11·63 (0·56) | 0·34 | 11·57 (0·55) | 11·79 (0·56) | 0·012 | 11·59 (0·56) | 11·55 (0·55) | 0·44 |
| Three-year change (n=164) |  |  |  |  | 0·05 (0·28) | 0·11 (0·23) | 0·17 |  |  |  |
| Phosphorylated tau-231, mean (SD) |  |  |  |  |  |  |  |  |  |  |
| Baseline (n=164) |  |  |  |  | 13·49 (0·57) | 13·56 (0·63) | 0·40 |  |  |  |
| Follow-up (n=552) | 13·60 (0·68) | 13·57 (0·68) | 13·65 (0·67) | 0·16 | 13·52 (0·65) | 13·77 (0·64) | 0·016 | 13·60 (0·69) | 13·59 (0·69) | 0·88 |
| Three-year change (n=164) |  |  |  |  | 0·03 (0·36) | 0·15 (0·34) | 0·030 |  |  |  |
| Blood-based biomarkers in standard deviations, mean (SD) |  |  |  |  |  |  |  |  |  |  |
| Glial fibrillary acidic protein, mean (SD) |  |  |  |  |  |  |  |  |  |  |
| Baseline (n=164) |  |  |  |  | -0·16 (0·93) | 0·08 (0·98) | 0·11 |  |  |  |
| Follow-up (n=552) | 0·01 (1·01) | -0·05 (0·99) | 0·06 (1·04) | 0·21 | -0·16 (0·96) | 0·29 (0·99) | 0·0035 | 0·01 (0·98) | -0·03 (1·05) | 0·69 |
| Three-year change (n=164) |  |  |  |  | -0·01 (0·54) | 0·15 (0·41) | 0·039 |  |  |  |
| Neurofilament light chain, mean (SD) |  |  |  |  |  |  |  |  |  |  |
| Baseline (n=164) |  |  |  |  | 0·00 (1·07) | 0·07 (0·89) | 0·65 |  |  |  |
| Follow-up (n=552) | -0·03 (1·03) | -0·08 (1·06) | 0·02 (0·99) | 0·24 | 0·23 (1·06) | 0·51 (0·97) | 0·079 | -0·19 (1·05) | -0·21 (0·90) | 0·89 |
| Three-year change (n=164) |  |  |  |  | 0·14 (0·67) | 0·33 (0·55) | 0·055 |  |  |  |
| Phosphorylated tau-181, mean (SD) |  |  |  |  |  |  |  |  |  |  |
| Baseline (n=164) |  |  |  |  | -0·03 (0·89) | -0·08 (0·92) | 0·73 |  |  |  |
| Follow-up (n=552) | 0·02 (1·01) | -0·02 (1·02) | 0·06 (1·01) | 0·36 | -0·06 (1·04) | 0·23 (0·91) | 0·059 | 0·03 (1·00) | -0·01 (1·06) | 0·71 |
| Three-year change (n=164) |  |  |  |  | -0·01 (0·61) | 0·23 (0·56) | 0·0097 |  |  |  |
| Phosphorylated tau-217, mean (SD) |  |  |  |  |  |  |  |  |  |  |
| Baseline (n=164) |  |  |  |  | -0·17 (0·94) | 0·09 (0·96) | 0·090 |  |  |  |
| Follow-up (n=552) | 0·01 (1·03) | -0·04 (1·02) | 0·06 (1·03) | 0·30 | -0·05 (1·01) | 0·37 (0·93) | 0·0058 | -0·01 (1·02) | -0·10 (1·06) | 0·41 |
| Three-year change (n=164) |  |  |  |  | 0·09 (0·57) | 0·22 (0·42) | 0·11 |  |  |  |
| Phosphorylated tau-231, mean (SD) |  |  |  |  |  |  |  |  |  |  |
| Baseline (n=164) |  |  |  |  | -0·14 (0·91) | -0·03 (0·93) | 0·45 |  |  |  |
| Follow-up (n=552) | 0·03 (1·02) | -0·03 (1·03) | 0·10 (1·01) | 0·12 | -0·10 (1·02) | 0·29 (0·93) | 0·012 | 0·02 (1·01) | 0·01 (1·05) | 0·91 |
| Three-year change (n=164) |  |  |  |  | 0·03 (0·58) | 0·23 (0·49) | 0·019 |  |  |  |
| Cognition, mean (SD) |  |  |  |  |  |  |  |  |  |  |
| Baseline (n=552) | 0·06 (0·89) | 0·09 (0·90) | 0·04 (0·87) | 0·49 | -0·27 (1·07) | -0·20 (1·00) | 0·68 | 0·24 (0·78) | 0·16 (0·77) | 0·34 |
| Follow-up (n=546) | -0·18 (1·03) | -0·12 (1·04) | -0·25 (1·02) | 0·16 | -0·52 (1·25) | -0·65 (1·16) | 0·48 | 0·04 (0·89) | -0·03 (0·87) | 0·43 |
| Three-year change (n=546) | -0·24 (0·47) | -0·21 (0·50) | -0·27 (0·45) | 0·15 | -0·26 (0·55) | -0·42 (0·44) | 0·034 | -0·19 (0·47) | -0·19 (0·43) | 0·97 |

Abbreviations: ACHIEVE, Aging and Cognitive Health Evaluation in Elders Trial; ARIC, Atherosclerosis in Communities Cohort; dB, decibels; GED, General educational development credential; kg, kilograms; m, metres; mm HG, millimetres of mercury; NULISA protein quantification; NPQ.

Data are n (%) or mean (SD). Denominators for percentages are based on the number of participants with complete data as indicated after each characteristic. Sex, race, education income, cigarette use, and alcohol use were self-reported. The presence of apolipoprotein E ε4 alleles was ascertained by the TaqMan assay (Applied Biosystems, Foster City, California). Diabetes and kidney disease were defined as present if the participant reported using medication or self-reported a physician diagnosis. Sitting blood pressure was measured using a random zero sphygmomanometer. Hypertension was defined as present based on the use of antihypertensive medication, systolic blood pressure greater than or equal to 140 mm Hg, or diastolic blood pressure greater than or equal to 90 mm Hg. Glial fibrillary acidic protein, neurofilament light chain, and phosphorylated tau at threonine 181, 217, and 231 were measured using the Alamar NUcleic acid Linked Immuno-Sandwich Assay (NULISA™) central nervous system 120 kit, which produces sample-specific NPQ units. Higher scores indicate greater neuroinflammation, neurodegeneration, or Alzheimer's disease pathology. A 1-unit change corresponds to a 2-fold increase in the concentration of the biomarker. A 2-unit change corresponds to a 4-fold increase in the concentration. To prevent outliers from biasing results, a rank-based inverse normal transformation was applied to each biomarker prior to statistical analysis. A factor score quantifying cognition was developed using a validated latent variable modelling approach and standardised to the baseline, with lower scores indicating worse cognitive function. Univariate differences assessed using χ2 tests, t tests, and Cochran–Armitage trend tests.

# **eTable 3. Intention-to-Treat Analysis of Three-Year Change in Pre-Specified Blood-Based Biomarkers and Cognition Among Participants with Baseline and Follow-Up Samples in ACHIEVE by Randomly Assigned Treatment (n=164)**

|  | **Model 1** | | **Model 2** | | **Model 3** | |
| --- | --- | --- | --- | --- | --- | --- |
|  | **Three-Year Change**  **in SD units** | **Difference**  **in SD units** | **Three-Year Change**  **in SD units** | **Difference**  **in SD units** | **Three-Year Change**  **in SD units** | **Difference**  **in SD units** |
|  | **β (95% CI)** | **β (95% CI)** | **β (95% CI)** | **β (95% CI)** | **β (95% CI)** | **β (95% CI)** |
| **Glial fibrillary acidic protein** | | | | | | |
| Control | 0·149 (0·046, 0·252) | -0·156 (-0·306, -0·007) | 0·149 (0·039, 0·260) | -0·175 (-0·322, -0·028) | 0·149 (0·038, 0·260) | -0·172 (-0·321, -0·024) |
| Hearing Intervention | -0·007 (-0·116, 0·101) | p = 0·041 | -0·026 (-0·144, 0·093) | p = 0·019 | -0·023 (-0·143, 0·097) | p = 0·023 |
| **Neurofilament light chain** | | | | | | |
| Control | 0·320 (0·192, 0·448) | -0·139 (-0·323, 0·045) | 0·330 (0·185, 0·475) | -0·159 (-0·348, 0·029) | 0·330 (0·186, 0·475) | -0·172 (-0·362, 0·017) |
| Hearing Intervention | 0·180 (0·048, 0·313) | p = 0·14 | 0·171 (0·022, 0·319) | p = 0·098 | 0·158 (0·008, 0·309) | p = 0·075 |
| **Cognition** | | | | | | |
| Control | -0·423 (-0·529, -0·317) | 0·174 (0·018, 0·330) | -0·448 (-0·559, -0·337) | 0·192 (0·043, 0·341) | -0·448 (-0·559, -0·336) | 0·192 (0·042, 0·342) |
| Hearing Intervention | -0·249 (-0·363, -0·135) | p = 0·029 | -0·256 (-0·378, -0·135) | p = 0·012 | -0·255 (-0·378, -0·133) | p = 0·012 |

Abbreviations: ACHIEVE, Aging and Cognitive Health Evaluation in Elders Trial; CI, confidence interval; SD, standard deviation.

Point estimates, 95% confidence intervals, and p values were calculated by fitting separate linear mixed effects models for each outcome to imputed data. Each model specified time from baseline as the timescale, included a random intercept and time slope, and employed an unstructured variance-covariance matrix. The first model generated crude, unadjusted estimates. The second model adjusted for baseline measures of age, sex, field site, education, hearing loss (pure tone average <40 dB vs ≥40 dB), and the presence of apolipoprotein E ε4 alleles. The third model integrated baseline measures of body mass index and kidney disease. An interaction was specified between each covariate and time. Glial fibrillary acidic protein and neurofilament light chain were measured using the Alamar NUcleic acid Linked Immuno-Sandwich Assay (NULISA™) central nervous system 120 kit. Higher scores indicate greater neuroinflammation or neurodegeneration. To prevent outliers from biasing results, a rank-based inverse normal transformation was applied to each biomarker prior to statistical analysis. A factor score quantifying cognition was developed using a validated latent variable modelling approach and standardised to the baseline, with lower scores indicating worse cognitive function.

# **eTable 4. Complete Case Intention-to-Treat Analysis of Three-Year Change in Pre-Specified Blood-Based Biomarkers and Cognition Among Participants with Baseline and Follow-Up Samples in ACHIEVE by Randomly Assigned Treatment (n=164)**

|  | **Model 1** | | **Model 2** | | **Model 3** | | |  |
| --- | --- | --- | --- | --- | --- | --- | --- | --- |
|  | **Three-Year Change**  **in SD units** | **Difference**  **in SD units** | **Three-Year Change**  **in SD units** | **Difference**  **in SD units** | | **Three-Year Change**  **in SD units** | **Difference**  **in SD units** | |
|  | **β (95% CI)** | **β (95% CI)** | **β (95% CI)** | **β (95% CI)** | | **β (95% CI)** | **β (95% CI)** | |
| **Glial fibrillary acidic protein** | | | | | | | | |
| Control | 0·149 (0·046, 0·252) | -0·156 (-0·306, -0·007) | 0·152 (0·041, 0·263) | -0·172 (-0·322, -0·022) | | 0·147 (0·035, 0·259) | -0·179 (-0·331, -0·027) | |
| Hearing Intervention | -0·007 (-0·116, 0·101) | p = 0·041 | -0·020 (-0·142, 0·101) | p = 0·025 | | -0·032 (-0·157, 0·093) | p = 0·021 | |
| **Neurofilament light chain** | | | | | | | | |
| Control | 0·320 (0·192, 0·448) | -0·139 (-0·323, 0·045) | 0·324 (0·177, 0·472) | -0·144 (-0·336, 0·049) | | 0·323 (0·175, 0·470) | -0·165 (-0·360, 0·031) | |
| Hearing Intervention | 0·180 (0·048, 0·313) | p = 0·14 | 0·181 (0·027, 0·335) | p = 0·14 | | 0·158 (-0·001, 0·316) | p = 0·097 | |
| **Cognition** | | | | | | | | |
| Control | -0·423 (-0·531, -0·316) | 0·176 (0·018, 0·335) | -0·446 (-0·558, -0·333) | 0·189 (0·035, 0·343) | | -0·448 (-0·561, -0·335) | 0·182 (0·026, 0·338) | |
| Hearing Intervention | -0·247 (-0·363, -0·131) | p = 0·029 | -0·257 (-0·382, -0·131) | p = 0·017 | | -0·266 (-0·395, -0·136) | p = 0·023 | |

Abbreviations: ACHIEVE, Aging and Cognitive Health Evaluation in Elders Trial; CI, confidence interval; SD, standard deviation.

Point estimates, 95% confidence intervals, and p values were calculated by fitting separate linear mixed effects models for each outcome to complete case data. Each model specified time from baseline as the timescale, included a random intercept and time slope, and employed an unstructured variance-covariance matrix. The first model generated crude, unadjusted estimates. The second model adjusted for baseline measures of age, sex, field site, education, hearing loss (pure tone average <40 dB vs ≥40 dB), and the presence of apolipoprotein E ε4 alleles. The third model integrated baseline measures of body mass index and kidney disease. An interaction was specified between each covariate and time. Glial fibrillary acidic protein and neurofilament light chain were measured using the Alamar NUcleic acid Linked Immuno-Sandwich Assay (NULISA™) central nervous system 120 kit. Higher scores indicate greater neuroinflammation or neurodegeneration. To prevent outliers from biasing results, a rank-based inverse normal transformation was applied to each biomarker prior to statistical analysis. A factor score quantifying cognition was developed using a validated latent variable modelling approach and standardised to the baseline, with lower scores indicating worse cognitive function.

# **eTable 5.** Intention-to-Treat Analysis of Three-Year Change in Untransformed Pre-Specified Blood-Based Biomarkers Among Participants with Baseline and Follow-Up Samples in ACHIEVE by Randomly Assigned Treatment (n=164)

|  | **Model 1** | | **Model 2** | | **Model 3** | |
| --- | --- | --- | --- | --- | --- | --- |
|  | **Three-Year Change**  **in NPQ units** | **Difference**  **in NPQ units** | **Three-Year Change**  **in NPQ units** | **Difference**  **in NPQ units** | **Three-Year Change**  **in NPQ units** | **Difference**  **in NPQ units** |
|  | **β (95% CI)** | **β (95% CI)** | **β (95% CI)** | **β (95% CI)** | **β (95% CI)** | **β (95% CI)** |
| **Glial fibrillary acidic protein** | | | | | | |
| Control | 0·100 (0·034, 0·165) | -0·106 (-0·201, -0·011) | 0·106 (0·034, 0·178) | -0·106 (-0·198, -0·013) | 0·107 (0·034, 0·179) | -0·104 (-0·197, -0·011) |
| Hearing Intervention | -0·006 (-0·075, 0·063) | p = 0·029 | 0·001 (-0·072, 0·073) | p = 0·025 | 0·003 (-0·071, 0·076) | p = 0·028 |
| **Neurofilament light chain** | | | | | | |
| Control | 0·241 (0·140, 0·342) | -0·125 (-0·271, 0·020) | 0·246 (0·132, 0·360) | -0·142 (-0·291, 0·008) | 0·246 (0·134, 0·358) | -0·155 (-0·303, -0·007) |
| Hearing Intervention | 0·116 (0·011, 0·220) | p = 0·091 | 0·104 (-0·014, 0·222) | p = 0·063 | 0·091 (-0·027, 0·209) | p = 0·040 |

Abbreviations: ACHIEVE, Aging and Cognitive Health Evaluation in Elders Trial; CI, confidence interval; NULISA protein quantification, NPQ.

Point estimates, 95% confidence intervals, and p values were calculated by fitting separate linear mixed effects models for each outcome to imputed data. Each model specified time from baseline as the timescale, included a random intercept and time slope, and employed an unstructured variance-covariance matrix. The first model generated crude, unadjusted estimates. The second model adjusted for baseline measures of age, sex, field site, education, hearing loss (pure tone average <40 dB vs ≥40 dB), and the presence of apolipoprotein E ε4 alleles. The third model integrated baseline measures of body mass index and kidney disease. An interaction was specified between each covariate and time. Glial fibrillary acidic protein and neurofilament light chain were measured using the Alamar NUcleic acid Linked Immuno-Sandwich Assay (NULISA™) central nervous system 120 kit, which produces sample-specific NPQ units. Higher scores indicate greater neuroinflammation or neurodegeneration. A 1-unit change corresponds to a 2-fold increase in the concentration of the biomarker. A 2-unit change corresponds to a 4-fold increase in the concentration. Concentrations are biomarker-specific and cannot be compared to each other.

# **eTable 6.** Complete Case Intention-to-Treat Analysis of Three-Year Change in Untransformed Pre-Specified Blood-Based Biomarkers Among Participants with Baseline and Follow-Up Samples in ACHIEVE by Randomly Assigned Treatment (n=164)

|  | **Model 1** | | **Model 2** | | **Model 3** | |
| --- | --- | --- | --- | --- | --- | --- |
|  | **Three-Year Change**  **in NPQ units** | **Difference**  **in NPQ units** | **Three-Year Change**  **in NPQ units** | **Difference**  **in NPQ units** | **Three-Year Change**  **in NPQ units** | **Difference**  **in NPQ units** |
|  | **β (95% CI)** | **β (95% CI)** | **β (95% CI)** | **β (95% CI)** | **β (95% CI)** | **β (95% CI)** |
| **Glial fibrillary acidic protein** | | | | | | |
| Control | 0·100 (0·034, 0·165) | -0·106 (-0·201, -0·011) | 0·098 (0·027, 0·169) | -0·116 (-0·212, -0·020) | 0·095 (0·024, 0·167) | -0·120 (-0·218, -0·023) |
| Hearing Intervention | -0·006 (-0·075, 0·063) | p = 0·029 | -0·018 (-0·095, 0·060) | p = 0·019 | -0·025 (-0·105, 0·055) | p = 0·016 |
| **Neurofilament light chain** | | | | | | |
| Control | 0·241 (0·140, 0·342) | -0·125 (-0·271, 0·020) | 0·241 (0·125, 0·357) | -0·129 (-0·281, 0·024) | 0·240 (0·125, 0·354) | -0·150 (-0·302, 0·003) |
| Hearing Intervention | 0·116 (0·011, 0·220) | p = 0·091 | 0·113 (-0·009, 0·234) | p = 0·097 | 0·090 (-0·034, 0·214) | p = 0·055 |

Abbreviations: ACHIEVE, Aging and Cognitive Health Evaluation in Elders Trial; CI, confidence interval; NULISA protein quantification, NPQ.

Point estimates, 95% confidence intervals, and p values were calculated by fitting separate linear mixed effects models for each outcome to complete case data. Each model specified time from baseline as the timescale, included a random intercept and time slope, and employed an unstructured variance-covariance matrix. The first model generated crude, unadjusted estimates. The second model adjusted for baseline measures of age, sex, field site, education, hearing loss (pure tone average <40 dB vs ≥40 dB), and the presence of apolipoprotein E ε4 alleles. The third model integrated baseline measures of body mass index and kidney disease. An interaction was specified between each covariate and time. Glial fibrillary acidic protein and neurofilament light chain were measured using the Alamar NUcleic acid Linked Immuno-Sandwich Assay (NULISA™) central nervous system 120 kit, which produces sample-specific NPQ units. Higher scores indicate greater neuroinflammation or neurodegeneration. A 1-unit change corresponds to a 2-fold increase in the concentration of the biomarker. A 2-unit change corresponds to a 4-fold increase in the concentration. Concentrations are biomarker-specific and cannot be compared to each other.

# **eTable 7.** Multivariate Intention-to-Treat Analysis of Three-Year Change in Pre-Specified Blood-Based Biomarkers Among Participants with Baseline and Follow-Up Samples in ACHIEVE by Randomly Assigned Treatment (n=164)

|  | **Model 1** | | **Model 2** | | **Model 3** | |
| --- | --- | --- | --- | --- | --- | --- |
|  | **Three-Year Change**  **in SD units** | **Difference**  **in SD units** | **Three-Year Change**  **in SD units** | **Difference**  **in SD units** | **Three-Year Change**  **in SD units** | **Difference**  **in SD units** |
|  | **β (95% CI)** | **β (95% CI)** | **β (95% CI)** | **β (95% CI)** | **β (95% CI)** | **β (95% CI)** |
| **Imputed** | | | | | | |
| Control | 0·236 (0·153, 0·319) | -0·158 (-0·277, -0·038) | 0·241 (0·150, 0·332) | -0·176 (-0·296, -0·056) | 0·240 (0·149, 0·331) | -0·180 (-0·300, -0·059) |
| Hearing Intervention | 0·078 (-0·008, 0·164) | p = 0·0099 | 0·065 (-0·031, 0·161) | p = 0·0042 | 0·061 (-0·036, 0·157) | p = 0·0036 |
| **Complete case** | | | | | | |
| Control | 0·236 (0·153, 0·319) | -0·158 (-0·277, -0·038) | 0·239 (0·147, 0·332) | -0·165 (-0·287, -0·043) | 0·235 (0·142, 0·327) | -0·178 (-0·302, -0·054) |
| Hearing Intervention | 0·078 (-0·008, 0·164) | p = 0·0099 | 0·074 (-0·024, 0·173) | p = 0·0084 | 0·056 (-0·045, 0·157) | p = 0·0049 |

Abbreviations: ACHIEVE, Aging and Cognitive Health Evaluation in Elders Trial; CI, confidence interval; SD, standard deviation.

Point estimates, 95% confidence intervals, and p values were calculated by fitting a single linear mixed effects model for glial fibrillary acidic protein and neurofilament light chain. The model specified time from baseline as the timescale, included a random intercept and time slope for each participant, added a random intercept for the biomarkers, and employed an unstructured variance-covariance matrix. The first model generated crude, unadjusted estimates. The second model adjusted for baseline measures of age, sex, field site, education, hearing loss (pure tone average <40 dB vs ≥40 dB), and the presence of apolipoprotein E ε4 alleles. The third model integrated baseline measures of body mass index and kidney disease. An interaction was specified between each covariate and time. Glial fibrillary acidic protein and neurofilament light chain were measured using the Alamar NUcleic acid Linked Immuno-Sandwich Assay (NULISA™) central nervous system 120 kit. Higher scores indicate greater neuroinflammation and neurodegeneration. To prevent outliers from biasing results, a rank-based inverse normal transformation was applied to each biomarker prior to statistical analysis.

# **eTable 8.** Per-Protocol Analysis **of Three-Year Change in Pre-Specified Blood-Based Biomarkers and Cognition Among Participants with Baseline and Follow-Up Samples in ACHIEVE by Randomly Assigned Treatment (n=150)**

|  | **Model 1** | | **Model 2** | | **Model 3** | |
| --- | --- | --- | --- | --- | --- | --- |
|  | **Three-Year Change**  **in SD units** | **Difference**  **in SD units** | **Three-Year Change**  **in SD units** | **Difference**  **in SD units** | **Three-Year Change**  **in SD units** | **Difference**  **in SD units** |
|  | **β (95% CI)** | **β (95% CI)** | **β (95% CI)** | **β (95% CI)** | **β (95% CI)** | **β (95% CI)** |
| **Glial fibrillary acidic protein** | | | | | | |
| Control | 0·157 (0·047, 0·267) | -0·178 (-0·337, -0·019) | 0·153 (0·034, 0·272) | -0·199 (-0·357, -0·041) | 0·156 (0·036, 0·276) | -0·197 (-0·356, -0·037) |
| Hearing Intervention | -0·021 (-0·136, 0·094) | p = 0·029 | -0·046 (-0·172, 0·081) | p = 0·014 | -0·040 (-0·169, 0·088) | p = 0·016 |
| **Neurofilament light chain** | | | | | | |
| Control | 0·283 (0·157, 0·408) | -0·098 (-0·276, 0·080) | 0·280 (0·136, 0·424) | -0·096 (-0·282, 0·090) | 0·285 (0·142, 0·428) | -0·115 (-0·301, 0·071) |
| Hearing Intervention | 0·185 (0·059, 0·310) | p = 0·28 | 0·184 (0·041, 0·328) | p = 0·31 | 0·170 (0·025, 0·315) | p = 0·23 |
| **Cognition** | | | | | | |
| Control | -0·400 (-0·512, -0·287) | 0·157 (-0·005, 0·320) | -0·431 (-0·546, -0·315) | 0·164 (0·010, 0·317) | -0·433 (-0·548, -0·317) | 0·171 (0·017, 0·325) |
| Hearing Intervention | -0·242 (-0·360, -0·124) | p = 0·058 | -0·267 (-0·390, -0·144) | p = 0·037 | -0·262 (-0·387, -0·138) | p = 0·030 |

Abbreviations: ACHIEVE, Aging and Cognitive Health Evaluation in Elders Trial; CI, confidence interval; SD, standard deviation.

Point estimates, 95% confidence intervals, and p values were calculated by fitting separate linear mixed effects models for each outcome to imputed data. Each model specified time from baseline as the timescale, included a random intercept and time slope, and employed an unstructured variance-covariance matrix. The first model generated crude, unadjusted estimates. The second model adjusted for baseline measures of age, sex, field site, education, hearing loss (pure tone average <40 dB vs ≥40 dB), and the presence of apolipoprotein E ε4 alleles. The third model integrated baseline measures of body mass index and kidney disease. An interaction was specified between each covariate and time. Glial fibrillary acidic protein and neurofilament light chain were measured using the Alamar NUcleic acid Linked Immuno-Sandwich Assay (NULISA™) central nervous system 120 kit. Higher scores indicate greater neuroinflammation or neurodegeneration. To prevent outliers from biasing results, a rank-based inverse normal transformation was applied to each biomarker prior to statistical analysis. A factor score quantifying cognition was developed using a validated latent variable modelling approach and standardised to the baseline, with lower scores indicating worse cognitive function.

# **eTable 9.** Complier Average Causal Effect Analysis of Three-Year Change in Pre-Specified Blood-Based Biomarkers and Cognition Among Participants with Baseline and Follow-Up Samples in ACHIEVE by Randomly Assigned Treatment (n=164)

|  | **Model 1** | | **Model 2** | | **Model 3** | |
| --- | --- | --- | --- | --- | --- | --- |
|  | **Three-Year Change**  **in SD units** | **Difference**  **in SD units** | **Three-Year Change**  **in SD units** | **Difference**  **in SD units** | **Three-Year Change**  **in SD units** | **Difference**  **in SD units** |
|  | **β (95% CI)** | **β (95% CI)** | **β (95% CI)** | **β (95% CI)** | **β (95% CI)** | **β (95% CI)** |
| **Glial fibrillary acidic protein** | | | | | | |
| Control | 0·148 (0·045, 0·250) | -0·155 (-0·303, -0·007) | 0·149 (0·037, 0·261) | -0·174 (-0·323, -0·026) | 0·149 (0·036, 0·261) | -0·171 (-0·321, -0·021) |
| Hearing Intervention | -0·007 (-0·115, 0·100) | p = 0·040 | -0·025 (-0·145, 0·094) | p = 0·022 | -0·022 (-0·143, 0·099) | p = 0·026 |
| **Neurofilament light chain** | | | | | | |
| Control | 0·306 (0·180, 0·431) | -0·149 (-0·331, 0·033) | 0·313 (0·170, 0·456) | -0·165 (-0·351, 0·021) | 0·316 (0·174, 0·458) | -0·170 (-0·356, 0·016) |
| Hearing Intervention | 0·157 (0·025, 0·288) | p = 0·11 | 0·148 (0·000, 0·296) | p = 0·083 | 0·146 (-0·002, 0·294) | p = 0·073 |
| **Cognition** | | | | | | |
| Control | -0·420 (-0·527, -0·313) | 0·173 (0·017, 0·329) | -0·442 (-0·554, -0·331) | 0·189 (0·040, 0·338) | -0·441 (-0·552, -0·329) | 0·188 (0·038, 0·338) |
| Hearing Intervention | -0·247 (-0·361, -0·133) | p = 0·030 | -0·253 (-0·375, -0·132) | p = 0·013 | -0·252 (-0·375, -0·130) | p = 0·014 |

Abbreviations: ACHIEVE, Aging and Cognitive Health Evaluation in Elders Trial; CI, confidence interval; SD, standard deviation.

Point estimates, 95% confidence intervals, and p values were calculated by fitting separate weighted linear mixed effects models for each outcome to imputed data. Each model specified time from baseline as the timescale, included a random intercept and time slope, and employed an unstructured variance-covariance matrix. Weights estimated the probability of complying with the intervention protocol if assigned to treatment. The first model generated crude, unadjusted estimates. The second model adjusted for baseline measures of age, sex, field site, education, hearing loss (pure tone average <40 dB vs ≥40 dB), and the presence of apolipoprotein E ε4 alleles. The third model integrated baseline measures of body mass index and kidney disease. An interaction was specified between each covariate and time. Glial fibrillary acidic protein and neurofilament light chain were measured using the Alamar NUcleic acid Linked Immuno-Sandwich Assay (NULISA™) central nervous system 120 kit. Higher scores indicate greater neuroinflammation or neurodegeneration. To prevent outliers from biasing results, a rank-based inverse normal transformation was applied to each biomarker prior to statistical analysis. A factor score quantifying cognition was developed using a validated latent variable modelling approach and standardised to the baseline, with lower scores indicating worse cognitive function.

# **eTable 10. Selection Weighted Intention-to-Treat Analysis of Three-Year Change in Pre-Specified Blood-Based Biomarkers and Cognition Among Participants with Baseline and Follow-Up Samples in ACHIEVE by Randomly Assigned Treatment (n=164)**

|  | **Model 1** | | **Model 2** | | **Model 3** | |
| --- | --- | --- | --- | --- | --- | --- |
|  | **Three-Year Change**  **in SD units** | **Difference**  **in SD units** | **Three-Year Change**  **in SD units** | **Difference**  **in SD units** | **Three-Year Change**  **in SD units** | **Difference**  **in SD units** |
|  | **β (95% CI)** | **β (95% CI)** | **β (95% CI)** | **β (95% CI)** | **β (95% CI)** | **β (95% CI)** |
| **Glial fibrillary acidic protein** | | | | | | |
| Control | 0·149 (0·047, 0·251) | -0·157 (-0·305, -0·009) | 0·149 (0·039, 0·260) | -0·175 (-0·322, -0·028) | 0·149 (0·038, 0·261) | -0·172 (-0·321, -0·024) |
| Hearing Intervention | -0·008 (-0·114, 0·099) | p = 0·038 | -0·026 (-0·145, 0·093) | p = 0·019 | -0·023 (-0·143, 0·098) | p = 0·023 |
| **Neurofilament light chain** | | | | | | |
| Control | 0·325 (0·193, 0·456) | -0·179 (-0·368, 0·010) | 0·324 (0·179, 0·468) | -0·185 (-0·375, 0·004) | 0·324 (0·181, 0·468) | -0·194 (-0·384, -0·003) |
| Hearing Intervention | 0·146 (0·011, 0·282) | p = 0·064 | 0·139 (-0·013, 0·290) | p = 0·056 | 0·131 (-0·022, 0·283) | p = 0·046 |
| **Cognition** | | | | | | |
| Control | -0·423 (-0·530, -0·316) | 0·176 (0·019, 0·334) | -0·448 (-0·561, -0·334) | 0·189 (0·034, 0·344) | -0·450 (-0·564, -0·336) | 0·183 (0·026, 0·340) |
| Hearing Intervention | -0·247 (-0·362, -0·131) | p = 0·028 | -0·259 (-0·386, -0·132) | p = 0·018 | -0·267 (-0·397, -0·137) | p = 0·023 |

Abbreviations: ACHIEVE, Aging and Cognitive Health Evaluation in Elders Trial; CI, confidence interval; SD, standard deviation.

Point estimates, 95% confidence intervals, and p values were calculated by fitting separate weighted linear mixed effects models for each outcome to imputed data. Each model specified time from baseline as the timescale, included a random intercept and time slope, and employed an unstructured variance-covariance matrix. Weights estimated the probability of having an analysed sample. The first model generated crude, unadjusted estimates. The second model adjusted for baseline measures of age, sex, field site, education, hearing loss (pure tone average <40 dB vs ≥40 dB), and the presence of apolipoprotein E ε4 alleles. The third model integrated baseline measures of body mass index and kidney disease. An interaction was specified between each covariate and time. Glial fibrillary acidic protein and neurofilament light chain were measured using the Alamar NUcleic acid Linked Immuno-Sandwich Assay (NULISA™) central nervous system 120 kit. Higher scores indicate greater neuroinflammation or neurodegeneration. To prevent outliers from biasing results, a rank-based inverse normal transformation was applied to each biomarker prior to statistical analysis. A factor score quantifying cognition was developed using a validated latent variable modelling approach and standardised to the baseline, with lower scores indicating worse cognitive function.

# **eTable 11. Intention-to-Treat Analysis of Three-Year Change in Exploratory Blood-Based Biomarkers of Alzheimer's Disease Pathology Among Participants with Baseline and Follow-Up Samples in ACHIEVE by Randomly Assigned Treatment (n=164)**

|  | **Model 1** | | **Model 2** | | **Model 3** | |
| --- | --- | --- | --- | --- | --- | --- |
|  | **Three-Year Change**  **in SD units** | **Difference**  **in SD units** | **Three-Year Change**  **in SD units** | **Difference**  **in SD units** | **Three-Year Change**  **in SD units** | **Difference**  **in SD units** |
|  | **β (95% CI)** | **β (95% CI)** | **β (95% CI)** | **β (95% CI)** | **β (95% CI)** | **β (95% CI)** |
| **Phosphorylated tau 181** | | | | | | |
| Control | 0·228 (0·103, 0·354) | -0·238 (-0·420, -0·056) | 0·222 (0·085, 0·360) | -0·245 (-0·427, -0·062) | 0·222 (0·086, 0·359) | -0·244 (-0·426, -0·061) |
| Hearing Intervention | -0·010 (-0·142, 0·122) | p = 0·011 | -0·022 (-0·169, 0·125) | p = 0·0087 | -0·021 (-0·169, 0·126) | p = 0·0088 |
| **Phosphorylated tau 217** | | | | | | |
| Control | 0·208 (0·105, 0·310) | -0·127 (-0·274, 0·021) | 0·201 (0·085, 0·316) | -0·116 (-0·266, 0·034) | 0·201 (0·085, 0·316) | -0·116 (-0·267, 0·035) |
| Hearing Intervention | 0·081 (-0·025, 0·187) | p = 0·091 | 0·085 (-0·034, 0·203) | p = 0·13 | 0·085 (-0·035, 0·204) | p = 0·13 |
| **Phosphorylated tau 231** | | | | | | |
| Control | 0·231 (0·118, 0·345) | -0·198 (-0·362, -0·033) | 0·255 (0·132, 0·378) | -0·199 (-0·363, -0·035) | 0·254 (0·131, 0·377) | -0·201 (-0·366, -0·037) |
| Hearing Intervention | 0·034 (-0·085, 0·153) | p = 0·019 | 0·056 (-0·076, 0·188) | p = 0·017 | 0·052 (-0·081, 0·186) | p = 0·016 |

Abbreviations: ACHIEVE, Aging and Cognitive Health Evaluation in Elders Trial; CI, confidence interval; SD, standard deviation.

Point estimates, 95% confidence intervals, and p values were calculated by fitting separate linear mixed effects models for each outcome to imputed data. Each model specified time from baseline as the timescale, included a random intercept and time slope, and employed an unstructured variance-covariance matrix. The first model generated crude, unadjusted estimates. The second model adjusted for baseline measures of age, sex, field site, education, hearing loss (pure tone average <40 dB vs ≥40 dB), and the presence of apolipoprotein E ε4 alleles. The third model integrated baseline measures of body mass index and kidney disease. An interaction was specified between each covariate and time. Phosphorylated tau at threonine 181, 217, and 231 was measured using the Alamar NUcleic acid Linked Immuno-Sandwich Assay (NULISA™) central nervous system 120 kit. Higher scores indicate greater Alzheimer's disease pathology. To prevent outliers from biasing results, a rank-based inverse normal transformation was applied to each biomarker prior to statistical analysis.

# **eTable 12. Complete Case Intention-to-Treat Analysis of Three-Year Change in Exploratory Blood-Based Biomarkers of Alzheimer's Disease Pathology Among Participants with Baseline and Follow-Up Samples in ACHIEVE by Randomly Assigned Treatment (n=164)**

|  | **Model 1** | | **Model 2** | | **Model 3** | | |  |
| --- | --- | --- | --- | --- | --- | --- | --- | --- |
|  | **Three-Year Change**  **in SD units** | **Difference**  **in SD units** | **Three-Year Change**  **in SD units** | **Difference**  **in SD units** | | **Three-Year Change**  **in SD units** | **Difference**  **in SD units** | |
|  | **β (95% CI)** | **β (95% CI)** | **β (95% CI)** | **β (95% CI)** | | **β (95% CI)** | **β (95% CI)** | |
| **Phosphorylated tau 181** | | | | | | | | |
| Control | 0·228 (0·103, 0·354) | -0·238 (-0·420, -0·056) | 0·237 (0·102, 0·373) | -0·220 (-0·404, -0·037) | | 0·241 (0·105, 0·376) | -0·209 (-0·394, -0·025) | |
| Hearing Intervention | -0·010 (-0·142, 0·122) | p = 0·011 | 0·017 (-0·132, 0·166) | p = 0·019 | | 0·031 (-0·120, 0·183) | p = 0·026 | |
| **Phosphorylated tau 217** | | | | | | | | |
| Control | 0·208 (0·105, 0·310) | -0·127 (-0·274, 0·021) | 0·211 (0·093, 0·329) | -0·115 (-0·269, 0·039) | | 0·209 (0·090, 0·329) | -0·118 (-0·275, 0·038) | |
| Hearing Intervention | 0·081 (-0·025, 0·187) | p = 0·091 | 0·096 (-0·027, 0·219) | p = 0·14 | | 0·091 (-0·036, 0·218) | p = 0·14 | |
| **Phosphorylated tau 231** | | | | | | | | |
| Control | 0·231 (0·118, 0·345) | -0·198 (-0·362, -0·033) | 0·268 (0·144, 0·393) | -0·179 (-0·347, -0·011) | | 0·270 (0·145, 0·394) | -0·176 (-0·345, -0·007) | |
| Hearing Intervention | 0·034 (-0·085, 0·153) | p = 0·019 | 0·090 (-0·047, 0·226) | p = 0·037 | | 0·093 (-0·046, 0·232) | p = 0·042 | |

Abbreviations: ACHIEVE, Aging and Cognitive Health Evaluation in Elders Trial; CI, confidence interval; SD, standard deviation.

Point estimates, 95% confidence intervals, and p values were calculated by fitting separate linear mixed effects models for each outcome to complete case data. Each model specified time from baseline as the timescale, included a random intercept and time slope, and employed an unstructured variance-covariance matrix. The first model generated crude, unadjusted estimates. The second model adjusted for baseline measures of age, sex, field site, education, hearing loss (pure tone average <40 dB vs ≥40 dB), and the presence of apolipoprotein E ε4 alleles. The third model integrated baseline measures of body mass index and kidney disease. An interaction was specified between each covariate and time. Phosphorylated tau at threonine 181, 217, and 231 was measured using the Alamar NUcleic acid Linked Immuno-Sandwich Assay (NULISA™) central nervous system 120 kit. Higher scores indicate greater Alzheimer's disease pathology. To prevent outliers from biasing results, a rank-based inverse normal transformation was applied to each biomarker prior to statistical analysis.

# **eTable 13.** Intention-to-Treat Analysis of Three-Year Change in Untransformed Exploratory Blood-Based Biomarkers of Alzheimer's Disease Pathology Among Participants with Baseline and Follow-Up Samples in ACHIEVE by Randomly Assigned Treatment (n=164)

|  | **Model 1** | | **Model 2** | | **Model 3** | |
| --- | --- | --- | --- | --- | --- | --- |
|  | **Three-Year Change**  **in NPQ units** | **Difference**  **in NPQ units** | **Three-Year Change**  **in NPQ units** | **Difference**  **in NPQ units** | **Three-Year Change**  **in NPQ units** | **Difference**  **in NPQ units** |
|  | **β (95% CI)** | **β (95% CI)** | **β (95% CI)** | **β (95% CI)** | **β (95% CI)** | **β (95% CI)** |
| **Phosphorylated tau 181** | | | | | | |
| Control | 0·135 (0·058, 0·211) | -0·124 (-0·234, -0·013) | 0·125 (0·039, 0·210) | -0·120 (-0·229, -0·010) | 0·125 (0·041, 0·210) | -0·122 (-0·231, -0·013) |
| Hearing Intervention | 0·011 (-0·069, 0·091) | p = 0·028 | 0·005 (-0·081, 0·091) | p = 0·032 | 0·003 (-0·083, 0·089) | p = 0·028 |
| **Phosphorylated tau 217** | | | | | | |
| Control | 0·110 (0·055, 0·165) | -0·059 (-0·138, 0·021) | 0·108 (0·047, 0·169) | -0·051 (-0·132, 0·029) | 0·107 (0·047, 0·168) | -0·055 (-0·136, 0·025) |
| Hearing Intervention | 0·051 (-0·006, 0·108) | p = 0·15 | 0·056 (-0·008, 0·121) | p = 0·21 | 0·052 (-0·012, 0·116) | p = 0·18 |
| **Phosphorylated tau 231** | | | | | | |
| Control | 0·147 (0·071, 0·223) | -0·121 (-0·228, -0·013) | 0·167 (0·084, 0·251) | -0·117 (-0·223, -0·010) | 0·168 (0·085, 0·251) | -0·122 (-0·228, -0·016) |
| Hearing Intervention | 0·026 (-0·050, 0·103) | p = 0·028 | 0·051 (-0·033, 0·135) | p = 0·033 | 0·046 (-0·038, 0·130) | p = 0·024 |

Abbreviations: ACHIEVE, Aging and Cognitive Health Evaluation in Elders Trial; CI, confidence interval; NULISA protein quantification, NPQ.

Point estimates, 95% confidence intervals, and p values were calculated by fitting separate linear mixed effects models for each outcome to imputed data. Each model specified time from baseline as the timescale, included a random intercept and time slope, and employed an unstructured variance-covariance matrix. The first model generated crude, unadjusted estimates. The second model adjusted for baseline measures of age, sex, field site, education, hearing loss (pure tone average <40 dB vs ≥40 dB), and the presence of apolipoprotein E ε4 alleles. The third model integrated baseline measures of body mass index and kidney disease. An interaction was specified between each covariate and time. Phosphorylated tau at threonine 181, 217, and 231 was measured using the Alamar NUcleic acid Linked Immuno-Sandwich Assay (NULISA™) central nervous system 120 kit, which produces sample-specific NPQ units. Higher scores indicate greater Alzheimer's disease pathology. A 1-unit change corresponds to a 2-fold increase in the concentration of the biomarker. A 2-unit change corresponds to a 4-fold increase in the concentration. Concentrations are biomarker-specific and cannot be compared to each other.

# **eTable 14.** Complete Case Intention-to-Treat Analysis of Three-Year Change in Untransformed Exploratory Blood-Based Biomarkers of Alzheimer's Disease Pathology Among Participants with Baseline and Follow-Up Samples in ACHIEVE by Randomly Assigned Treatment (n=164)

|  | **Model 1** | | **Model 2** | | **Model 3** | |
| --- | --- | --- | --- | --- | --- | --- |
|  | **Three-Year Change**  **in NPQ units** | **Difference**  **in NPQ units** | **Three-Year Change**  **in NPQ units** | **Difference**  **in NPQ units** | **Three-Year Change**  **in NPQ units** | **Difference**  **in NPQ units** |
|  | **β (95% CI)** | **β (95% CI)** | **β (95% CI)** | **β (95% CI)** | **β (95% CI)** | **β (95% CI)** |
| **Phosphorylated tau 181** | | | | | | |
| Control | 0·135 (0·058, 0·211) | -0·124 (-0·234, -0·013) | 0·142 (0·058, 0·226) | -0·115 (-0·229, -0·002) | 0·144 (0·061, 0·227) | -0·111 (-0·224, 0·002) |
| Hearing Intervention | 0·011 (-0·069, 0·091) | p = 0·028 | 0·027 (-0·065, 0·119) | p = 0·047 | 0·033 (-0·060, 0·126) | p = 0·055 |
| **Phosphorylated tau 217** | | | | | | |
| Control | 0·110 (0·055, 0·165) | -0·059 (-0·138, 0·021) | 0·114 (0·051, 0·176) | -0·051 (-0·134, 0·032) | 0·113 (0·050, 0·175) | -0·056 (-0·140, 0·027) |
| Hearing Intervention | 0·051 (-0·006, 0·108) | p = 0·15 | 0·063 (-0·004, 0·130) | p = 0·23 | 0·057 (-0·012, 0·125) | p = 0·19 |
| **Phosphorylated tau 231** | | | | | | |
| Control | 0·147 (0·071, 0·223) | -0·121 (-0·228, -0·013) | 0·176 (0·091, 0·262) | -0·111 (-0·220, -0·001) | 0·181 (0·096, 0·265) | -0·111 (-0·220, -0·001) |
| Hearing Intervention | 0·026 (-0·050, 0·103) | p = 0·028 | 0·066 (-0·022, 0·153) | p = 0·048 | 0·070 (-0·019, 0·159) | p = 0·047 |

Abbreviations: ACHIEVE, Aging and Cognitive Health Evaluation in Elders Trial; CI, confidence interval; NULISA protein quantification, NPQ.

Point estimates, 95% confidence intervals, and p values were calculated by fitting separate linear mixed effects models for each outcome to complete case data. Each model specified time from baseline as the timescale, included a random intercept and time slope, and employed an unstructured variance-covariance matrix. The first model generated crude, unadjusted estimates. The second model adjusted for baseline measures of age, sex, field site, education, hearing loss (pure tone average <40 dB vs ≥40 dB), and the presence of apolipoprotein E ε4 alleles. The third model integrated baseline measures of body mass index and kidney disease. An interaction was specified between each covariate and time. Phosphorylated tau at threonine 181, 217, and 231 was measured using the Alamar NUcleic acid Linked Immuno-Sandwich Assay (NULISA™) central nervous system 120 kit, which produces sample-specific NPQ units. Higher scores indicate greater Alzheimer's disease pathology. A 1-unit change corresponds to a 2-fold increase in the concentration of the biomarker. A 2-unit change corresponds to a 4-fold increase in the concentration. Concentrations are biomarker-specific and cannot be compared to each other.

# **eTable 15.** Multivariate Intention-to-Treat Analysis of Three-Year Change in Exploratory Blood-Based Biomarkers of Alzheimer's Disease Pathology Among Participants with Baseline and Follow-Up Samples in ACHIEVE by Randomly Assigned Treatment (n=164)

|  | **Model 1** | | **Model 2** | | **Model 3** | |
| --- | --- | --- | --- | --- | --- | --- |
|  | **Three-Year Change**  **in SD units** | **Difference**  **in SD units** | **Three-Year Change**  **in SD units** | **Difference**  **in SD units** | **Three-Year Change**  **in SD units** | **Difference**  **in SD units** |
|  | **β (95% CI)** | **β (95% CI)** | **β (95% CI)** | **β (95% CI)** | **β (95% CI)** | **β (95% CI)** |
| **Imputed** | | | | | | |
| Control | 0·223 (0·157, 0·289) | -0·190 (-0·285, -0·095) | 0·227 (0·155, 0·298) | -0·189 (-0·284, -0·094) | 0·226 (0·154, 0·297) | -0·189 (-0·285, -0·094) |
| Hearing Intervention | 0·033 (-0·036, 0·102) | p = 0·0001 | 0·038 (-0·039, 0·114) | p < 0·0001 | 0·036 (-0·040, 0·113) | p < 0·0001 |
| **Complete case** | | | | | | |
| Control | 0·223 (0·157, 0·289) | -0·190 (-0·285, -0·095) | 0·240 (0·168, 0·312) | -0·174 (-0·271, -0·078) | 0·241 (0·169, 0·313) | -0·170 (-0·267, -0·073) |
| Hearing Intervention | 0·033 (-0·036, 0·102) | p = 0·0001 | 0·065 (-0·013, 0·144) | p = 0·0004 | 0·071 (-0·009, 0·150) | p = 0·0006 |

Abbreviations: ACHIEVE, Aging and Cognitive Health Evaluation in Elders Trial; CI, confidence interval; SD, standard deviation.

Point estimates, 95% confidence intervals, and p values were calculated by fitting a single linear mixed effects model for phosphorylated tau at threonine 181, 217, and 231. The model specified time from baseline as the timescale, included a random intercept and time slope for each participant, added a random intercept for the biomarkers, and employed an unstructured variance-covariance matrix. The first model generated crude, unadjusted estimates. The second model adjusted for baseline measures of age, sex, field site, education, hearing loss (pure tone average <40 dB vs ≥40 dB), and the presence of apolipoprotein E ε4 alleles. The third model integrated baseline measures of body mass index and kidney disease. An interaction was specified between each covariate and time. Phosphorylated tau at threonine 181, 217, and 231 was measured using the Alamar NUcleic acid Linked Immuno-Sandwich Assay (NULISA™) central nervous system 120 kit. Higher scores indicate greater Alzheimer's disease pathology. To prevent outliers from biasing results, a rank-based inverse normal transformation was applied to each biomarker prior to statistical analysis.

# **eTable 16. Intention-to-Treat Analysis of Differences in Pre-Specified Blood-Based Biomarkers and Cognition Among Participants with Follow-Up Samples in ACHIEVE by Randomly Assigned Treatment and Recruitment Source (n=552)**

|  | **Model 1** | | **Model 2** | | **Model 3** | |
| --- | --- | --- | --- | --- | --- | --- |
|  | **Value** | **Difference** | **Value** | **Difference** | **Value** | **Difference** |
|  | **β (95% CI)** | **β (95% CI)** | **β (95% CI)** | **β (95% CI)** | **β (95% CI)** | **β (95% CI)** |
|  | **Total (n=552)** | | | | | |
| **Glial fibrillary acidic protein** | | | | | | |
| Control | 0·066 (-0·058, 0·190) | -0·116 (-0·285, 0·053) | 0·049 (-0·064, 0·163) | -0·083 (-0·244, 0·077) | 0·048 (-0·065, 0·162) | -0·081 (-0·240, 0·078) |
| Hearing Intervention | -0·050 (-0·164, 0·065) | p = 0·18 | -0·034 (-0·145, 0·077) | p = 0·31 | -0·033 (-0·141, 0·075) | p = 0·32 |
| **Neurofilament light chain** | | | | | | |
| Control | 0·034 (-0·081, 0·149) | -0·124 (-0·292, 0·044) | 0·005 (-0·099, 0·109) | -0·069 (-0·224, 0·086) | 0·006 (-0·093, 0·105) | -0·069 (-0·218, 0·080) |
| Hearing Intervention | -0·090 (-0·212, 0·032) | p = 0·15 | -0·063 (-0·177, 0·050) | p = 0·39 | -0·064 (-0·174, 0·047) | p = 0·36 |
| **Cognition** | | | | | | |
| Control | -0·252 (-0·375, -0·129) | 0·130 (-0·042, 0·302) | -0·220 (-0·324, -0·115) | 0·079 (-0·067, 0·224) | -0·221 (-0·325, -0·116) | 0·081 (-0·065, 0·226) |
| Hearing Intervention | -0·122 (-0·242, -0·002) | p = 0·14 | -0·141 (-0·239, -0·042) | p = 0·29 | -0·140 (-0·238, -0·042) | p = 0·28 |
|  | **ARIC (n=176)** | | | | | |
| **Glial fibrillary acidic protein** | | | | | | |
| Control | 0·177 (-0·025, 0·379) | -0·451 (-0·748, -0·154) | 0·071 (-0·136, 0·279) | -0·429 (-0·718, -0·139) | 0·089 (-0·119, 0·296) | -0·410 (-0·687, -0·132) |
| Hearing Intervention | -0·274 (-0·505, -0·043) | p = 0·0029 | -0·357 (-0·602, -0·113) | p = 0·0037 | -0·321 (-0·560, -0·081) | p = 0·0038 |
| **Neurofilament light chain** | | | | | | |
| Control | 0·375 (0·176, 0·573) | -0·322 (-0·622, -0·021) | 0·255 (0·050, 0·460) | -0·354 (-0·649, -0·058) | 0·280 (0·090, 0·469) | -0·363 (-0·633, -0·093) |
| Hearing Intervention | 0·053 (-0·189, 0·295) | p = 0·036 | -0·099 (-0·365, 0·167) | p = 0·019 | -0·083 (-0·332, 0·165) | p = 0·0084 |
| **Cognition** | | | | | | |
| Control | -0·639 (-0·883, -0·394) | 0·111 (-0·255, 0·478) | -0·508 (-0·747, -0·269) | 0·172 (-0·126, 0·469) | -0·516 (-0·751, -0·280) | 0·186 (-0·112, 0·484) |
| Hearing Intervention | -0·527 (-0·836, -0·219) | p = 0·55 | -0·336 (-0·616, -0·057) | p = 0·26 | -0·330 (-0·612, -0·047) | p = 0·22 |
|  | **De Novo (n=376)** | | | | | |
| **Glial fibrillary acidic protein** | | | | | | |
| Control | -0·003 (-0·166, 0·160) | 0·035 (-0·171, 0·240) | 0·006 (-0·154, 0·165) | 0·092 (-0·096, 0·280) | 0·007 (-0·154, 0·167) | 0·094 (-0·093, 0·280) |
| Hearing Intervention | 0·032 (-0·109, 0·173) | p = 0·74 | 0·097 (-0·060, 0·255) | p = 0·34 | 0·101 (-0·054, 0·255) | p = 0·32 |
| **Neurofilament light chain** | | | | | | |
| Control | -0·199 (-0·340, -0·058) | 0·012 (-0·185, 0·209) | -0·165 (-0·295, -0·035) | 0·067 (-0·112, 0·245) | -0·162 (-0·288, -0·036) | 0·071 (-0·103, 0·245) |
| Hearing Intervention | -0·187 (-0·337, -0·038) | p = 0·91 | -0·098 (-0·234, 0·037) | p = 0·46 | -0·091 (-0·224, 0·043) | p = 0·42 |
| **Cognition** | | | | | | |
| Control | -0·028 (-0·166, 0·110) | 0·072 (-0·107, 0·251) | -0·080 (-0·205, 0·044) | 0·040 (-0·118, 0·198) | -0·081 (-0·206, 0·044) | 0·040 (-0·119, 0·198) |
| Hearing Intervention | 0·044 (-0·086, 0·175) | p = 0·43 | -0·040 (-0·152, 0·072) | p = 0·62 | -0·041 (-0·154, 0·071) | p = 0·62 |

Abbreviations: ACHIEVE, Aging and Cognitive Health Evaluation in Elders Trial; ARIC, Atherosclerosis in Communities Cohort; CI, confidence interval.

Point estimates, 95% confidence intervals, and p values were calculated by fitting separate linear regression models for each outcome to imputed data. The first model adjusted for the time in years between randomisation and the follow-up assessment. The second model added baseline measures of age, sex, recruitment source, field site, education, hearing loss (pure tone average <40 dB vs ≥40 dB), and the presence of apolipoprotein E ε4 alleles. The third model integrated baseline measures of body mass index and kidney disease. Glial fibrillary acidic protein and neurofilament light chain were measured using the Alamar NUcleic acid Linked Immuno-Sandwich Assay (NULISA™) central nervous system 120 kit. Higher scores indicate greater neuroinflammation or neurodegeneration. To prevent outliers from biasing results, a rank-based inverse normal transformation was applied to each biomarker prior to statistical analysis. A factor score quantifying cognition was developed using a validated latent variable modelling approach and standardised to the baseline, with lower scores indicating worse cognitive function.

# **eTable 17. Complete Case Intention-to-Treat Analysis of Differences in Pre-Specified Blood-Based Biomarkers and Cognition Among Participants with Follow-Up Samples in ACHIEVE by Randomly Assigned Treatment and Recruitment Source (n=552)**

|  | **Model 1** | | **Model 2** | | **Model 3** | |
| --- | --- | --- | --- | --- | --- | --- |
|  | **Value** | **Difference** | **Value** | **Difference** | **Value** | **Difference** |
|  | **β (95% CI)** | **β (95% CI)** | **β (95% CI)** | **β (95% CI)** | **β (95% CI)** | **β (95% CI)** |
|  | **Total (n=552)** | | | | | |
| **Glial fibrillary acidic protein** | | | | | | |
| Control | 0·066 (-0·058, 0·190) | -0·108 (-0·277, 0·062) | 0·112 (-0·002, 0·227) | -0·087 (-0·250, 0·076) | 0·110 (-0·005, 0·225) | -0·087 (-0·248, 0·075) |
| Hearing Intervention | -0·042 (-0·157, 0·074) | p = 0·21 | 0·025 (-0·089, 0·140) | p = 0·30 | 0·023 (-0·089, 0·135) | p = 0·30 |
| **Neurofilament light chain** | | | | | | |
| Control | 0·033 (-0·082, 0·148) | -0·127 (-0·296, 0·041) | 0·034 (-0·070, 0·138) | -0·056 (-0·215, 0·104) | 0·037 (-0·062, 0·137) | -0·064 (-0·217, 0·089) |
| Hearing Intervention | -0·094 (-0·217, 0·028) | p = 0·14 | -0·022 (-0·140, 0·097) | p = 0·49 | -0·026 (-0·142, 0·089) | p = 0·41 |
| **Cognition** | | | | | | |
| Control | -0·256 (-0·379, -0·134) | 0·141 (-0·031, 0·313) | -0·211 (-0·316, -0·106) | 0·088 (-0·058, 0·235) | -0·216 (-0·320, -0·111) | 0·099 (-0·047, 0·245) |
| Hearing Intervention | -0·115 (-0·235, 0·005) | p = 0·11 | -0·123 (-0·225, -0·021) | p = 0·24 | -0·117 (-0·218, -0·015) | p = 0·18 |
|  | **ARIC (n=176)** | | | | | |
| **Glial fibrillary acidic protein** | | | | | | |
| Control | 0·176 (-0·026, 0·379) | -0·431 (-0·734, -0·127) | 0·093 (-0·107, 0·294) | -0·457 (-0·761, -0·153) | 0·092 (-0·109, 0·293) | -0·438 (-0·728, -0·149) |
| Hearing Intervention | -0·254 (-0·494, -0·014) | p = 0·0054 | -0·364 (-0·617, -0·110) | p = 0·0032 | -0·346 (-0·594, -0·099) | p = 0·0030 |
| **Neurofilament light chain** | | | | | | |
| Control | 0·376 (0·179, 0·574) | -0·326 (-0·629, -0·022) | 0·311 (0·107, 0·514) | -0·380 (-0·692, -0·068) | 0·317 (0·129, 0·504) | -0·410 (-0·694, -0·126) |
| Hearing Intervention | 0·051 (-0·195, 0·296) | p = 0·035 | -0·069 (-0·363, 0·225) | p = 0·017 | -0·094 (-0·367, 0·179) | p = 0·0046 |
| **Cognition** | | | | | | |
| Control | -0·638 (-0·882, -0·394) | 0·139 (-0·233, 0·510) | -0·486 (-0·715, -0·258) | 0·268 (-0·032, 0·568) | -0·490 (-0·718, -0·263) | 0·290 (-0·013, 0·593) |
| Hearing Intervention | -0·042 (-0·157, 0·074) | p = 0·21 | 0·025 (-0·089, 0·140) | p = 0·30 | 0·023 (-0·089, 0·135) | p = 0·30 |
|  | **De Novo (n=376)** | | | | | |
| **Glial fibrillary acidic protein** | | | | | | |
| Control | -0·003 (-0·166, 0·160) | 0·035 (-0·171, 0·240) | 0·062 (-0·098, 0·223) | 0·073 (-0·117, 0·262) | 0·071 (-0·091, 0·232) | 0·075 (-0·113, 0·263) |
| Hearing Intervention | 0·032 (-0·109, 0·173) | p = 0·74 | 0·135 (-0·025, 0·295) | p = 0·45 | 0·145 (-0·012, 0·303) | p = 0·43 |
| **Neurofilament light chain** | | | | | | |
| Control | -0·199 (-0·340, -0·058) | 0·012 (-0·185, 0·209) | -0·145 (-0·276, -0·014) | 0·078 (-0·103, 0·259) | -0·132 (-0·261, -0·004) | 0·080 (-0·095, 0·256) |
| Hearing Intervention | -0·187 (-0·337, -0·038) | p = 0·91 | -0·067 (-0·204, 0·071) | p = 0·40 | -0·052 (-0·188, 0·084) | p = 0·37 |
| **Cognition** | | | | | | |
| Control | -0·033 (-0·171, 0·105) | 0·072 (-0·107, 0·250) | -0·063 (-0·189, 0·063) | 0·022 (-0·138, 0·181) | -0·067 (-0·193, 0·059) | 0·027 (-0·133, 0·187) |
| Hearing Intervention | 0·039 (-0·091, 0·169) | p = 0·43 | -0·041 (-0·156, 0·073) | p = 0·79 | -0·040 (-0·156, 0·076) | p = 0·74 |

Abbreviations: ACHIEVE, Aging and Cognitive Health Evaluation in Elders Trial; ARIC, Atherosclerosis in Communities Cohort; CI, confidence interval.

Point estimates, 95% confidence intervals, and p values were calculated by fitting separate linear regression models for each outcome to complete case data. The first model adjusted for the time in years between randomisation and the follow-up assessment. The second model added baseline measures of age, sex, recruitment source, field site, education, hearing loss (pure tone average <40 dB vs ≥40 dB), and the presence of apolipoprotein E ε4 alleles. The third model integrated baseline measures of body mass index and kidney disease. Glial fibrillary acidic protein and neurofilament light chain were measured using the Alamar NUcleic acid Linked Immuno-Sandwich Assay (NULISA™) central nervous system 120 kit. Higher scores indicate greater neuroinflammation or neurodegeneration. To prevent outliers from biasing results, a rank-based inverse normal transformation was applied to each biomarker prior to statistical analysis. A factor score quantifying cognition was developed using a validated latent variable modelling approach and standardised to the baseline, with lower scores indicating worse cognitive function.

# **eTable 18. Intention-to-Treat Analysis of Differences in Untransformed Pre-Specified Blood-Based Biomarkers Among Participants with Follow-Up Samples in ACHIEVE by Randomly Assigned Treatment and Recruitment Source (n=552)**

|  | **Model 1** | | **Model 2** | | **Model 3** | |
| --- | --- | --- | --- | --- | --- | --- |
|  | **NPQ units** | **Difference** | **NPQ units** | **Difference** | **NPQ units** | **Difference** |
|  | **β (95% CI)** | **β (95% CI)** | **β (95% CI)** | **β (95% CI)** | **β (95% CI)** | **β (95% CI)** |
|  | **Total (n=552)** | | | | | |
| **Glial fibrillary acidic protein** | | | | | | |
| Control | 13·204 (13·123, 13·286) | -0·064 (-0·181, 0·053) | 13·199 (13·123, 13·274) | -0·047 (-0·155, 0·062) | 13·198 (13·122, 13·274) | -0·046 (-0·153, 0·062) |
| Hearing Intervention | 13·141 (13·056, 13·225) | p = 0·28 | 13·152 (13·073, 13·232) | p = 0·40 | 13·153 (13·075, 13·231) | p = 0·41 |
| **Neurofilament light chain** | | | | | | |
| Control | 12·494 (12·413, 12·575) | -0·083 (-0·199, 0·033) | 12·472 (12·399, 12·545) | -0·045 (-0·153, 0·062) | 12·472 (12·404, 12·541) | -0·046 (-0·148, 0·056) |
| Hearing Intervention | 12·411 (12·328, 12·493) | p = 0·16 | 12·426 (12·349, 12·504) | p = 0·41 | 12·426 (12·352, 12·501) | p = 0·38 |
|  | **ARIC (n=176)** | | | | | |
| **Glial fibrillary acidic protein** | | | | | | |
| Control | 13·275 (13·144, 13·407) | -0·302 (-0·496, -0·108) | 13·205 (13·070, 13·340) | -0·286 (-0·474, -0·098) | 13·217 (13·082, 13·352) | -0·275 (-0·456, -0·094) |
| Hearing Intervention | 12·973 (12·822, 13·125) | p = 0·0023 | 12·919 (12·760, 13·079) | p = 0·0029 | 12·942 (12·785, 13·099) | p = 0·0029 |
| **Neurofilament light chain** | | | | | | |
| Control | 12·734 (12·585, 12·883) | -0·238 (-0·463, -0·013) | 12·651 (12·501, 12·801) | -0·261 (-0·485, -0·037) | 12·671 (12·534, 12·807) | -0·273 (-0·473, -0·073) |
| Hearing Intervention | 12·496 (12·326, 12·666) | p = 0·038 | 12·390 (12·197, 12·583) | p = 0·023 | 12·398 (12·220, 12·576) | p = 0·0075 |
|  | **De Novo (n=376)** | | | | | |
| **Glial fibrillary acidic protein** | | | | | | |
| Control | 13·171 (13·058, 13·284) | 0·041 (-0·102, 0·183) | 13·208 (13·058, 13·357) | 0·080 (-0·053, 0·213) | 13·208 (13·058, 13·359) | 0·081 (-0·051, 0·214) |
| Hearing Intervention | 13·212 (13·087, 13·337) | p = 0·58 | 13·288 (13·109, 13·466) | p = 0·24 | 13·290 (13·113, 13·467) | p = 0·23 |
| **Neurofilament light chain** | | | | | | |
| Control | 12·323 (12·232, 12·413) | 0·020 (-0·108, 0·149) | 12·346 (12·262, 12·430) | 0·056 (-0·061, 0·174) | 12·348 (12·266, 12·430) | 0·060 (-0·054, 0·173) |
| Hearing Intervention | 12·343 (12·244, 12·442) | p = 0·76 | 12·402 (12·310, 12·494) | p = 0·35 | 12·408 (12·317, 12·498) | p = 0·31 |

Abbreviations: ACHIEVE, Aging and Cognitive Health Evaluation in Elders Trial; ARIC, Atherosclerosis in Communities Cohort; CI, confidence interval; NULISA protein quantification, NPQ.

Point estimates, 95% confidence intervals, and p values were calculated by fitting separate linear regression models for each outcome to imputed data. The first model adjusted for the time in years between randomisation and the follow-up assessment. The second model added baseline measures of age, sex, recruitment source, field site, education, hearing loss (pure tone average <40 dB vs ≥40 dB), and the presence of apolipoprotein E ε4 alleles. The third model integrated baseline measures of body mass index and kidney disease. Glial fibrillary acidic protein and neurofilament light chain were measured using the Alamar NUcleic acid Linked Immuno-Sandwich Assay (NULISA™) central nervous system 120 kit, which produces sample-specific NPQ units. Higher scores indicate greater neuroinflammation or neurodegeneration. A 1-unit change corresponds to a 2-fold increase in the concentration of the biomarker. A 2-unit change corresponds to a 4-fold increase in the concentration. Concentrations are biomarker-specific and cannot be compared to each other.

# **eTable 19. Complete Case Intention-to-Treat Analysis of Differences in Untransformed Pre-Specified Blood-Based Biomarkers Among Participants with Follow-Up Samples in ACHIEVE by Randomly Assigned Treatment and Recruitment Source (n=552)**

|  | **Model 1** | | **Model 2** | | **Model 3** | |
| --- | --- | --- | --- | --- | --- | --- |
|  | **NPQ units** | **Difference** | **NPQ units** | **Difference** | **NPQ units** | **Difference** |
|  | **β (95% CI)** | **β (95% CI)** | **β (95% CI)** | **β (95% CI)** | **β (95% CI)** | **β (95% CI)** |
|  | **Total (n=552)** | | | | | |
| **Glial fibrillary acidic protein** | | | | | | |
| Control | 13·204 (13·123, 13·285) | -0·058 (-0·176, 0·059) | 13·239 (13·163, 13·316) | -0·049 (-0·159, 0·062) | 13·238 (13·161, 13·315) | -0·048 (-0·159, 0·062) |
| Hearing Intervention | 13·146 (13·061, 13·231) | p = 0·33 | 13·191 (13·106, 13·276) | p = 0·39 | 13·190 (13·105, 13·274) | p = 0·39 |
| **Neurofilament light chain** | | | | | | |
| Control | 12·493 (12·412, 12·574) | -0·085 (-0·202, 0·031) | 12·491 (12·418, 12·565) | -0·035 (-0·146, 0·075) | 12·494 (12·425, 12·564) | -0·041 (-0·146, 0·063) |
| Hearing Intervention | 12·408 (12·325, 12·490) | p = 0·15 | 12·456 (12·375, 12·538) | p = 0·53 | 12·453 (12·375, 12·531) | p = 0·44 |
|  | **ARIC (n=176)** | | | | | |
| **Glial fibrillary acidic protein** | | | | | | |
| Control | 13·275 (13·143, 13·407) | -0·290 (-0·489, -0·091) | 13·220 (13·090, 13·349) | -0·305 (-0·502, -0·109) | 13·219 (13·089, 13·349) | -0·294 (-0·482, -0·106) |
| Hearing Intervention | 12·985 (12·827, 13·143) | p = 0·0042 | 12·914 (12·750, 13·079) | p = 0·0023 | 12·925 (12·764, 13·086) | p = 0·0022 |
| **Neurofilament light chain** | | | | | | |
| Control | 12·735 (12·587, 12·884) | -0·242 (-0·470, -0·015) | 12·689 (12·538, 12·840) | -0·282 (-0·520, -0·044) | 12·695 (12·560, 12·830) | -0·308 (-0·521, -0·096) |
| Hearing Intervention | 12·493 (12·320, 12·666) | p = 0·037 | 12·407 (12·193, 12·621) | p = 0·021 | 12·387 (12·191, 12·582) | p = 0·0044 |
|  | **De Novo (n=376)** | | | | | |
| **Glial fibrillary acidic protein** | | | | | | |
| Control | 13·171 (13·058, 13·284) | 0·041 (-0·102, 0·183) | 13·246 (13·096, 13·395) | 0·066 (-0·068, 0·201) | 13·251 (13·101, 13·402) | 0·068 (-0·066, 0·201) |
| Hearing Intervention | 13·212 (13·087, 13·337) | p = 0·58 | 13·312 (13·132, 13·492) | p = 0·33 | 13·319 (13·139, 13·499) | p = 0·32 |
| **Neurofilament light chain** | | | | | | |
| Control | 12·323 (12·232, 12·413) | 0·020 (-0·108, 0·149) | 12·356 (12·271, 12·441) | 0·067 (-0·053, 0·186) | 12·365 (12·281, 12·448) | 0·068 (-0·047, 0·183) |
| Hearing Intervention | 12·343 (12·244, 12·442) | p = 0·76 | 12·423 (12·329, 12·516) | p = 0·27 | 12·433 (12·341, 12·524) | p = 0·25 |

Abbreviations: ACHIEVE, Aging and Cognitive Health Evaluation in Elders Trial; ARIC, Atherosclerosis in Communities Cohort; CI, confidence interval; NULISA protein quantification, NPQ.

Point estimates, 95% confidence intervals, and p values were calculated by fitting separate linear regression models for each outcome to complete case data. The first model adjusted for the time in years between randomisation and the follow-up assessment. The second model added baseline measures of age, sex, recruitment source, field site, education, hearing loss (pure tone average <40 dB vs ≥40 dB), and the presence of apolipoprotein E ε4 alleles. The third model integrated baseline measures of body mass index and kidney disease. Glial fibrillary acidic protein and neurofilament light chain were measured using the Alamar NUcleic acid Linked Immuno-Sandwich Assay (NULISA™) central nervous system 120 kit, which produces sample-specific NPQ units. Higher scores indicate greater neuroinflammation or neurodegeneration. A 1-unit change corresponds to a 2-fold increase in the concentration of the biomarker. A 2-unit change corresponds to a 4-fold increase in the concentration. Concentrations are biomarker-specific and cannot be compared to each other.

# **eTable 20.** Multivariate **Intention-to-Treat Analysis of Differences in Pre-Specified Blood-Based Biomarkers Among Participants with Follow-Up Samples in ACHIEVE by Randomly Assigned Treatment and Recruitment Source (n=552)**

|  | **Model 1** | | **Model 2** | | **Model 3** | |
| --- | --- | --- | --- | --- | --- | --- |
|  | **Value** | **Difference** | **Value** | **Difference** | **Value** | **Difference** |
|  | **β (95% CI)** | **β (95% CI)** | **β (95% CI)** | **β (95% CI)** | **β (95% CI)** | **β (95% CI)** |
|  | **Total (n=552)** | | | | | |
| **Imputed** | | | | | | |
| Control | 0·050 (-0·050, 0·151) | -0·120 (-0·264, 0·024) | 0·028 (-0·061, 0·116) | -0·076 (-0·209, 0·057) | 0·027 (-0·059, 0·114) | -0·075 (-0·204, 0·054) |
| Hearing Intervention | -0·070 (-0·173, 0·033) | p = 0·11 | -0·048 (-0·145, 0·049) | p = 0·26 | -0·048 (-0·141, 0·045) | p = 0·25 |
| **Complete case** | | | | | | |
| Control | 0·050 (-0·050, 0·150) | -0·118 (-0·262, 0·027) | 0·073 (-0·017, 0·163) | -0·071 (-0·209, 0·066) | 0·074 (-0·015, 0·162) | -0·075 (-0·208, 0·058) |
| Hearing Intervention | -0·068 (-0·172, 0·036) | p = 0·11 | 0·002 (-0·100, 0·103) | p = 0·31 | -0·002 (-0·100, 0·097) | p = 0·27 |
|  | **ARIC (n=176)** | | | | | |
| **Imputed** | | | | | | |
| Control | 0·276 (0·108, 0·444) | -0·386 (-0·643, -0·130) | 0·163 (-0·004, 0·331) | -0·391 (-0·642, -0·140) | 0·184 (0·024, 0·345) | -0·386 (-0·617, -0·155) |
| Hearing Intervention | -0·111 (-0·318, 0·097) | p = 0·0031 | -0·228 (-0·446, -0·010) | p = 0·0022 | -0·202 (-0·410, 0·006) | p = 0·0010 |
| **Complete case** | | | | | | |
| Control | 0·276 (0·108, 0·445) | -0·378 (-0·639, -0·117) | 0·202 (0·038, 0·366) | -0·418 (-0·685, -0·152) | 0·204 (0·047, 0·361) | -0·424 (-0·669, -0·180) |
| Hearing Intervention | -0·102 (-0·315, 0·111) | p = 0·0045 | -0·216 (-0·454, 0·021) | p = 0·0021 | -0·220 (-0·446, 0·006) | p = 0·0007 |
|  | **De Novo (n=376)** | | | | | |
| **Imputed** | | | | | | |
| Control | -0·101 (-0·230, 0·027) | 0·023 (-0·149, 0·195) | -0·080 (-0·199, 0·039) | 0·079 (-0·073, 0·231) | -0·077 (-0·196, 0·041) | 0·082 (-0·066, 0·231) |
| Hearing Intervention | -0·078 (-0·208, 0·052) | p = 0·79 | 0·000 (-0·129, 0·128) | p = 0·31 | 0·005 (-0·120, 0·130) | p = 0·28 |
| **Complete case** | | | | | | |
| Control | -0·101 (-0·230, 0·027) | 0·023 (-0·149, 0·195) | -0·041 (-0·163, 0·081) | 0·075 (-0·079, 0·230) | -0·031 (-0·153, 0·091) | 0·078 (-0·073, 0·228) |
| Hearing Intervention | -0·078 (-0·208, 0·052) | p = 0·79 | 0·034 (-0·097, 0·166) | p = 0·34 | 0·047 (-0·081, 0·175) | p = 0·31 |

Abbreviations: ACHIEVE, Aging and Cognitive Health Evaluation in Elders Trial; ARIC, Atherosclerosis in Communities Cohort; CI, confidence interval.

Point estimates, 95% confidence intervals, and p values were calculated by fitting a single linear regression model for glial fibrillary acidic protein and neurofilament light chain. The first model adjusted for the time in years between randomisation and the follow-up assessment. The second model added baseline measures of age, sex, recruitment source, field site, education, hearing loss (pure tone average <40 dB vs ≥40 dB), and the presence of apolipoprotein E ε4 alleles. The third model integrated baseline measures of body mass index and kidney disease. Glial fibrillary acidic protein and neurofilament light chain were measured using the Alamar NUcleic acid Linked Immuno-Sandwich Assay (NULISA™) central nervous system 120 kit. Higher scores indicate greater neuroinflammation or neurodegeneration. To prevent outliers from biasing results, a rank-based inverse normal transformation was applied to each biomarker prior to statistical analysis.

# **eTable 21.** Per-Protocol Analysis **of Differences in Pre-Specified Blood-Based Biomarkers and Cognition Among Participants with Follow-Up Sample in ACHIEVE by Randomly Assigned Treatment and Recruitment Source (n=552)**

|  | **Model 1** | | **Model 2** | | **Model 3** | |
| --- | --- | --- | --- | --- | --- | --- |
|  | **Value** | **Difference** | **Value** | **Difference** | **Value** | **Difference** |
|  | **β (95% CI)** | **β (95% CI)** | **β (95% CI)** | **β (95% CI)** | **β (95% CI)** | **β (95% CI)** |
|  | **Total (n=494)** | | | | | |
| **Glial fibrillary acidic protein** | | | | | | |
| Control | 0·039 (-0·099, 0·177) | -0·075 (-0·255, 0·106) | 0·038 (-0·089, 0·165) | -0·058 (-0·229, 0·114) | 0·039 (-0·088, 0·165) | -0·055 (-0·225, 0·115) |
| Hearing Intervention | -0·035 (-0·152, 0·081) | p = 0·42 | -0·020 (-0·132, 0·093) | p = 0·51 | -0·017 (-0·127, 0·093) | p = 0·52 |
| **Neurofilament light chain** | | | | | | |
| Control | -0·023 (-0·151, 0·105) | -0·056 (-0·234, 0·123) | -0·047 (-0·164, 0·070) | -0·005 (-0·170, 0·159) | -0·052 (-0·162, 0·058) | 0·001 (-0·157, 0·159) |
| Hearing Intervention | -0·079 (-0·202, 0·045) | p = 0·54 | -0·052 (-0·166, 0·061) | p = 0·95 | -0·051 (-0·163, 0·060) | p = 0·99 |
| **Cognition** | | | | | | |
| Control | -0·249 (-0·386, -0·113) | 0·141 (-0·041, 0·324) | -0·206 (-0·320, -0·093) | 0·074 (-0·080, 0·227) | -0·202 (-0·314, -0·089) | 0·071 (-0·082, 0·224) |
| Hearing Intervention | -0·108 (-0·229, 0·013) | p = 0·13 | -0·132 (-0·232, -0·032) | p = 0·35 | -0·130 (-0·230, -0·031) | p = 0·36 |
|  | **ARIC (n=160)** | | | | | |
| **Glial fibrillary acidic protein** | | | | | | |
| Control | 0·225 (0·010, 0·439) | -0·449 (-0·758, -0·140) | 0·131 (-0·096, 0·358) | -0·438 (-0·738, -0·137) | 0·189 (-0·034, 0·411) | -0·442 (-0·725, -0·159) |
| Hearing Intervention | -0·224 (-0·459, 0·011) | p = 0·0044 | -0·307 (-0·562, -0·052) | p = 0·0043 | -0·253 (-0·501, -0·006) | p = 0·0022 |
| **Neurofilament light chain** | | | | | | |
| Control | 0·308 (0·100, 0·516) | -0·205 (-0·517, 0·107) | 0·183 (-0·032, 0·399) | -0·245 (-0·556, 0·065) | 0·247 (0·042, 0·453) | -0·277 (-0·557, 0·003) |
| Hearing Intervention | 0·103 (-0·152, 0·359) | p = 0·20 | -0·062 (-0·340, 0·216) | p = 0·12 | -0·030 (-0·290, 0·230) | p = 0·052 |
| **Cognition** | | | | | | |
| Control | -0·661 (-0·924, -0·397) | 0·195 (-0·186, 0·576) | -0·562 (-0·809, -0·314) | 0·181 (-0·129, 0·491) | -0·577 (-0·817, -0·337) | 0·201 (-0·108, 0·510) |
| Hearing Intervention | -0·466 (-0·791, -0·140) | p = 0·32 | -0·381 (-0·679, -0·083) | p = 0·25 | -0·376 (-0·673, -0·079) | p = 0·20 |
|  | **De Novo (n=334)** | | | | | |
| **Glial fibrillary acidic protein** | | | | | | |
| Control | -0·070 (-0·255, 0·115) | 0·110 (-0·113, 0·333) | -0·018 (-0·196, 0·159) | 0·123 (-0·081, 0·327) | -0·022 (-0·201, 0·157) | 0·131 (-0·072, 0·334) |
| Hearing Intervention | 0·040 (-0·105, 0·185) | p = 0·34 | 0·105 (-0·056, 0·266) | p = 0·24 | 0·109 (-0·049, 0·268) | p = 0·21 |
| **Neurofilament light chain** | | | | | | |
| Control | -0·293 (-0·451, -0·135) | 0·092 (-0·118, 0·302) | -0·227 (-0·372, -0·082) | 0·113 (-0·077, 0·302) | -0·236 (-0·375, -0·097) | 0·130 (-0·056, 0·316) |
| Hearing Intervention | -0·201 (-0·352, -0·049) | p = 0·39 | -0·115 (-0·250, 0·020) | p = 0·25 | -0·106 (-0·240, 0·028) | p = 0·17 |
| **Cognition** | | | | | | |
| Control | 0·003 (-0·157, 0·162) | 0·021 (-0·173, 0·214) | -0·057 (-0·197, 0·084) | 0·015 (-0·154, 0·184) | -0·053 (-0·194, 0·087) | 0·010 (-0·159, 0·180) |
| Hearing Intervention | 0·023 (-0·110, 0·157) | p = 0·83 | -0·042 (-0·156, 0·072) | p = 0·86 | -0·043 (-0·157, 0·071) | p = 0·90 |

Abbreviations: ACHIEVE, Aging and Cognitive Health Evaluation in Elders Trial; ARIC, Atherosclerosis in Communities Cohort; CI, confidence interval.

Point estimates, 95% confidence intervals, and p values were calculated by fitting separate linear regression models for each outcome to imputed data. The first model adjusted for the time in years between randomisation and the follow-up assessment. The second model added baseline measures of age, sex, recruitment source, field site, education, hearing loss (pure tone average <40 dB vs ≥40 dB), and the presence of apolipoprotein E ε4 alleles. The third model integrated baseline measures of body mass index and kidney disease. Glial fibrillary acidic protein and neurofilament light chain were measured using the Alamar NUcleic acid Linked Immuno-Sandwich Assay (NULISA™) central nervous system 120 kit. Higher scores indicate greater neuroinflammation or neurodegeneration. To prevent outliers from biasing results, a rank-based inverse normal transformation was applied to each biomarker prior to statistical analysis. A factor score quantifying cognition was developed using a validated latent variable modelling approach and standardised to the baseline, with lower scores indicating worse cognitive function.

# **eTable 22.** Complier Average Causal Effect Analysis of **Differences in Pre-Specified Blood-Based Biomarkers and Cognition Among Participants with Follow-Up Samples in ACHIEVE by Randomly Assigned Treatment and Recruitment Source (n=552)**

|  | **Model 1** | | **Model 2** | | **Model 3** | |
| --- | --- | --- | --- | --- | --- | --- |
|  | **Value** | **Difference** | **Value** | **Difference** | **Value** | **Difference** |
|  | **β (95% CI)** | **β (95% CI)** | **β (95% CI)** | **β (95% CI)** | **β (95% CI)** | **β (95% CI)** |
|  | **Total (n=552)** | | | | | |
| **Glial fibrillary acidic protein** | | | | | | |
| Control | 0·081 (-0·048, 0·209) | -0·156 (-0·338, 0·026) | 0·057 (-0·058, 0·171) | -0·100 (-0·272, 0·071) | 0·048 (-0·066, 0·163) | -0·092 (-0·261, 0·078) |
| Hearing Intervention | -0·075 (-0·203, 0·052) | p = 0·093 | -0·044 (-0·165, 0·077) | p = 0·25 | -0·043 (-0·161, 0·075) | p = 0·29 |
| **Neurofilament light chain** | | | | | | |
| Control | 0·093 (-0·045, 0·230) | -0·194 (-0·388, -0·001) | 0·032 (-0·076, 0·140) | -0·099 (-0·270, 0·071) | 0·018 (-0·083, 0·118) | -0·088 (-0·250, 0·073) |
| Hearing Intervention | -0·102 (-0·235, 0·032) | p = 0·049 | -0·067 (-0·197, 0·062) | p = 0·25 | -0·071 (-0·193, 0·052) | p = 0·28 |
| **Cognition** | | | | | | |
| Control | -0·381 (-0·533, -0·229) | 0·189 (-0·011, 0·389) | -0·272 (-0·382, -0·161) | 0·088 (-0·062, 0·239) | -0·270 (-0·379, -0·162) | 0·090 (-0·060, 0·240) |
| Hearing Intervention | -0·192 (-0·323, -0·061) | p = 0·064 | -0·183 (-0·284, -0·083) | p = 0·25 | -0·180 (-0·281, -0·080) | p = 0·24 |
|  | **ARIC (n=176)** | | | | | |
| **Glial fibrillary acidic protein** | | | | | | |
| Control | 0·295 (0·066, 0·523) | -0·553 (-0·891, -0·215) | 0·134 (-0·074, 0·342) | -0·495 (-0·799, -0·191) | 0·146 (-0·059, 0·352) | -0·459 (-0·750, -0·168) |
| Hearing Intervention | -0·259 (-0·510, -0·008) | p = 0·0013 | -0·361 (-0·612, -0·111) | p = 0·0014 | -0·313 (-0·557, -0·068) | p = 0·0020 |
| **Neurofilament light chain** | | | | | | |
| Control | 0·463 (0·254, 0·673) | -0·350 (-0·661, -0·039) | 0·312 (0·103, 0·520) | -0·369 (-0·665, -0·073) | 0·332 (0·144, 0·520) | -0·364 (-0·635, -0·093) |
| Hearing Intervention | 0·114 (-0·136, 0·363) | p = 0·028 | -0·058 (-0·323, 0·207) | p = 0·015 | -0·033 (-0·282, 0·217) | p = 0·0084 |
| **Cognition** | | | | | | |
| Control | -0·791 (-1·064, -0·519) | 0·129 (-0·288, 0·546) | -0·626 (-0·897, -0·355) | 0·230 (-0·089, 0·550) | -0·628 (-0·892, -0·363) | 0·244 (-0·082, 0·569) |
| Hearing Intervention | -0·662 (-1·011, -0·313) | p = 0·54 | -0·395 (-0·680, -0·111) | p = 0·16 | -0·384 (-0·670, -0·098) | p = 0·14 |
|  | **De Novo (n=376)** | | | | | |
| **Glial fibrillary acidic protein** | | | | | | |
| Control | -0·007 (-0·174, 0·159) | 0·056 (-0·154, 0·266) | 0·002 (-0·156, 0·160) | 0·108 (-0·081, 0·297) | -0·002 (-0·162, 0·158) | 0·111 (-0·077, 0·298) |
| Hearing Intervention | 0·048 (-0·099, 0·196) | p = 0·60 | 0·110 (-0·045, 0·265) | p = 0·26 | 0·109 (-0·042, 0·259) | p = 0·25 |
| **Neurofilament light chain** | | | | | | |
| Control | -0·188 (-0·327, -0·049) | 0·021 (-0·174, 0·216) | -0·152 (-0·280, -0·023) | 0·069 (-0·111, 0·248) | -0·159 (-0·283, -0·034) | 0·073 (-0·100, 0·247) |
| Hearing Intervention | -0·167 (-0·317, -0·018) | p = 0·83 | -0·083 (-0·220, 0·054) | p = 0·45 | -0·085 (-0·217, 0·047) | p = 0·41 |
| **Cognition** | | | | | | |
| Control | -0·064 (-0·208, 0·079) | 0·079 (-0·107, 0·265) | -0·105 (-0·231, 0·021) | 0·041 (-0·122, 0·204) | -0·103 (-0·230, 0·024) | 0·040 (-0·123, 0·203) |
| Hearing Intervention | 0·014 (-0·124, 0·152) | p = 0·41 | -0·064 (-0·182, 0·055) | p = 0·62 | -0·063 (-0·179, 0·053) | p = 0·63 |

Abbreviations: ACHIEVE, Aging and Cognitive Health Evaluation in Elders Trial; ARIC, Atherosclerosis in Communities Cohort; CI, confidence interval.

Point estimates, 95% confidence intervals, and p values were calculated by fitting separate weighted linear regression models for each outcome to imputed data. Weights estimated the probability of complying with the intervention protocol if assigned to treatment. The first model adjusted for the time in years between randomisation and the follow-up assessment. The second model added baseline measures of age, sex, recruitment source, field site, education, hearing loss (pure tone average <40 dB vs ≥40 dB), and the presence of apolipoprotein E ε4 alleles. The third model integrated baseline measures of body mass index and kidney disease. Glial fibrillary acidic protein and neurofilament light chain were measured using the Alamar NUcleic acid Linked Immuno-Sandwich Assay (NULISA™) central nervous system 120 kit. Higher scores indicate greater neuroinflammation or neurodegeneration. To prevent outliers from biasing results, a rank-based inverse normal transformation was applied to each biomarker prior to statistical analysis. A factor score quantifying cognition was developed using a validated latent variable modelling approach and standardised to the baseline, with lower scores indicating worse cognitive function.

# **eTable 23. Selection Weighted Intention-to-Treat Analysis of Differences in Pre-Specified Blood-Based Biomarkers and Cognition Among Participants with Follow-Up Samples in ACHIEVE by Randomly Assigned Treatment and Recruitment Source (n=552)**

|  | **Model 1** | | **Model 2** | | **Model 3** | |
| --- | --- | --- | --- | --- | --- | --- |
|  | **Value** | **Difference** | **Value** | **Difference** | **Value** | **Difference** |
|  | **β (95% CI)** | **β (95% CI)** | **β (95% CI)** | **β (95% CI)** | **β (95% CI)** | **β (95% CI)** |
|  | **Total (n=552)** | | | | | |
| **Glial fibrillary acidic protein** | | | | | | |
| Control | 0·119 (-0·028, 0·267) | -0·124 (-0·315, 0·066) | 0·097 (-0·035, 0·229) | -0·139 (-0·313, 0·036) | 0·103 (-0·030, 0·236) | -0·142 (-0·317, 0·032) |
| Hearing Intervention | -0·005 (-0·126, 0·116) | p = 0·20 | -0·042 (-0·153, 0·069) | p = 0·12 | -0·039 (-0·147, 0·069) | p = 0·11 |
| **Neurofilament light chain** | | | | | | |
| Control | -0·002 (-0·136, 0·133) | -0·030 (-0·220, 0·161) | -0·004 (-0·119, 0·111) | -0·022 (-0·191, 0·146) | 0·002 (-0·107, 0·111) | -0·028 (-0·190, 0·134) |
| Hearing Intervention | -0·032 (-0·165, 0·102) | p = 0·76 | -0·027 (-0·150, 0·096) | p = 0·79 | -0·026 (-0·144, 0·093) | p = 0·74 |
| **Cognition** | | | | | | |
| Control | -0·303 (-0·445, -0·160) | 0·159 (-0·034, 0·352) | -0·310 (-0·432, -0·187) | 0·094 (-0·077, 0·266) | -0·307 (-0·426, -0·188) | 0·094 (-0·075, 0·264) |
| Hearing Intervention | -0·144 (-0·271, -0·016) | p = 0·11 | -0·215 (-0·330, -0·101) | p = 0·28 | -0·213 (-0·327, -0·099) | p = 0·28 |
|  | **ARIC (n=176)** | | | | | |
| **Glial fibrillary acidic protein** | | | | | | |
| Control | 0·222 (0·027, 0·418) | -0·481 (-0·780, -0·183) | 0·127 (-0·083, 0·337) | -0·467 (-0·758, -0·176) | 0·166 (-0·052, 0·384) | -0·496 (-0·786, -0·206) |
| Hearing Intervention | -0·259 (-0·500, -0·019) | p = 0·0016 | -0·340 (-0·589, -0·092) | p = 0·0017 | -0·330 (-0·571, -0·089) | p = 0·0008 |
| **Neurofilament light chain** | | | | | | |
| Control | 0·327 (0·031, 0·622) | -0·015 (-0·528, 0·499) | 0·189 (-0·084, 0·461) | -0·137 (-0·527, 0·254) | 0·246 (0·034, 0·457) | -0·288 (-0·584, 0·008) |
| Hearing Intervention | 0·312 (-0·151, 0·775) | p = 0·96 | 0·052 (-0·274, 0·378) | p = 0·49 | -0·043 (-0·308, 0·222) | p = 0·057 |
| **Cognition** | | | | | | |
| Control | -0·930 (-1·343, -0·517) | 0·017 (-0·917, 0·950) | -0·660 (-0·931, -0·390) | 0·192 (-0·152, 0·536) | -0·661 (-0·916, -0·406) | 0·293 (-0·035, 0·621) |
| Hearing Intervention | -0·913 (-1·782, -0·045) | p = 0·97 | -0·468 (-0·769, -0·168) | p = 0·27 | -0·368 (-0·659, -0·077) | p = 0·080 |
|  | **De Novo (n=376)** | | | | | |
| **Glial fibrillary acidic protein** | | | | | | |
| Control | 0·094 (-0·102, 0·290) | -0·032 (-0·274, 0·210) | 0·091 (-0·115, 0·296) | 0·002 (-0·226, 0·231) | 0·099 (-0·106, 0·305) | 0·003 (-0·223, 0·228) |
| Hearing Intervention | 0·062 (-0·095, 0·219) | p = 0·80 | 0·093 (-0·061, 0·247) | p = 0·99 | 0·102 (-0·048, 0·252) | p = 0·98 |
| **Neurofilament light chain** | | | | | | |
| Control | -0·186 (-0·354, -0·018) | 0·081 (-0·148, 0·311) | -0·153 (-0·309, 0·002) | 0·094 (-0·111, 0·300) | -0·138 (-0·292, 0·017) | 0·095 (-0·105, 0·296) |
| Hearing Intervention | -0·105 (-0·279, 0·069) | p = 0·49 | -0·059 (-0·213, 0·096) | p = 0·37 | -0·042 (-0·192, 0·107) | p = 0·35 |
| **Cognition** | | | | | | |
| Control | -0·116 (-0·266, 0·034) | 0·075 (-0·131, 0·281) | -0·142 (-0·279, -0·004) | 0·030 (-0·152, 0·212) | -0·139 (-0·278, 0·000) | 0·028 (-0·156, 0·211) |
| Hearing Intervention | -0·041 (-0·202, 0·120) | p = 0·48 | -0·111 (-0·242, 0·020) | p = 0·75 | -0·111 (-0·241, 0·019) | p = 0·77 |

Abbreviations: ACHIEVE, Aging and Cognitive Health Evaluation in Elders Trial; ARIC, Atherosclerosis in Communities Cohort; CI, confidence interval.

Point estimates, 95% confidence intervals, and p values were calculated by fitting separate weighted linear regression models for each outcome to imputed data. Weights estimated the probability of having an analysed sample. The first model adjusted for the time in years between randomisation and the follow-up assessment. The second model added baseline measures of age, sex, recruitment source, field site, education, hearing loss (pure tone average <40 dB vs ≥40 dB), and the presence of apolipoprotein E ε4 alleles. The third model integrated baseline measures of body mass index and kidney disease. Glial fibrillary acidic protein and neurofilament light chain were measured using the Alamar NUcleic acid Linked Immuno-Sandwich Assay (NULISA™) central nervous system 120 kit. Higher scores indicate greater neuroinflammation or neurodegeneration. To prevent outliers from biasing results, a rank-based inverse normal transformation was applied to each biomarker prior to statistical analysis. A factor score quantifying cognition was developed using a validated latent variable modelling approach and standardised to the baseline, with lower scores indicating worse cognitive function.

# **eTable 24. Intention-to-Treat Analysis of Differences in Exploratory Blood-Based Biomarkers of Alzheimer's Disease Pathology Among Participants with Follow-Up Samples in ACHIEVE by Randomly Assigned Treatment and Recruitment Source (n=552)**

|  | **Model 1** | | **Model 2** | | **Model 3** | |
| --- | --- | --- | --- | --- | --- | --- |
|  | **Value** | **Difference** | **Value** | **Difference** | **Value** | **Difference** |
|  | **β (95% CI)** | **β (95% CI)** | **β (95% CI)** | **β (95% CI)** | **β (95% CI)** | **β (95% CI)** |
|  | **Total (n=552)** | | | | | |
| **Phosphorylated tau 181** | | | | | | |
| Control | 0·063 (-0·058, 0·185) | -0·080 (-0·249, 0·090) | 0·066 (-0·052, 0·184) | -0·086 (-0·251, 0·080) | 0·069 (-0·047, 0·185) | -0·091 (-0·253, 0·072) |
| Hearing Intervention | -0·016 (-0·134, 0·101) | p = 0·36 | -0·019 (-0·134, 0·095) | p = 0·31 | -0·022 (-0·133, 0·090) | p = 0·28 |
| **Phosphorylated tau 217** | | | | | | |
| Control | 0·064 (-0·058, 0·186) | -0·107 (-0·276, 0·062) | 0·067 (-0·053, 0·186) | -0·112 (-0·278, 0·054) | 0·068 (-0·051, 0·187) | -0·114 (-0·279, 0·050) |
| Hearing Intervention | -0·043 (-0·160, 0·074) | p = 0·21 | -0·045 (-0·159, 0·068) | p = 0·19 | -0·046 (-0·158, 0·065) | p = 0·17 |
| **Phosphorylated tau 231** | | | | | | |
| Control | 0·104 (-0·017, 0·225) | -0·138 (-0·308, 0·031) | 0·107 (-0·011, 0·225) | -0·144 (-0·310, 0·022) | 0·110 (-0·007, 0·226) | -0·148 (-0·311, 0·015) |
| Hearing Intervention | -0·034 (-0·153, 0·084) | p = 0·11 | -0·037 (-0·152, 0·078) | p = 0·089 | -0·039 (-0·152, 0·074) | p = 0·075 |
|  | **ARIC (n=176)** | | | | | |
| **Phosphorylated tau 181** | | | | | | |
| Control | 0·185 (-0·006, 0·375) | -0·338 (-0·632, -0·045) | 0·141 (-0·052, 0·333) | -0·366 (-0·656, -0·077) | 0·152 (-0·037, 0·341) | -0·384 (-0·667, -0·101) |
| Hearing Intervention | -0·154 (-0·398, 0·091) | p = 0·024 | -0·226 (-0·486, 0·035) | p = 0·013 | -0·232 (-0·487, 0·024) | p = 0·0079 |
| **Phosphorylated tau 217** | | | | | | |
| Control | 0·240 (0·053, 0·428) | -0·510 (-0·783, -0·238) | 0·142 (-0·048, 0·332) | -0·498 (-0·763, -0·233) | 0·149 (-0·043, 0·340) | -0·502 (-0·762, -0·243) |
| Hearing Intervention | -0·270 (-0·489, -0·051) | p = 0·0002 | -0·356 (-0·597, -0·115) | p = 0·0002 | -0·354 (-0·589, -0·118) | p = 0·0001 |
| **Phosphorylated tau 231** | | | | | | |
| Control | 0·215 (0·021, 0·409) | -0·472 (-0·763, -0·180) | 0·174 (-0·021, 0·368) | -0·487 (-0·777, -0·197) | 0·185 (-0·005, 0·375) | -0·506 (-0·788, -0·224) |
| Hearing Intervention | -0·257 (-0·498, -0·015) | p = 0·0015 | -0·313 (-0·577, -0·049) | p = 0·0010 | -0·321 (-0·581, -0·061) | p = 0·0004 |
|  | **De Novo (n=376)** | | | | | |
| **Phosphorylated tau 181** | | | | | | |
| Control | -0·041 (-0·206, 0·123) | 0·047 (-0·163, 0·257) | -0·039 (-0·195, 0·116) | 0·038 (-0·164, 0·240) | -0·033 (-0·186, 0·120) | 0·044 (-0·154, 0·242) |
| Hearing Intervention | 0·006 (-0·132, 0·143) | p = 0·66 | -0·001 (-0·132, 0·131) | p = 0·71 | 0·011 (-0·118, 0·140) | p = 0·66 |
| **Phosphorylated tau 217** | | | | | | |
| Control | -0·106 (-0·273, 0·060) | 0·090 (-0·121, 0·302) | -0·087 (-0·247, 0·074) | 0·084 (-0·120, 0·288) | -0·082 (-0·241, 0·076) | 0·088 (-0·112, 0·289) |
| Hearing Intervention | -0·016 (-0·158, 0·126) | p = 0·40 | -0·003 (-0·140, 0·134) | p = 0·42 | 0·006 (-0·129, 0·140) | p = 0·39 |
| **Phosphorylated tau 231** | | | | | | |
| Control | -0·021 (-0·182, 0·139) | 0·021 (-0·188, 0·229) | -0·014 (-0·168, 0·140) | 0·021 (-0·180, 0·222) | -0·008 (-0·160, 0·143) | 0·026 (-0·171, 0·224) |
| Hearing Intervention | 0·000 (-0·141, 0·140) | p = 0·85 | 0·007 (-0·127, 0·142) | p = 0·84 | 0·018 (-0·114, 0·150) | p = 0·79 |

Abbreviations: ACHIEVE, Aging and Cognitive Health Evaluation in Elders Trial; ARIC, Atherosclerosis in Communities Cohort; CI, confidence interval.

Point estimates, 95% confidence intervals, and p values were calculated by fitting separate linear regression models for each outcome to imputed data. The first model adjusted for the time in years between randomisation and the follow-up assessment. The second model added baseline measures of age, sex, recruitment source, field site, education, hearing loss (pure tone average <40 dB vs ≥40 dB), and the presence of apolipoprotein E ε4 alleles. The third model integrated baseline measures of body mass index and kidney disease. Phosphorylated tau at threonine 181, 217, and 231 was measured using the Alamar NUcleic acid Linked Immuno-Sandwich Assay (NULISA™) central nervous system 120 kit. Higher scores indicate greater Alzheimer's disease pathology. To prevent outliers from biasing results, a rank-based inverse normal transformation was applied to each biomarker prior to statistical analysis.

# **eTable 25. Complete Case Intention-to-Treat Analysis of Differences in Exploratory Blood-Based Biomarkers of Alzheimer's Disease Pathology Among Participants with Follow-Up Samples in ACHIEVE by Randomly Assigned Treatment and Recruitment Source (n=552)**

|  | **Model 1** | | **Model 2** | | **Model 3** | |
| --- | --- | --- | --- | --- | --- | --- |
|  | **Value** | **Difference** | **Value** | **Difference** | **Value** | **Difference** |
|  | **β (95% CI)** | **β (95% CI)** | **β (95% CI)** | **β (95% CI)** | **β (95% CI)** | **β (95% CI)** |
|  | **Total (n=552)** | | | | | |
| **Phosphorylated tau 181** | | | | | | |
| Control | 0·063 (-0·058, 0·185) | -0·072 (-0·242, 0·098) | 0·072 (-0·049, 0·194) | -0·048 (-0·220, 0·123) | 0·085 (-0·035, 0·205) | -0·059 (-0·227, 0·110) |
| Hearing Intervention | -0·009 (-0·127, 0·110) | p = 0·41 | 0·024 (-0·093, 0·141) | p = 0·58 | 0·027 (-0·088, 0·142) | p = 0·50 |
| **Phosphorylated tau 217** | | | | | | |
| Control | 0·064 (-0·058, 0·185) | -0·094 (-0·263, 0·076) | 0·074 (-0·049, 0·198) | -0·064 (-0·236, 0·108) | 0·080 (-0·043, 0·203) | -0·071 (-0·241, 0·100) |
| Hearing Intervention | -0·030 (-0·148, 0·088) | p = 0·28 | 0·010 (-0·107, 0·127) | p = 0·46 | 0·009 (-0·107, 0·125) | p = 0·42 |
| **Phosphorylated tau 231** | | | | | | |
| Control | 0·104 (-0·017, 0·224) | -0·126 (-0·296, 0·044) | 0·108 (-0·014, 0·230) | -0·089 (-0·260, 0·082) | 0·119 (0·000, 0·239) | -0·099 (-0·267, 0·069) |
| Hearing Intervention | -0·022 (-0·142, 0·097) | p = 0·15 | 0·019 (-0·099, 0·137) | p = 0·31 | 0·020 (-0·096, 0·136) | p = 0·25 |
|  | **ARIC (n=176)** | | | | | |
| **Phosphorylated tau 181** | | | | | | |
| Control | 0·187 (-0·005, 0·378) | -0·316 (-0·615, -0·017) | 0·190 (-0·009, 0·389) | -0·302 (-0·610, 0·006) | 0·196 (0·000, 0·391) | -0·327 (-0·634, -0·019) |
| Hearing Intervention | -0·129 (-0·381, 0·122) | p = 0·038 | -0·112 (-0·388, 0·165) | p = 0·054 | -0·131 (-0·406, 0·144) | p = 0·037 |
| **Phosphorylated tau 217** | | | | | | |
| Control | 0·239 (0·052, 0·426) | -0·470 (-0·745, -0·194) | 0·181 (-0·017, 0·379) | -0·423 (-0·704, -0·141) | 0·182 (-0·017, 0·381) | -0·432 (-0·706, -0·157) |
| Hearing Intervention | -0·231 (-0·453, -0·008) | p = 0·0008 | -0·242 (-0·505, 0·022) | p = 0·0032 | -0·250 (-0·507, 0·008) | p = 0·002 |
| **Phosphorylated tau 231** | | | | | | |
| Control | 0·216 (0·022, 0·411) | -0·435 (-0·730, -0·140) | 0·223 (0·022, 0·424) | -0·380 (-0·684, -0·076) | 0·228 (0·033, 0·424) | -0·409 (-0·710, -0·107) |
| Hearing Intervention | -0·219 (-0·465, 0·027) | p = 0·0039 | -0·157 (-0·432, 0·117) | p = 0·014 | -0·180 (-0·454, 0·094) | p = 0·0079 |
|  | **De Novo (n=376)** | | | | | |
| **Phosphorylated tau 181** | | | | | | |
| Control | -0·041 (-0·206, 0·123) | 0·047 (-0·163, 0·257) | -0·039 (-0·198, 0·120) | 0·045 (-0·161, 0·251) | -0·017 (-0·175, 0·140) | 0·047 (-0·154, 0·249) |
| Hearing Intervention | 0·006 (-0·132, 0·143) | p = 0·66 | 0·006 (-0·127, 0·140) | p = 0·66 | 0·030 (-0·102, 0·162) | p = 0·65 |
| **Phosphorylated tau 217** | | | | | | |
| Control | -0·106 (-0·273, 0·060) | 0·090 (-0·121, 0·302) | -0·087 (-0·251, 0·077) | 0·098 (-0·110, 0·306) | -0·073 (-0·236, 0·091) | 0·097 (-0·108, 0·303) |
| Hearing Intervention | -0·016 (-0·158, 0·126) | p = 0·40 | 0·011 (-0·128, 0·151) | p = 0·35 | 0·025 (-0·113, 0·163) | p = 0·35 |
| **Phosphorylated tau 231** | | | | | | |
| Control | -0·021 (-0·182, 0·139) | 0·021 (-0·188, 0·229) | -0·021 (-0·179, 0·137) | 0·030 (-0·176, 0·236) | -0·001 (-0·156, 0·154) | 0·031 (-0·171, 0·233) |
| Hearing Intervention | 0·000 (-0·141, 0·140) | p = 0·85 | 0·009 (-0·128, 0·146) | p = 0·78 | 0·030 (-0·106, 0·166) | p = 0·76 |

Abbreviations: ACHIEVE, Aging and Cognitive Health Evaluation in Elders Trial; ARIC, Atherosclerosis in Communities Cohort; CI, confidence interval.

Point estimates, 95% confidence intervals, and p values were calculated by fitting separate linear regression models for each outcome to complete case data. The first model adjusted for the time in years between randomisation and the follow-up assessment. The second model added baseline measures of age, sex, recruitment source, field site, education, hearing loss (pure tone average <40 dB vs ≥40 dB), and the presence of apolipoprotein E ε4 alleles. The third model integrated baseline measures of body mass index and kidney disease. Phosphorylated tau at threonine 181, 217, and 231 was measured using the Alamar NUcleic acid Linked Immuno-Sandwich Assay (NULISA™) central nervous system 120 kit. Higher scores indicate greater Alzheimer's disease pathology. To prevent outliers from biasing results, a rank-based inverse normal transformation was applied to each biomarker prior to statistical analysis.

# **eTable 26. Intention-to-Treat Analysis of Differences in Untransformed Exploratory Blood-Based Biomarkers of Alzheimer's Disease Pathology Among Participants with Follow-Up Samples in ACHIEVE by Randomly Assigned Treatment and Recruitment Source (n=552)**

|  | **Model 1** | | **Model 2** | | **Model 3** | |
| --- | --- | --- | --- | --- | --- | --- |
|  | **NPQ units** | **Difference** | **NPQ units** | **Difference** | **NPQ units** | **Difference** |
|  | **β (95% CI)** | **β (95% CI)** | **β (95% CI)** | **β (95% CI)** | **β (95% CI)** | **β (95% CI)** |
|  | **Total (n=552)** | | | | | |
| **Phosphorylated tau 181** | | | | | | |
| Control | 13·375 (13·301, 13·449) | -0·041 (-0·145, 0·063) | 13·376 (13·304, 13·448) | -0·044 (-0·146, 0·058) | 13·378 (13·308, 13·448) | -0·048 (-0·147, 0·052) |
| Hearing Intervention | 13·333 (13·261, 13·406) | p = 0·44 | 13·332 (13·261, 13·403) | p = 0·39 | 13·330 (13·261, 13·400) | p = 0·35 |
| **Phosphorylated tau 217** | | | | | | |
| Control | 11·630 (11·564, 11·696) | -0·055 (-0·147, 0·037) | 11·632 (11·567, 11·698) | -0·059 (-0·149, 0·031) | 11·633 (11·568, 11·698) | -0·060 (-0·149, 0·028) |
| Hearing Intervention | 11·575 (11·512, 11·639) | p = 0·24 | 11·574 (11·512, 11·635) | p = 0·20 | 11·573 (11·513, 11·633) | p = 0·18 |
| **Phosphorylated tau 231** | | | | | | |
| Control | 13·649 (13·568, 13·730) | -0·085 (-0·198, 0·029) | 13·651 (13·572, 13·730) | -0·089 (-0·199, 0·022) | 13·653 (13·576, 13·730) | -0·092 (-0·200, 0·016) |
| Hearing Intervention | 13·564 (13·485, 13·644) | p = 0·14 | 13·563 (13·486, 13·640) | p = 0·12 | 13·561 (13·486, 13·636) | p = 0·096 |
|  | **ARIC (n=176)** | | | | | |
| **Phosphorylated tau 181** | | | | | | |
| Control | 13·440 (13·319, 13·561) | -0·185 (-0·365, -0·006) | 13·413 (13·294, 13·531) | -0·201 (-0·379, -0·023) | 13·421 (13·305, 13·537) | -0·215 (-0·387, -0·043) |
| Hearing Intervention | 13·255 (13·108, 13·402) | p = 0·043 | 13·212 (13·054, 13·370) | p = 0·027 | 13·206 (13·053, 13·358) | p = 0·014 |
| **Phosphorylated tau 217** | | | | | | |
| Control | 11·703 (11·593, 11·814) | -0·262 (-0·414, -0·110) | 11·653 (11·544, 11·761) | -0·251 (-0·398, -0·104) | 11·657 (11·547, 11·767) | -0·255 (-0·398, -0·112) |
| Hearing Intervention | 11·441 (11·329, 11·553) | p = 0·0007 | 11·402 (11·276, 11·528) | p = 0·0008 | 11·402 (11·281, 11·523) | p = 0·0005 |
| **Phosphorylated tau 231** | | | | | | |
| Control | 13·714 (13·579, 13·848) | -0·297 (-0·487, -0·107) | 13·682 (13·551, 13·813) | -0·304 (-0·493, -0·114) | 13·690 (13·562, 13·818) | -0·319 (-0·502, -0·136) |
| Hearing Intervention | 13·417 (13·266, 13·567) | p = 0·0022 | 13·378 (13·211, 13·545) | p = 0·0017 | 13·371 (13·208, 13·534) | p = 0·0006 |
|  | **De Novo (n=376)** | | | | | |
| **Phosphorylated tau 181** | | | | | | |
| Control | 13·311 (13·213, 13·409) | 0·030 (-0·099, 0·158) | 13·311 (13·218, 13·404) | 0·025 (-0·099, 0·149) | 13·315 (13·223, 13·406) | 0·029 (-0·093, 0·150) |
| Hearing Intervention | 13·341 (13·258, 13·424) | p = 0·65 | 13·336 (13·255, 13·416) | p = 0·69 | 13·343 (13·264, 13·422) | p = 0·64 |
| **Phosphorylated tau 217** | | | | | | |
| Control | 11·543 (11·456, 11·629) | 0·046 (-0·067, 0·159) | 11·554 (11·469, 11·638) | 0·040 (-0·070, 0·150) | 11·556 (11·473, 11·640) | 0·043 (-0·064, 0·150) |
| Hearing Intervention | 11·589 (11·512, 11·665) | p = 0·43 | 11·594 (11·520, 11·669) | p = 0·47 | 11·599 (11·526, 11·673) | p = 0·43 |
| **Phosphorylated tau 231** | | | | | | |
| Control | 13·566 (13·462, 13·670) | 0·017 (-0·123, 0·156) | 13·570 (13·469, 13·670) | 0·015 (-0·120, 0·150) | 13·574 (13·475, 13·672) | 0·019 (-0·112, 0·151) |
| Hearing Intervention | 13·582 (13·489, 13·676) | p = 0·81 | 13·585 (13·495, 13·675) | p = 0·82 | 13·593 (13·504, 13·681) | p = 0·77 |

Abbreviations: ACHIEVE, Aging and Cognitive Health Evaluation in Elders Trial; ARIC, Atherosclerosis in Communities Cohort; CI, confidence interval; NULISA protein quantification, NPQ.

Point estimates, 95% confidence intervals, and p values were calculated by fitting separate linear regression models for each outcome to imputed data. The first model adjusted for the time in years between randomisation and the follow-up assessment. The second model added baseline measures of age, sex, recruitment source, field site, education, hearing loss (pure tone average <40 dB vs ≥40 dB), and the presence of apolipoprotein E ε4 alleles. The third model integrated baseline measures of body mass index and kidney disease. Phosphorylated tau at threonine 181, 217, and 231 was measured using the Alamar NUcleic acid Linked Immuno-Sandwich Assay (NULISA™) central nervous system 120 kit, which produces sample-specific NPQ units. Higher scores indicate greater Alzheimer's disease pathology. A 1-unit change corresponds to a 2-fold increase in the concentration of the biomarker. A 2-unit change corresponds to a 4-fold increase in the concentration. Concentrations are biomarker-specific and cannot be compared to each other.

# **eTable 27. Complete Case Intention-to-Treat Analysis of Differences in Untransformed Exploratory Blood-Based Biomarkers of Alzheimer's Disease Pathology Among Participants with Follow-Up Samples in ACHIEVE by Randomly Assigned Treatment and Recruitment Source (n=552)**

|  | **Model 1** | | **Model 2** | | **Model 3** | |
| --- | --- | --- | --- | --- | --- | --- |
|  | **NPQ units** | **Difference** | **NPQ units** | **Difference** | **NPQ units** | **Difference** |
|  | **β (95% CI)** | **β (95% CI)** | **β (95% CI)** | **β (95% CI)** | **β (95% CI)** | **β (95% CI)** |
|  | **Total (n=552)** | | | | | |
| **Phosphorylated tau 181** | | | | | | |
| Control | 13·375 (13·301, 13·449) | -0·037 (-0·141, 0·068) | 13·380 (13·306, 13·454) | -0·026 (-0·132, 0·080) | 13·388 (13·315, 13·461) | -0·033 (-0·136, 0·070) |
| Hearing Intervention | 13·338 (13·265, 13·411) | p = 0·49 | 13·354 (13·281, 13·427) | p = 0·63 | 13·356 (13·285, 13·427) | p = 0·53 |
| **Phosphorylated tau 217** | | | | | | |
| Control | 11·630 (11·564, 11·696) | -0·048 (-0·140, 0·044) | 11·638 (11·571, 11·706) | -0·038 (-0·131, 0·056) | 11·642 (11·574, 11·709) | -0·041 (-0·134, 0·051) |
| Hearing Intervention | 11·582 (11·518, 11·646) | p = 0·31 | 11·601 (11·536, 11·665) | p = 0·43 | 11·600 (11·537, 11·664) | p = 0·38 |
| **Phosphorylated tau 231** | | | | | | |
| Control | 13·649 (13·568, 13·729) | -0·077 (-0·190, 0·037) | 13·653 (13·571, 13·735) | -0·057 (-0·172, 0·057) | 13·661 (13·581, 13·741) | -0·064 (-0·176, 0·047) |
| Hearing Intervention | 13·572 (13·492, 13·652) | p = 0·18 | 13·596 (13·517, 13·675) | p = 0·33 | 13·597 (13·519, 13·675) | p = 0·26 |
|  | **ARIC (n=176)** | | | | | |
| **Phosphorylated tau 181** | | | | | | |
| Control | 13·441 (13·320, 13·562) | -0·172 (-0·355, 0·012) | 13·443 (13·319, 13·566) | -0·169 (-0·357, 0·020) | 13·447 (13·328, 13·567) | -0·187 (-0·374, 0·000) |
| Hearing Intervention | 13·269 (13·118, 13·421) | p = 0·067 | 13·274 (13·104, 13·444) | p = 0·080 | 13·260 (13·094, 13·426) | p = 0·050 |
| **Phosphorylated tau 217** | | | | | | |
| Control | 11·702 (11·592, 11·812) | -0·243 (-0·397, -0·089) | 11·677 (11·565, 11·789) | -0·225 (-0·381, -0·068) | 11·678 (11·565, 11·791) | -0·231 (-0·383, -0·079) |
| Hearing Intervention | 11·459 (11·344, 11·575) | p = 0·0020 | 11·452 (11·313, 11·592) | p = 0·0049 | 11·447 (11·313, 11·582) | p = 0·0029 |
| **Phosphorylated tau 231** | | | | | | |
| Control | 13·714 (13·580, 13·848) | -0·274 (-0·467, -0·082) | 13·715 (13·580, 13·851) | -0·246 (-0·444, -0·047) | 13·720 (13·589, 13·851) | -0·267 (-0·463, -0·071) |
| Hearing Intervention | 13·440 (13·286, 13·593) | p = 0·0053 | 13·470 (13·293, 13·646) | p = 0·015 | 13·453 (13·279, 13·627) | p = 0·0077 |
|  | **De Novo (n=376)** | | | | | |
| **Phosphorylated tau 181** | | | | | | |
| Control | 13·311 (13·213, 13·409) | 0·030 (-0·099, 0·158) | 13·311 (13·216, 13·406) | 0·026 (-0·100, 0·153) | 13·325 (13·230, 13·420) | 0·027 (-0·096, 0·151) |
| Hearing Intervention | 13·341 (13·258, 13·424) | p = 0·65 | 13·338 (13·257, 13·418) | p = 0·68 | 13·352 (13·272, 13·433) | p = 0·66 |
| **Phosphorylated tau 217** | | | | | | |
| Control | 11·543 (11·456, 11·629) | 0·046 (-0·067, 0·159) | 11·553 (11·467, 11·639) | 0·049 (-0·063, 0·161) | 11·562 (11·476, 11·649) | 0·049 (-0·061, 0·158) |
| Hearing Intervention | 11·589 (11·512, 11·665) | p = 0·43 | 11·602 (11·526, 11·678) | p = 0·39 | 11·611 (11·536, 11·686) | p = 0·39 |
| **Phosphorylated tau 231** | | | | | | |
| Control | 13·566 (13·462, 13·670) | 0·017 (-0·123, 0·156) | 13·565 (13·462, 13·668) | 0·020 (-0·118, 0·158) | 13·580 (13·478, 13·682) | 0·021 (-0·114, 0·155) |
| Hearing Intervention | 13·582 (13·489, 13·676) | p = 0·81 | 13·585 (13·494, 13·676) | p = 0·78 | 13·601 (13·510, 13·691) | p = 0·76 |

Abbreviations: ACHIEVE, Aging and Cognitive Health Evaluation in Elders Trial; ARIC, Atherosclerosis in Communities Cohort; CI, confidence interval; NULISA protein quantification, NPQ.

Point estimates, 95% confidence intervals, and p values were calculated by fitting separate linear regression models for each outcome to complete case data. The first model adjusted for the time in years between randomisation and the follow-up assessment. The second model added baseline measures of age, sex, recruitment source, field site, education, hearing loss (pure tone average <40 dB vs ≥40 dB), and the presence of apolipoprotein E ε4 alleles. The third model integrated baseline measures of body mass index and kidney disease. Phosphorylated tau at threonine 181, 217, and 231 was measured using the Alamar NUcleic acid Linked Immuno-Sandwich Assay (NULISA™) central nervous system 120 kit, which produces sample-specific NPQ units. Higher scores indicate greater Alzheimer's disease pathology. A 1-unit change corresponds to a 2-fold increase in the concentration of the biomarker. A 2-unit change corresponds to a 4-fold increase in the concentration. Concentrations are biomarker-specific and cannot be compared to each other.

# **eTable 28.** Multivariate **Intention-to-Treat Analysis of Differences in Exploratory Blood-Based Biomarkers of Alzheimer's Disease Pathology Among Participants with Follow-Up Samples in ACHIEVE by Randomly Assigned Treatment and Recruitment Source (n=552)**

|  | **Model 1** | | **Model 2** | | **Model 3** | |
| --- | --- | --- | --- | --- | --- | --- |
|  | **Value** | **Difference** | **Value** | **Difference** | **Value** | **Difference** |
|  | **β (95% CI)** | **β (95% CI)** | **β (95% CI)** | **β (95% CI)** | **β (95% CI)** | **β (95% CI)** |
|  | **Total (n=552)** | | | | | |
| **Imputed** | | | | | | |
| Control | 0·077 (-0·037, 0·191) | -0·108 (-0·267, 0·051) | 0·080 (-0·031, 0·191) | -0·114 (-0·270, 0·042) | 0·082 (-0·027, 0·192) | -0·118 (-0·271, 0·035) |
| Hearing Intervention | -0·031 (-0·142, 0·080) | p = 0·18 | -0·034 (-0·142, 0·074) | p = 0·15 | -0·036 (-0·141, 0·070) | p = 0·13 |
| **Complete case** | | | | | | |
| Control | 0·077 (-0·037, 0·191) | -0·097 (-0·257, 0·062) | 0·085 (-0·030, 0·200) | -0·067 (-0·228, 0·094) | 0·095 (-0·018, 0·208) | -0·076 (-0·234, 0·082) |
| Hearing Intervention | -0·020 (-0·132, 0·091) | p = 0·23 | 0·018 (-0·093, 0·128) | p = 0·41 | 0·019 (-0·090, 0·128) | p = 0·36 |
|  | **ARIC (n=176)** | | | | | |
| **Imputed** | | | | | | |
| Control | 0·213 (0·035, 0·391) | -0·440 (-0·707, -0·173) | 0·152 (-0·028, 0·332) | -0·450 (-0·716, -0·185) | 0·162 (-0·017, 0·340) | -0·464 (-0·722, -0·206) |
| Hearing Intervention | -0·227 (-0·447, -0·006) | p = 0·0012 | -0·298 (-0·540, -0·056) | p = 0·0009 | -0·302 (-0·539, -0·066) | p = 0·0004 |
| **Complete case** | | | | | | |
| Control | 0·214 (0·036, 0·392) | -0·407 (-0·678, -0·136) | 0·198 (0·011, 0·385) | -0·368 (-0·648, -0·089) | 0·202 (0·017, 0·387) | -0·389 (-0·665, -0·113) |
| Hearing Intervention | -0·193 (-0·418, 0·032) | p = 0·0032 | -0·170 (-0·425, 0·085) | p = 0·0099 | -0·187 (-0·438, 0·064) | p = 0·0057 |
|  | **De Novo (n=376)** | | | | | |
| **Imputed** | | | | | | |
| Control | -0·056 (-0·209, 0·097) | 0·053 (-0·145, 0·250) | -0·047 (-0·192, 0·099) | 0·048 (-0·143, 0·238) | -0·041 (-0·185, 0·102) | 0·053 (-0·134, 0·240) |
| Hearing Intervention | -0·004 (-0·135, 0·128) | p = 0·60 | 0·001 (-0·125, 0·127) | p = 0·62 | 0·012 (-0·112, 0·136) | p = 0·58 |
| **Complete case** | | | | | | |
| Control | -0·056 (-0·209, 0·097) | 0·053 (-0·145, 0·250) | -0·049 (-0·198, 0·100) | 0·058 (-0·137, 0·253) | -0·030 (-0·178, 0·118) | 0·059 (-0·132, 0·249) |
| Hearing Intervention | -0·004 (-0·135, 0·128) | p = 0·60 | 0·009 (-0·120, 0·137) | p = 0·56 | 0·028 (-0·099, 0·155) | p = 0·55 |

Abbreviations: ACHIEVE, Aging and Cognitive Health Evaluation in Elders Trial; ARIC, Atherosclerosis in Communities Cohort; CI, confidence interval.

Point estimates, 95% confidence intervals, and p values were calculated by fitting a single linear regression model for phosphorylated tau at threonine 181, 217, and 231. The first model adjusted for the time in years between randomisation and the follow-up assessment. The second model added baseline measures of age, sex, recruitment source, field site, education, hearing loss (pure tone average <40 dB vs ≥40 dB), and the presence of apolipoprotein E ε4 alleles. The third model integrated baseline measures of body mass index and kidney disease. Phosphorylated tau at threonine 181, 217, and 231 was measured using the Alamar NUcleic acid Linked Immuno-Sandwich Assay (NULISA™) central nervous system 120 kit. Higher scores indicate greater Alzheimer's disease pathology. To prevent outliers from biasing results, a rank-based inverse normal transformation was applied to each biomarker prior to statistical analysis.

# **eFigure 1. ACHIEVE Screening, Randomisation, Follow-Up, and Analysis of Blood-Based Biomarkers Stratified by Recruitment Source**

Abbreviations: ACHIEVE, Aging and Cognitive Health Evaluation in Elders Trial; ARIC, Atherosclerosis in Communities Cohort.

*Among de novo participants, samples were collected and analysed at the follow-up.

# **eFigure 2. Analysis of** Blood-Based Biomarkers Obtained at Follow-Up from **ACHIEVE Participants Stratified by Randomisation and Recruitment Source**

Abbreviations: ACHIEVE, Aging and Cognitive Health Evaluation in Elders Trial; ARIC, Atherosclerosis in Communities Cohort.

# **eFigure 3. Scatterplots of Three-Year Change in Pre-Specified Blood-Based Biomarkers Among Participants with Baseline and Follow-Up Samples in ACHIEVE by Randomly Assigned Treatment (n=164)**

Abbreviations: ACHIEVE, Aging and Cognitive Health Evaluation in Elders Trial.

Glial fibrillary acidic protein and neurofilament light chain were measured using the Alamar NUcleic acid Linked Immuno-Sandwich Assay (NULISA™) central nervous system 120 kit. Higher scores indicate greater neuroinflammation or neurodegeneration. To prevent outliers from biasing results, a rank-based inverse normal transformation was applied to each biomarker.

# **eFigure 4. Scatterplots of Three-Year Change in Exploratory Blood-Based Biomarkers of Alzheimer's Disease Pathology Among Participants with Baseline and Follow-Up Samples in ACHIEVE by Randomly Assigned Treatment (n=164)**

Abbreviations: ACHIEVE, Aging and Cognitive Health Evaluation in Elders Trial.

Phosphorylated tau at threonine 181, 217, and 231 was measured using the Alamar NUcleic acid Linked Immuno-Sandwich Assay (NULISA™) central nervous system 120 kit. Higher scores indicate greater Alzheimer's disease pathology. To prevent outliers from biasing results, a rank-based inverse normal transformation was applied to each biomarker.

# **eFigure 5. Scatterplots of Three-Year Change in Untransformed Pre-Specified Blood-Based Biomarkers Among Participants with Baseline and Follow-Up Samples in ACHIEVE by Randomly Assigned Treatment (n=164)**

Abbreviations: ACHIEVE, Aging and Cognitive Health Evaluation in Elders Trial.

Glial fibrillary acidic protein and neurofilament light chain were measured using the Alamar NUcleic acid Linked Immuno-Sandwich Assay (NULISA™) central nervous system 120 kit. This assay produces sample-specific NULISA protein quantification through normalisation using internal and inter-plate controls followed by a log2-transformation. Higher scores indicate greater neuroinflammation or neurodegeneration. A 1-unit change corresponds to a 2-fold increase in the concentration of the biomarker. A 2-unit change corresponds to a 4-fold increase in the concentration.

# **eFigure 6. Scatterplots of Three-Year Change in Untransformed Exploratory Blood-Based Biomarkers of Alzheimer's Disease Pathology Among Participants with Baseline and Follow-Up Samples in ACHIEVE by Randomly Assigned Treatment (n=164)**

Abbreviations: ACHIEVE, Aging and Cognitive Health Evaluation in Elders Trial.

Phosphorylated tau at threonine 181, 217, and 231 was measured using the Alamar NUcleic acid Linked Immuno-Sandwich Assay (NULISA™) central nervous system 120 kit. Higher scores indicate greater Alzheimer's disease pathology. A 1-unit change corresponds to a 2-fold increase in the concentration of the biomarker. A 2-unit change corresponds to a 4-fold increase in the concentration.

# **Members of the ACHIEVE Collaborative Research Group**

**Members of the ACHIEVE Collaborative Research Group are:**

**Johns Hopkins University:** Jennifer A. Deal, PhD (MPI), Frank R. Lin, MD, PhD, Marilyn Albert, PhD, Joshua Betz, MS, Alden Gross, PhD, Christine M. Mitchell, ScM, Clarice Myers, AuD, Jennifer A. Schrack, PhD, Richey Sharrett, MD, DrPH

**New York University:** Josef Coresh, MD, PhD (MPI), James R. Pike, MBA, Nicholas S. Reed, AuD, Alison R. Huang, PhD

**George W. Comstock Center for Public Health Research and Prevention, Johns Hopkins University:** Melissa Minotti, MPH, Spencer Bolton, Laura Sherry, AuD

**University of Minnesota:** James S. Pankow, PhD (Site PI), Sarah Aguilar, MS, Elizabeth Anderson, PhD, Sydney Boelter, Elizabeth Penland Miller, Debbie Ng, MPH, Kristi Oeding, PhD, AuD, Sandra Potter, Katherine Teece, AuD, Soni Uccellini, Matthew Waggenspack, AuD, Luanne Welch, RN, Jacqueline Weycker, AuD, Kerry Witherell, AuD, CCC-A

**University of Mississippi Medical Center:** Kevin Sullivan (Site PI), Thomas Mosley, PhD, Latilraka Anderson, MS, EdS, Jillian Burt, MBA, April Carr, Arkenya Carter, MS, Sarah Faucette, AuD PhD, Rachel Foster, MS, Ceola Greenwood, MS, Temeka Griffin, DPC, Candace Jones, MSW, EdS, Dawn McLendon, LPN, Thomas Mosley, PhD, Stacee Naylor MSN, RN, CCRP, Jenny Newman, MS, Deidre O’Connor, MS, Tiffany Owens, MSCP, Jeraline Sims, RN, MSN, Allison Thweatt, RN, Tamikia Washington

**Wake Forest University:** Lynne E. Wagenknecht, DrPH (Site PI), Kathleen M. Hayden, PhD, Bria Backman, Debbie Barr, Joshua Evans, Jaime Hampton, AuD, Hailley Humphrey-Rutledge, AuD, Kaila H. Liou, AuD, Ashely Mitchell, Susan Smith, Nadine Shelton

**University of South Florida:** Theresa Chisolm, PhD (Site PI), Victoria Sanchez, AuD, PhD, Michelle Arnold, AuD, PhD, Ann C. Eddins, PhD, MBA, Emily Moore, AuD, Haley Neil, AuD, Preyanca Oree, AuD, Laura Westermann, MA

**University of Pittsburgh:** Nancy W. Glynn, PhD (Site PI), Yurun Cai, PhD, Theresa Gmelin, MSW, MPH

**University of North Carolina (ACHIEVE Data Coordinating Center):** David Couper, PhD (Site PI), Sheila Burgard, MS, Lisa Gravens-Mueller, MS, David Li, MS

**Edinburgh Napier University:** Adele M. Goman PhD

**Mayo Clinic:** Clifford R. Jack, MD (Site PI), David Knopman, MD, Denise Reyes, AJ Spychalla, Kaely Thostenson

**Data and Safety Monitoring Board (DSMB):** Doug Galasko, PhD (Chair), Julie Buring, PhD, Judy Dubno, PhD, Tom Greene, PhD, Lawrence Lustig, MD

**National Institute on Aging (NIA):** Coryse St. Hillaire-Clarke, PhD, Matt Sutterer, PhD

# **References**

1. Panikkar D, Vivek S, Crimmins E, Faul J, Langa KM, Thyagarajan B. Pre-analytical variables influencing stability of blood-based biomarkers of neuropathology. *J Alzheimers Dis* 2023; **95**: 735–748.
2. Ishigami J, Karger AB, Lutsey PL, Coresh J, Matsushita K. Stability of serum bone-mineral, kidney, and cardiac biomarkers after a freeze-thaw cycle: the ARIC Study. *Am J Epidemiol* 2022; **191**: 534–537.
3. Selvin E, Coresh J, Jordahl J, Boland L, Steffes MW. Stability of haemoglobin A1c (HbA1c) measurements from frozen whole blood samples stored for over a decade. *Diabet Med* 2005; **22**: 1726–1730.
4. Rea Reyes RE, Wilson RE, Langhough RE, Studer RL, Jonaitis EM, Oomens JE, et al. Targeted proteomic biomarker profiling using NULISA in a cohort enriched with risk for Alzheimer’s disease and related dementias. *Alzheimers Dement* 2025; **21**: e70166.
5. Kolberg ER, Morales EEG, Thallmayer TW, Arnold ML, Burgard S, Chisolm TH, et al.; ACHIEVE Collaborative Research Group. Hearing loss and cognition: a protocol for ensuring speech understanding before neurocognitive assessment. *Alzheimers Dement* 2024;**20**:1671–81.
6. Wechsler D. Wechsler Memory Scale–Revised. San Antonio, TX: Psychological Corporation, 1987.
7. Williams B, Mack W, Henderson V. Boston Naming Test in Alzheimer’s disease. *Neuropsychologia* 1989; **27**: 1073–1079.
8. Benton A, Hamsher K. Multilingual Aphasia Examination. Iowa City, IA: University of Iowa, 1976.
9. Reitan R. Validity of the Trail Making Test as an indicator of organic brain damage. *Percept Mot Skills* 1958; **8**: 271–276.
10. Ryan J, Lopez S. Wechsler Adult Intelligence Scale-III. In: Dorfman W, Hersen M, eds. Understanding Psychological Assessment: Perspectives on Individual Differences. New York, NY: Kluwer Academic/Plenum Publishers, 2001.
11. Knopman D, Ryberg S. A verbal memory test with high predictive accuracy for dementia of the Alzheimer type. *Arch Neurol* 1989; **46**: 141–145.
12. Killion M, Niquette P, Gudmundsen G, Revit L, Banerjee S. Development of a quick speech-in-noise test for measuring signal-to-noise ratio loss in normal-hearing and hearing-impaired listeners. *J Acoust Soc Am* 2004; **116**: 2395–2405.
13. Tomioka K, Ikeda H, Hanaie K, et al. The Hearing Handicap Inventory for Elderly-Screening (HHIE-S) versus a single question: reliability, validity, and relations with quality of life measures in the elderly community, Japan. *Qual Life Res* 2013; **22**: 1151–1159.
14. Ventry I, Weinstein B. The Hearing Handicap Inventory for the Elderly: a new tool. *Ear Hear* 1982; **3**: 128–134.
15. Rawlings A, Sang Y, Sharrett A, et al. Multiple imputation of cognitive performance as a repeatedly measured outcome. *Eur J Epidemiol* 2017; **32**: 55–66.
16. Van Buuren S. Multiple imputation of discrete and continuous data by fully conditional specification. *Stat Methods Med Res* 2007; **16**: 219–242.
17. von Hippel P. How many imputations do you need? A two-stage calculation using a quadratic rule. *Sociol Methods Res* 2020; **49**: 699–718.
18. Collins L, Schafer J, Kam C. A comparison of inclusive and restrictive strategies in modern missing data procedures. *Psychol Methods* 2001; **6**: 330–351.
19. Detry M, Lewis R. The intention-to-treat principle: how to assess the true effect of choosing a medical treatment. *JAMA* 2014; **312**: 85–86.
20. Little R, Rubin D. Causal effects in clinical and epidemiological studies via potential outcomes: concepts and analytical approaches. *Annu Rev Public Health* 2000; **21**: 121–145.
21. Shrier I, Steele R, Verhagen E, Herbert R, Riddell C, Kaufman J. Beyond intention to treat: what is the right question? *Clin Trials* 2014; **11**: 28–37.
22. Hernán M, Robins J. Per-protocol analyses of pragmatic trials. *N Engl J Med* 2017; **377**: 1391–1398.
23. Jo B, Stuart E. On the use of propensity scores in principal causal effect estimation. *Stat Med* 2009; **28**: 2857–2875.
24. Herbert R, Kasza J, Bø K. Analysis of randomised trials with long-term follow-up. *BMC Med Res Methodol* 2018; **18**: 48.
